# Use of contraceptives and risk of inflammatory bowel disease: a nested case–control study

**DOI:** 10.1111/apt.16647

**Published:** 2021-10-18

**Authors:** Thomas Joshua Pasvol, Stuart Bloom, Anthony Walter Segal, Greta Rait, Laura Horsfall

**Affiliations:** 1 The Research Department of Primary Care and Population Health, University College London, London, UK; 2 University College London Hospitals NHS Foundation Trust, London, UK; 3 Division of Medicine, University College London UK, London, UK

## Abstract

**Background:**

How contraceptive formulation, dose, duration of therapy and mode of delivery affects the risk of inflammatory bowel disease (IBD) is poorly described.

**Aim:**

To examine associations between types of hormonal contraception and development of IBD.

**Methods:**

This was a nested case-control study using IQVIA Medical Research Data. Women aged 15-49 years with a new diagnosis of IBD were matched with up to six controls by age, practice and year. Odds ratios (OR) and 95% confidence intervals (95% CI) for incident IBD and use of contraception were calculated.

**Results:**

4932 incident cases of IBD were matched to 29 340 controls. Use of combined oral contraceptive pills (COCPs) was associated with the development of Crohn's disease and ulcerative colitis (OR 1.60 [1.41-1.82] and 1.30 [1.15-1.45], respectively). Each additional month of COCP exposure per year of follow-up increased risk of Crohn's disease by 6.4% (5.1%-7.7%) and ulcerative colitis by 3.3% (2.1%-4.4%). Progestogen-only pills had no effect on Crohn's disease risk (OR 1.09 [0.84-1.40]) but there was a modest association with ulcerative colitis (OR 1.35 [1.12-1.64]). Parenteral contraception was not associated with the development of Crohn's disease or ulcerative colitis (OR 1.15 [0.99-1.47] and 1.17 [0.98-1.39], respectively).

**Conclusions:**

We observed an increase in the risk of IBD with increasing duration of exposure to COCPs. Progestogen-only pills were not associated with Crohn's disease but there was a modest association with ulcerative colitis. There was no association between parenteral progestogen-only contraception and IBD. These findings are broadly consistent with a hypothesis that the oestrogen component of contraception may drive IBD pathogenesis.

## 1 Introduction

Changes in the epidemiology of inflammatory bowel disease (IBD) across geographical location and time suggest that environmental risk factors play a major role in disease development.^1^

In the UK, approximately 26% of women of reproductive age use hormonal contraception^2^ and combined oral contraceptive pills (COCPs) which work by releasing an oestrogen and a progestogen are the most popular method. An increased risk of development of IBD in association with oral contraceptive pill exposure has been shown in numerous studies.^3–5^ However, the precise biological mechanism remains unknown. A number of proposed theories exist, largely relating to the effect of exogenous oestrogen on immunomodulation, intestinal wall function, gut microbiome and hypercoagulability.

Oestrogen has been linked to inhibition of TH1 mediated cytokines and stimulation of TH2 mediated cytokines.^6^ Additionally, oestrogen has been implicated in the pathogenesis and disease progression in a number of TH2 mediated inflammatory conditions.^7,8^ This would support a relationship between exogenous oestrogens and development of UC but not CD.

It is established that exogenous oestrogen affects oral and vaginal microbiota.^9,10^ However, more recent research has implicated the oestrogen-gut microbiome axis as playing a crucial role in the pathogenesis of several oestrogen-mediated diseases.^11^ If a complex relationship exists between oestrogen levels and the gut microbiome then one could hypothesise that changing circulating levels of oestrogen may, in turn, disrupt gut flora and precipitate gastrointestinal disease.

Oestrogen has been shown to modulate intestinal wall barrier function^12,13^ and individuals who have an episode of bacterial gastroenteritis have been shown to be fourfold more likely to develop IBD in the following year.^14^ Therefore, if the barrier function of the intestinal wall is compromised by exogenous oestrogen then this may potentially increase the risk of triggering IBD in a genetically susceptible individual. Additionally, some enteric infections can be sexually acquired and one could hypothesise that women taking contraception may be at greater risk of exposure.^15^

Some have theorised that IBD development may be related to micro-ischaemia within the vasculature of the gut^16^ and it is established that COCPs are associated with thromboembolic disease.^17^

How hormone formulation, dose and duration of contraceptive pill exposure relate to IBD risk is poorly characterised. Additionally, there is a paucity of literature on how progestogen-only and parenteral preparations of contraception affect IBD risk.

We hypothesise that oestrogen-containing contraceptives are associated with an increased risk of IBD and progestogen-only methods are not. We aimed to examine the association between various types of contraception and development of IBD. We were particularly interested in the impact of hormone formulation, dose and duration of therapy on subsequent IBD.

## 2 Materials and Methods

### 2.1 Data source

IQVIA Medical Research Data (IMRD) (incorporating data supplied by The Health Improvement Network, a Cegedim SA Database. Reference made to The Health Improvement Network is intended to be descriptive of the data asset licensed by IQVIA) is a large longitudinal database containing the anonymised electronic medical records of 18.3 million patients from 797 general practices throughout the UK. Data in IMRD are based on patient consultation records and are recorded using the Read code hierarchical coding system.^18^ The GP practices in IMRD are broadly representative of the UK in terms of age and gender of patients, practice size, geographical distribution, smoking prevalence^19,20^ and the prevalence of numerous chronic conditions such as hypertension, asthma and diabetes.^21^

Not only has the diagnosis of IBD been validated in a similar GP database,^22^ but we have demonstrated that 98.2% of individuals coded for incident IBD in IMRD have a record of at least one additional clinical event supportive of the diagnosis with 87.7% having at least two supporting events.^23^ Clinical events included a prescription for IBD drugs (any aminosalicylate or rectal steroid enema listed in chapter 1.5 of the British National Formulary,^24^ azathioprine, mercaptopurine, methotrexate, ciclosporin, infliximab, adalimumab, ustekinumab or vedolizumab [supplementary code lists]), a presentation with symptoms in keeping with IBD (abdominal pain, diarrhoea, bloody stools, weight loss), a referral to a gastroenterologist or an endoscopy.

### 2.2 Study population

A cohort of women aged 15-49 years who were registered with study practices contributing to IMRD for the period 1 January 2000-31 December 2018 was identified. Women were required to be registered with the practice for at least 9 months prior to cohort entry to avoid misclassifying prevalent IBD as incident disease.^27^ GP practices were required to meet acceptable standards of electronic data quality prior to cohort entry.^25,26^ Women were censored from the cohort at the first recording of a condition which would usually preclude future contraceptive use (bilateral salpingo-oophorectomy, hysterectomy, sterilisation) or the first prescription of hormone replacement therapy (supplementary code lists).

Within the cohort, we designed two nested case–control studies, one for CD and one for UC. Cases were those diagnosed with incident CD or UC during study follow-up. Case definition was taken from our previously published incidence study of IBD; In order to qualify as a case, an individual had to have either (a) two codes for IBD at different time points, (b) one code for IBD plus one prescription for a drug commonly used to treat IBD^23^ (supplementary code lists). Eligible cases were required to have at least one year of prescribing history prior to the date of diagnosis. One year was selected because prescriptions for contraceptive pills are typically not longer than one year in length.

Each case was matched with up to six controls by year of birth and GP practice using incidence density sampling. Each control was allocated an index date which was the date of diagnosis for their matched case. Each control was required to have the same (or greater) prescribing history prior to the index date as their matched case. Any additional prescribing history that a control may have had did not contribute towards the analysis (ie all controls contributed the same amount of prescribing history as their matched cases over the same calendar period). The lookback period was defined as the period between the start of the prescribing history and the IBD diagnosis date (or matched index date for controls).

### 2.3 Exposures

Exposure to contraceptives was based on the total lookback period. COCPs were subdivided by pill generation. Pill generation is the standard four-level classification system used for COCPs as they were rolled out chronologically, first-generation pills being the oldest and fourth generation the newest. Most pills contain ethinylestradiol and the difference between the generations of pill is the type of progestogen that is included. First-generation pills were not included as they had all been discontinued by the early 1990s. Co-cyprindiol, a pill containing ethinylestradiol and cyproterone acetate which is used as a treatment for acne and as a contraceptive was also included.

For the primary analysis, women were categorised as either non-contraceptive users (no prescribed contraceptive use during the lookback period), second-generation COCP users, newer generation COCP users (including third-generation and fourth-generation COCPs in addition to co-cyprindiol), POP users, long-acting reversible contraception users (these are parenteral progestogen-only methods including intrauterine systems, contraceptive implants and contraceptive intramuscular injections) or mixed contraceptive users (any combination of contraceptives during the lookback period) (supplementary code lists).

Specifically for contraceptive pills, women were classed as current users if their most recent prescription would finish ≤28 days before (or after) the index date. Twenty-eight days was selected because contraceptive pills come in boxes which last 28 days. COCPs were subdivided by oestrogen content; low strength (<30 μg ethinylestradiol) and standard strength (≥30 μg ethinylestradiol). For those pills containing mestranol, we treated 50 μg mestranol as bioequivalent to 35 μg ethinylestradiol.^28^ For those pills-containing estradiol, we treated 200 μg estradiol as bioequivalent to 1 μg ethinylestradiol.^29,30^

“Average months of contraceptive pill exposure per year of follow-up” was calculated and treated as both a continuous variable and separately as categorical variable in quantiles of three months per year to check for evidence of non-linearity with the development of IBD. This was done separately for COCPs and POPs. We also calculated “Average daily dose of oral oestrogen over follow-up” and similarly analysed as both a continuous variable and a categorical variable in quantiles of 5 μg ethinylestradiol per day (or equivalent).

### 2.4 Covariates and confounding factors

History of endometriosis, acne and polycystic ovarian syndrome were included as covariates because they are all commonly treated with COCPs and are also potentially linked to the development of IBD (supplementary code lists); increased risk of IBD has been shown in women with endometriosis in a nationwide Danish cohort study,^31^ severe acne can be a feature of IBD^32,33^ and polycystic ovarian syndrome has been shown to be associated with reduced biodiversity in the gut microbiome.^34^

We adjusted for smoking status treating smoking as a categorical variable with the levels “never smoker,” “ex-smoker” and “current smoker” (supplementary code lists). Smoking was included as it is an established risk factor for CD and may decrease the risk of developing UC.^35^ Additionally, smoking is a relative contraindication to the prescription of COCPs.^36,37^

We adjusted for body mass index (BMI) as a categorical variable using the levels “underweight” (BMI <18), “normal weight” (BMI 18-25), “overweight” (BMI 25-30) and “obese” (over 30) for the primary analysis and as a continuous variable in a sensitivity analysis. BMI was included as CD often presents with weight loss and BMI is an important factor to consider when choosing appropriate contraception.^37^

Social deprivation as measured by Townsend score^38^ was included as we found there to be an association between Townsend score and risk of UC in a previous study.^23^ Additionally, contraceptive uptake is lower in more deprived socio-economic groups.^39^ Evidence of pregnancy during follow-up was included as a yes/no binary variable; pregnancy would usually preclude the use of contraception and women may be less likely to conceive if they are unwell and developing a chronic inflammatory illness (supplementary code lists).

Data on BMI and smoking were captured using the earliest value recorded during the lookback period. If data were missing during this period then the latest value recorded prior to the start of the look-back period was substituted.

### 2.5 Statistical analysis

Crude incidence estimates per 100 000 person-years at risk were calculated for the source cohort. Ninety-five per cent confidence intervals (95% CI) were then calculated assuming a Poisson distribution.

Conditional logistic regression was used to analyse the nested-case control studies and obtain odds ratios (OR) for each exposure with 95% CI. The Wald test was used to test for the significance of exposures and categorical variables in the regression model and to test for multiplicative interactions. We were particularly interested in an interaction between contraceptive pill exposure and smoking as it was reported in a large cohort study that the increased risk of UC with contraceptive pills was exclusive to smokers.^40^ To check for secular trends we stratified ORs for OCP exposure by calendar period of IBD diagnosis date/index date using five-yearly quantiles.

Missing data were dealt with using complete case analysis for continuous variables and including “missing” as a level to categorical variables.

StataCorp. 2017. Stata Statistical Software: Release 15. College Station, TX: StataCorp LLC was used for all analyses.

### 2.6 Ethics

IMRD data collection was approved by the NHS South-East Multicentre Research Ethics Committee in 2003. This study was approved by the Scientific Research Committee (SRC) on 29 September 2018 (SRC reference 18THIN082).

### 2.7 Patient and public involvement

We involved representatives from the University College Hospitals NHS Foundation Trust IBD patient panel in refining the research question and designing the study protocol.

## 3 Results

A source cohort of 3 202 575 women contributing 16 300 866 person-years of follow-up was identified. Median (IQR) age at cohort entry was 28.2 (21.1-36.1) years. Overall incidence was 14.7 (95% CI 14.1-15.3) and 17.8 (95% CI 17.2-18.5) per 100 000 person-years for CD and UC, respectively.

2231 incident cases of CD were matched to 13 279 controls and 2701 incident cases of UC were matched to 16 061 controls (Table 1). Median (IQR) lookback period was 5.4 (3.0-8.7) years in the CD study and 5.2 (2.9-8.8) years in the UC study.

Amongst the 4932 IBD cases, 4917 (99.7%) had at least one additional event supportive of the diagnosis recorded in the GP notes (a prescription for IBD drugs, gastrointestinal symptoms in keeping with IBD, a referral to a gastroenterologist, an endoscopy) with 4642 (94.1%) having at least two supporting events.

### 3.1 Crohn's disease

**Figure 1:**
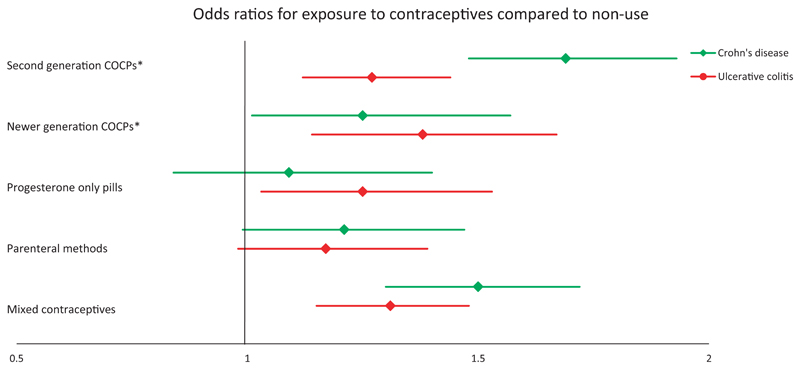
Adjusted odds ratios for Crohn's disease and ulcerative colitis exposed to contraceptives compared with non-use. Odds ratios with 95% confidence intervals are adjusted for Townsend score, body mass index, smoking status and history of polycystic ovarian syndrome, endometriosis, acne and pregnancy. *Abbreviation: COCP, combined oral contraceptive pill

Use of COCPs was associated with an increased risk of CD (OR 1.60 [95% CI 1.41-1.82]). The increased risk was higher for second-generation COCPs than newer COCPs when compared to non-use (OR 1.69 [95% CI 1.48-1.93] vs 1.25 [95% CI 1.01-1.57], respectively) (Figure 1, Table S1). The risk of CD was increased further amongst current users of COCPs (OR 2.12 [95% CI 1.83-2.44] and 1.64 [95% CI 1.33-2.01] for second-generation and newer COCPs, respectively). However, amongst current COCP users, there was no difference in CD risk for those using low strength oestrogen pills compared to standard strength oestrogen pills (OR 1.16 [95% CI 0.74-1.80]). Use of POPs and parenteral contraceptive methods was not associated with an increased risk of CD compared to non-use (OR 1.09 [95% CI 0.84-1.40] and 1.15 [95% CI 0.99-1.47], respectively; Figure 1, Table S1).

**Figure 2:**
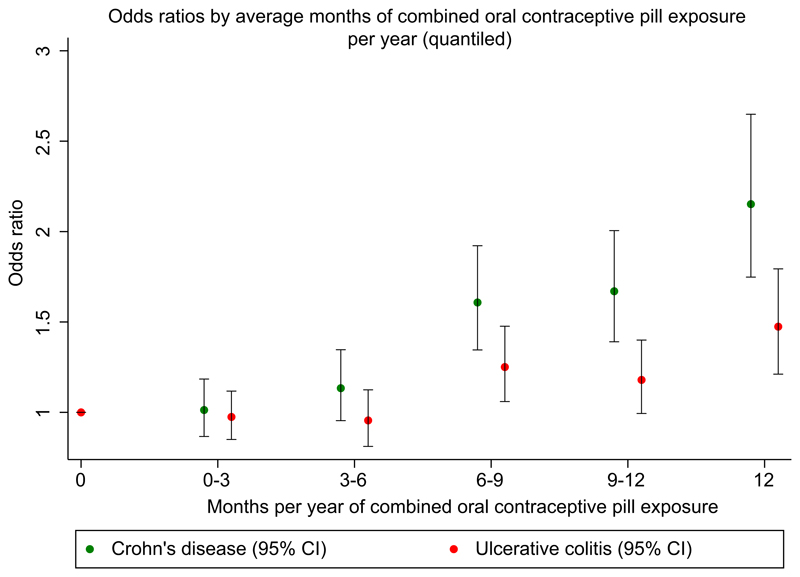
Adjusted odds ratios for Crohn's disease and ulcerative colitis exposed to COCPs compared to non-use. Average months per year of exposure to COCPs are stratified in 3-monthly quantiles. Odds ratios and 95% confidence intervals are adjusted for Townsend score, body mass index, smoking status and history of polycystic ovarian syndrome, endometriosis, acne and pregnancy

The risk of CD went up with increasing duration of exposure to COCPs (Figure 2). When treating “average months of COCP exposure per year” as a continuous linear variable, each additional month per year of COCP exposure, increased risk of CD by 6.4% (95% CI 5.1-7.7) compared to non-users. When treating average daily dose of oral oestrogen over follow-up as a continuous linear variable, CD risk increased by 3.1% (95% CI 2.5-3.7) per μg/day of ethinylestradiol (or equivalent) compared to non-users. Longer durations of exposure to POPs had no effect on CD risk (OR 0.99 [95% CI 0.97-1.02]). We found no evidence of an interaction between smoking and contraceptive pill exposure on risk of CD (Tables S2-S4). We found no evidence of temporal changes in the relationship between OCP exposure and CD (Table S5).

### 3.2 Ulcerative colitis

We found use of all types of contraceptive pills to be associated an increase in risk of UC; OR 1.27 (95% CI 1.12-1.44) for second-generation COCPs, 1.38 (95% CI 1.14-1.67) for newer generation COCPs and 1.25 (95% CI 1.03-1.53) for POPs, with risk increasing slightly amongst current users; OR 1.48 (95% CI 1.29-1.69) for second-generation COCPs, 1.62 (95% CI 1.34-1.95) for newer generation COCPs and 1.35 (95% CI 1.12-1.64) for POPs. Amongst current COCP users, there was no difference in UC risk for those using low strength oestrogen pills compared to standard strength oestrogen pills (OR 1.33 [95% CI 0.81-2.18]). Parenteral methods had no effect on UC risk (OR 1.17 [95% CI 0.98-1.39]) (Figure 1, Table S1).

When treating “average months of COCP exposure per year” as a continuous linear variable, each additional month per year of COCP exposure, increased risk of UC by 3.3% (95% CI 2.1-4.4) compared to non-users (Figure 2), equating to an additonal 1.7% (95% CI 1.1-2.2) increase in risk per μg/day of ethinylestradiol (or equivalent). However, a similar dose-response relationship was not observed with POPs (OR 1.02 [95% CI 1.00-1.04]). No interaction was found between POP exposure and smoking (Table S2). However, we found that the development of UC was slightly more associated with non-smokers taking COCPs than smokers taking COCPs (*P* = 0.03) (Table S3). We found no evidence of temporal changes in the relationship between OCP exposure and UC (Table S5).

### 3.3 Sensitivity analysis

When treating BMI as a continuous variable and excluding those with missing BMI, results were similar to the primary analysis across all methods of contraception for both CD and UC. However, confidence intervals were wider and crossed the null value for CD and newer generation COCPs (Table S6).

## 4 Discussion

This is the first study to describe IBD diagnosis in relation to a range of different contraceptives including progestogen-only methods. We observed an increase in the risk of CD with increasing durations of exposure to COCPs but not to POPs. We observed a more modest increase in the risk of UC with exposure to COCPs and POPs. There was no association between the use of parenteral progestogen-only contraception and IBD. Although there were inconsistencies, these findings are broadly in accordance with the hypothesis that exogenous oral oestrogen is the component of contraception associated with development of IBD.

Study strengths include the large number of included cases and controls and the use of a database which has been shown to be generalisable to the UK population. Unlike other studies which have relied on self-reporting of historic contraceptive use which is a potential source of recall bias, our data is based on prospectively collected electronic prescribing records which include detailed information on treatment duration, formulation and dosage. In comparison to other case–control studies, where controls have been peer-nominated or recruited from clinic, all women aged 15-49 years from IMRD were eligible for inclusion, thus minimising selection bias.

Our study has a number of limitations. Firstly, the potential mis-classification of exposure. Although the vast majority of women in the UK obtain contraception from primary care, our study does not capture those contraceptives obtained from sexual and reproductive health services. In the UK, 5% of females aged 13-54 years used a sexual and reproductive health service for reasons of contraception between 1 April 2019 and 31 March 2020.^41^ Additionally, although IMRD includes detailed prescribing data, we were unable to capture information on patient adherence. It has been reported that up to 52% of women miss their contraceptive pill once or more per month with 14% missing twice or more per month.^42^ These factors could potentially result in a shift in the ORs towards unity and an underestimate in the effect of contraceptives on IBD risk. Secondly, BMI and smoking data was unavailable for a slightly larger proportion of controls than cases (Table 1). Thirdly, although our sample size was large, we lacked statistical power to analyse newer classes of COCPs separately; third generation, fourth generation and co-cyprindiol were grouped together. Fourthly, we were not able to confirm our cases with radiological, endoscopic or histological findings. Therefore, it is possible that a small number were misclassified. Finally, although a validation paper has shown that median time between IBD diagnosis and the electronic recording in the primary care records is only eight days,^22^ there are bound to be delays in IBD diagnosis for a number of other reasons such as misdiagnosis or extended wait times for colonoscopy. This could introduce bias if we have included contraceptive exposure after a woman has already developed IBD; being diagnosed with a chronic illness may influence contraceptive uptake.

In keeping with published literature, we found an association between contraceptive pill use and risk of IBD.^3–5^ Our overall ORs for contraceptive pill exposure in relation to IBD were similar to a meta-analysis including 20 studies published in 2017^4^; 1.51 (95% CI 1.34-1.71) vs 1.32 (95% CI 1.17-1.49) for CD and 1.29 (95% CI 1.15-1.44) vs 1.30 (95% CI 1.13-1.49) for UC. In comparison to a smaller nested-case control study from the Asia-Pacific region,^43^ we observed similar ORs for newer generations of OCPs (1.25 [95% CI 1.01-1.57] vs 1.31 [95% CI 0.55-1.99] for CD and 1.38 [95% CI 1.14-1.67] vs 1.20 [95% CI 0.70-1.70] for UC). However, they concluded that these associations were non-significant which could be explained by insufficient study power. In keeping with the small number of previous studies looking at the duration of exposure, we found that the risk of IBD increased with longer periods of exposure. We observed a more than doubling in risk of CD in those taking COCPs continuously throughout follow-up. Contrary to a large US cohort study, we found that the development of UC was slightly more associated with non-smokers taking COCPs.^40^ However, we did not observe this effect for POPs or contraceptive pills overall (Tables S3-S5). As the effect was small, this may represent a chance finding.

No previous studies have looked at IBD risk specifically in relation to progestogen-only contraceptive methods and our finding that increased CD risk was isolated to oestrogen-containing contraception is novel. Of note, a study exploring associations between contraceptive pills and disease outcomes in CD found that there was an increased risk of surgery in those taking COCPs but not progestogen-only methods.^44^ Although we found no difference in IBD risk between users of low strength and standard strength oestrogen-containing COCPs, it should be noted that differences in oestrogen content amongst most COCPs are small (usually containing 20-35 μg ethinyloestradiol or equivalent).

Although a number of studies have associated oestrogens with IBD pathogenesis, genome-wide association studies have not implicated a number of genetic determinants of circulating oestrogen levels (variants in/near CYP19A1, FAM9B, Xq27.3, TRIM4 and CYP11B1/B2)^45^ as at risk loci for IBD^46^ and a mendelian randomisation analysis has found that genetically predicted 17β-estradiol reduced low-grade systemic inflammatory markers in women.^47^ However, it is important to note that COCPs do not work by slightly increasing background levels of endogenous oestrogen, they provide exogenous hormones which have a number of inhibitory effects on the pituitary and hypothalamus to prevent ovulation and anti-androgenic properties.

The benefits of contraceptives greatly outweigh the risk of developing IBD in the vast majority of individuals. However, our results may be useful to those women seeking contraception who have a strong family history of IBD. Importantly, our research does begin to shed some light on the potential biological mechanisms involved in the pathogenesis of these two diseases, highlighting the importance of future studies focusing on specific exogenous sex hormones.

## Supplementary Material

Supp info 1

Supp info 2

Supporting Information Tables

## Figures and Tables

**Table 1:** Patient demographics for cases and controls

	Crohn's disease		Ulcerative colitis	
	Cases (n = 2231) (%)	Controls (n = 13 279) (%)	Cases (n = 2701) (%)	Controls (n = 16 061) (%)
Median age (IQR) (diagnosis/index date)	29.8 (22.9-38.3)	29.8 (22.9-38.3)	33.2 (26.4-40.1)	33.2 (26.4-40.1)
Townsend				
1	412 (18.5)	2670 (20.1)	567 (21.0)	3385 (21.1)
2	382 (17.1)	2353 (17.7)	526 (19.5)	2934 (18.3)
3	401 (17.8)	2387 (18.0)	481 (17.8)	2981 (18.6)
4	354 (15.9)	2096 (15.8)	394 (14.6)	2496 (15.5)
5	268 (12.0)	1522 (11.5)	251 (9.3)	1704 (10.6)
Missing	414 (18.6)	2251 (17.0)	482 (17.9)	2561 (16.0)
Weight				
Median BMI (IQR)	23.6 (21.0-27.6)	23.8 (21.2-27.9)	23.1 (20.8-26.4)	23.9 (21.3-28.0)
Normal weight (BMI 18-25)	1042 (46.7)	5532 (41.7)	1436 (53.2)	7119 (44.3)
Overweight (BMI 25-30)	428 (19.2)	2252 (17.0)	476 (17.6)	3050 (19.0)
Obese (BMI >30)	310 (13.9)	1874 (14.1)	294 (10.9)	2313 (14.4)
Underweight (BMI <17)	81 (3.6)	358 (2.7)	91 (3.4)	380 (2.4)
Missing	370 (16.6)	3263 (24.6)	404 (15.0)	3199 (19.9)
Smoking				
Non-smoker	968 (43.4)	6480 (48.8)	1384 (51.2)	8078 (50.3)
Ex-smoker	238 (10.7)	1112 (8.4)	395 (14.6)	1505 (9.4)
Smoker	690 (30.9)	2980 (22.4)	563 (20.8)	3552 (22.1)
Missing	335 (15.0)	2707 (20.4)	359 (13.3)	2926 (18.2)
Polycystic ovarian syndrome	56 (2.5)	328 (2.5)	58 (2.2)	464 (2.9)
Endometriosis	37 (1.7)	148 (1.1)	35 (1.3)	215 (1.3)
Pregnancy	630 (28.2)	3553 (26.8)	867 (32.1)	4606 (28.7)
Acne	341 (15.3)	2333 (17.6)	491 (18.2)	2710 (16.9)

Abbreviation: BMI, body mass index.

## Data Availability

Data may be obtained from a third party and are not publicly available. Data were obtained from IQVIA Medical Research Data. The authors' licence for using these data does not allow sharing of raw data with third parties. However, the authors are happy to share the code used in this study upon reasonable request. Requesters should email the corresponding author to request the relevant code.

## References

[1] Ananthakrishnan AN, Bernstein CN, Iliopoulos D (2018). Environmental triggers in IBD: a review of progress and evidence. Nat Rev Gastroenterol Hepatol.

[2] Firman N, Palmer MJ, Timaeus IM, Wellings K (2018). Contraceptive method use among women and its association with age, relationship status and duration: findings from the third British National Survey of Sexual Attitudes and Lifestyles (Natsal-3). BMJ Sex Reprod Health.

[3] Cornish JA, Tan E, Simillis C, Clark SK, Teare J, Tekkis PP (2008). The risk of oral contraceptives in the etiology of inflammatory bowel disease: a meta-analysis. Am J Gastroenterol.

[4] Ortizo R, Lee SY, Nguyen ET, Jamal MM, Bechtold MM, Nguyen DL (2017). Exposure to oral contraceptives increases the risk for development of inflammatory bowel disease: a meta-analysis of case-controlled and cohort studies. Eur J Gastroenterol Hepatol.

[5] Wang X, Fan X, Deng H (2019). Use of oral contraceptives and risk of ulcerative colitis – a systematic review and meta-analysis. Pharmacol Res.

[6] Salem ML (2004). Estrogen, a double-edged sword: modulation of TH1- and TH2-mediated inflammations by differential regulation of TH1/TH2 cytokine production. Curr Drug Targets Inflamm Allergy.

[7] Cutolo M, Capellino S, Straub RH (2008). Oestrogens in rheumatic diseases: friend or foe?. Rheumatology.

[8] González DA, Díaz BB, Rodríguez Pérez MDC, Hernández AG, Chico BND, de León AC (2010). Sex hormones and autoimmunity. Immunol Lett.

[9] Muhleisen AL, Herbst-Kralovetz MM (2016). Menopause and the vaginal microbiome. Maturitas.

[10] Brusca MI, Rosa A, Albaina O, Moragues MD, Verdugo F, Ponton J (2010). The impact of oral contraceptives on women’s periodontal health and the subgingival occurrence of aggressive periodontopathogens and Candida species. J Periodontol.

[11] Baker JM, Al-Nakkash L, Herbst-Kralovetz MM (2017). Estrogen-gut microbiome axis: physiological and clinical implications. Maturitas.

[12] Braniste V, Leveque M, Buisson-Brenac C, Bueno L, Fioramonti J, Houdeau E (2009). Oestradiol decreases colonic permeability through oestrogen receptor beta-mediated up-regulation of occludin and junctional adhesion molecule-A in epithelial cells. J Physiol.

[13] Looijer-van Langen M, Hotte N, Dieleman LA, Albert E, Mulder C, Madsen KL (2011). Estrogen receptor-beta signaling modulates epithelial barrier function. Am J Physiol Gastrointest Liver Physiol.

[14] Garcia Rodriguez LA, Ruigomez A, Panes J (2006). Acute gastroenteritis is followed by an increased risk of inflammatory bowel disease. Gastroenterology.

[15] Segal AW (2016). Making sense of the cause of Crohn’s – a new look at an old disease. F1000Research.

[16] Ibrahim CB, Aroniadis OC, Brandt LJ (2010). On the role of ischemia in the pathogenesis of IBD: a review. Inflamm Bowel Dis.

[17] Vinogradova Y, Coupland C, Hippisley-Cox J (2015). Use of combined oral contraceptives and risk of venous thromboembolism: nested case-control studies using the QResearch and CPRD databases. BMJ.

[18] Benson T (2011). The history of the read codes: the inaugural James Read memorial lecture 2011. Informat Prim Care.

[19] Langley TE, Szatkowski LC, Wythe S, Lewis SA (2011). Can primary care data be used to monitor regional smoking prevalence? An analysis of The Health Improvement Network primary care data. BMC Public Health.

[20] Szatkowski L, Lewis S, McNeill A, Huang Y, Coleman T (2012). Can data from primary care medical records be used to monitor national smoking prevalence?. J Epidemiol Commun Health.

[21] Blak BT, Thompson M, Dattani H, Bourke A (2011). Generalisability of The Health Improvement Network (THIN) database: demographics, chronic disease prevalence and mortality rates. Informat Prim Care.

[22] Lewis JD, Brensinger C, Bilker WB, Strom BL (2002). Validity and completeness of the General Practice Research Database for studies of inflammatory bowel disease. Pharmacoepidemiol Drug Saf.

[23] Pasvol TJ, Horsfall L, Bloom S (2020). Incidence and prevalence of inflammatory bowel disease in UK primary care: a population-based cohort study. BMJ Open.

[24] Committee JF (2018). British National Formulary.

[25] Horsfall L, Walters K, Petersen I (2013). Identifying periods of acceptable computer usage in primary care research databases. Pharmacoepidemiol Drug Saf.

[26] Maguire A, Blak BT, Thompson M (2009). The importance of defining periods of complete mortality reporting for research using automated data from primary care. Pharmacoepidemiol Drug Saf.

[27] Lewis JD, Bilker WB, Weinstein RB, Strom BL (2005). The relationship between time since registration and measured incidence rates in the General Practice Research Database. Pharmacoepidemiol Drug Saf.

[28] Goldzieher JW, Brody SA (1990). Pharmacokinetics of ethinyl estradiol and mestranol. Am J Obstet Gynecol.

[29] Kirk JM, Wickramasuriya N, Shaw NJ (2016). Estradiol: micrograms or milligrams. Endocrinol Diabet Metabol Case Rep.

[30] VHA Pharmacy Benefits Management Strategic Healthcare Group, Program MAPaWVH (2003). Abbreviated Drug Class Review Combined estrogen and progestin products for hormone therapy.

[31] Jess T, Frisch M, Jørgensen KT, Pedersen BV, Nielsen NM (2012). Increased risk of inflammatory bowel disease in women with endometriosis: a nationwide Danish cohort study. Gut.

[32] Rispo A, Musto D, Imperatore N, Testa A, Rea M, Castiglione F (2016). Dramatic improvement of severe acne pustolosa after adalimumab in a patient with ulcerative colitis. Clin Case Rep.

[33] Murphy CL, Gibson D, Meyers LS (2009). Inflammatory bowel disease and acne. Am J Gastroenterol.

[34] Torres PJ, Siakowska M, Banaszewska B (2018). Gut microbial diversity in women with polycystic ovary syndrome correlates with hyperandrogenism. J Clin Endocrinol Metab.

[35] Higuchi LM, Khalili H, Chan AT, Richter JM, Bousvaros A, Fuchs CS (2012). A prospective study of cigarette smoking and the risk of inflammatory bowel disease in women. Am J Gastroenterol.

[36] Curtis KM, Jatlaoui TC, Tepper NK (2016). US selected practice recommendations for contraceptive use, 2016. MMWR Recommend Rep.

[37] Faculty of Sexual and Reproductive Healthcare (2019). UKMEC April 2016 Summary Sheet (Amended September 2019).

[38] Townsend P, Townsend P, Phillimore P, Beattie A (1988). Inequality and the North.

[39] French RS, Gibson L, Geary R, Glasier A, Wellings K (2020). Changes in the prevalence and profile of users of contraception in Britain 20002010: evidence from two National Surveys of Sexual Attitudes and Lifestyles. BMJ Sex Reproduct Health.

[40] Khalili H, Higuchi LM, Ananthakrishnan AN (2013). Oral contraceptives, reproductive factors and risk of inflammatory bowel disease. Gut.

[41] NHS Digital (2020). Sexual and Reproductive Health Services, England (Contraception) 2019/20.

[42] Molloy GJ, Graham H, McGuinness H (2012). Adherence to the oral contraceptive pill: a cross-sectional survey of modifiable behavioural determinants. BMC Public Health.

[43] Sanagapalli S, Ko Y, Kariyawasam V (2018). The association between new generation oral contraceptive pill and the development of inflammatory bowel diseases. Int Res.

[44] Khalili H, Granath F, Smedby KE (2016). Association between long-term oral contraceptive use and risk of Crohn’s disease complications in a nationwide study. Gastroenterology.

[45] Eriksson AL, Perry JRB, Coviello AD (2018). Genetic determinants of circulating estrogen levels and evidence of a causal effect of estradiol on bone density in men. J Clin Endocrinol Metabol.

[46] Liu JZ, van Sommeren S, Huang H (2015). Association analyses identify 38 susceptibility loci for inflammatory bowel disease and highlight shared genetic risk across populations. Nat Genet.

[47] Zhao J, Jiang CQ, Lam TH (2014). Genetically predicted 17β-estradiol and systemic inflammation in women: a separate-sample Mendelian randomisation analysis in the Guangzhou Biobank Cohort Study. J Epidemiol Commun Health.

