# Supplementary material for: Use of contraceptives and risk of inflammatory bowel disease: a nested case–control study
Source: Aliment Pharmacol Ther. Author manuscript; Available in PMC 2022 Jun 28. (PMC7612921; doi:10.1111/apt.16647)
Supplement: Supp info 1 [file EMS146182-supplement-Supp_info_1.docx]

**Supporting information – prescription code lists**

1. Post-menopausal hormone-replacement therapy

| Drug code | Generic name |
| --- | --- |
| 54611979 | Estradiol 500micrograms / Dydrogesterone 2.5mg tablets |
| 60462979 | Estradiol 50micrograms/24hours / Levonorgestrel 7micrograms/24hours transdermal patches |
| 60489979 | Estradiol 25micrograms/24hours transdermal patches |
| 60490979 | Estradiol 25micrograms/24hours transdermal patches |
| 78588978 | Estradiol 50micrograms/24hours transdermal patches |
| 82739998 | Estradiol 1mg gel sachets |
| 82740998 | Estradiol 1mg gel sachets |
| 82741998 | Estradiol 500microgram gel sachets |
| 82742998 | Estradiol 500microgram gel sachets |
| 83058998 | Generic hormonin tablets |
| 83429998 | Estradiol 100micrograms/24hours transdermal patches |
| 83430998 | Estradiol 75micrograms/24hours transdermal patches |
| 83431998 | Estradiol 25micrograms/24hours transdermal patches |
| 83432998 | Estradiol 50micrograms/24hours transdermal patches |
| 84780998 | Conjugated oestrogens 300microgram tablets |
| 84781998 | Conjugated oestrogens 300microgram tablets |
| 84862998 | Estradiol 0.06% gel (750microgram per actuation) |
| 85771979 | Ethinylestradiol 33.9micrograms/24hours / Norelgestromin 203micrograms/24hours transdermal patches |
| 85772979 | Ethinylestradiol 33.9micrograms/24hours / Norelgestromin 203micrograms/24hours transdermal patches |
| 85962998 | Estradiol 100micrograms/24hours transdermal patches |
| 85963998 | Estradiol 100micrograms/24hours transdermal patches |
| 85964998 | Estradiol 75micrograms/24hours transdermal patches |
| 85965998 | Estradiol 50micrograms/24hours transdermal patches |
| 85966998 | Estradiol 50micrograms/24hours transdermal patches |
| 85967998 | Estradiol 25micrograms/24hours transdermal patches |
| 85973998 | Estradiol 100micrograms/24hours transdermal patches |
| 85974998 | Estradiol 75micrograms/24hours transdermal patches |
| 85975998 | Estradiol 50micrograms/24hours transdermal patches |
| 85976998 | Estradiol 25micrograms/24hours transdermal patches |
| 86050998 | Generic Clinorette tablets |
| 86058998 | Estradiol 2mg tablets |
| 86546979 | Estradiol 1mg gel sachets |
| 86831998 | Estradiol 1mg / Drospirenone 2mg tablets |
| 86832998 | Estradiol 1mg / Drospirenone 2mg tablets |
| 87042998 | Estradiol 100micrograms/24hours transdermal patches |
| 87043998 | Estradiol 75micrograms/24hours transdermal patches |
| 87044998 | Estradiol 50micrograms/24hours transdermal patches |
| 87045998 | Estradiol 37.5micrograms/24hours transdermal patches |
| 87046998 | Estradiol 25micrograms/24hours transdermal patches |
| 87047998 | Estradiol 100micrograms/24hours transdermal patches |
| 87048998 | Estradiol 75micrograms/24hours transdermal patches |
| 87049998 | Estradiol 50micrograms/24hours transdermal patches |
| 87050998 | Estradiol 37.5micrograms/24hours transdermal patches |
| 87051998 | Estradiol 25micrograms/24hours transdermal patches |
| 87076979 | Generic Femoston 2/10mg tablets |
| 87082979 | Generic Evorel Sequi transdermal patches |
| 87549998 | Conjugated oestrogens 300microgram / Medroxyprogesterone 1.5mg modified-release tablets |
| 87550998 | Conjugated oestrogens 300microgram / Medroxyprogesterone 1.5mg modified-release tablets |
| 87759998 | Ethinylestradiol 2microgram tablets |
| 87898979 | Generic climagest 1mg tablets |
| 87901979 | Generic climagest 1mg tablets |
| 87953998 | Conjugated oestrogens equine with medroxyprogesterone acetate 625micrograms with 10mg tablets |
| 88207998 | Estradiol 1mg / Dydrogesterone 5mg tablets |
| 88320998 | Estradiol 2mg / Norethisterone acetate 1mg tablets |
| 88327998 | Estradiol 100micrograms/24hours transdermal patches |
| 88329998 | Estradiol 75micrograms/24hours transdermal patches |
| 88331997 | Estradiol 100micrograms/24hours transdermal patches |
| 88331998 | Estradiol 75micrograms/24hours transdermal patches |
| 88561998 | Phyto progesterone cream |
| 88634979 | Estradiol 1mg / Dydrogesterone 5mg tablets |
| 88635979 | Estradiol 1mg / Dydrogesterone 5mg tablets |
| 88638979 | Estradiol 1mg / Dydrogesterone 5mg tablets |
| 88826998 | Estradiol 80micrograms/24hours transdermal patches |
| 88828998 | Estradiol 40micrograms/24hours transdermal patches |
| 88835998 | Estradiol 50micrograms/24hours transdermal patches |
| 88887997 | Generic Evorel Sequi transdermal patches |
| 88887998 | Generic Evorel Sequi transdermal patches |
| 88889998 | Estradiol 50micrograms/24hours / Norethisterone 170micrograms/24hours transdermal patches |
| 88912998 | Estradiol valerate 2mg / norethisterone 700microgram tablets |
| 88915998 | Estradiol 1mg gel sachets |
| 88935998 | Estradiol hemihydrate 150mcg nasal spray |
| 88937998 | Estradiol 150micrograms/dose nasal spray |
| 89082996 | Estradiol 100micrograms/24hours transdermal patches |
| 89082997 | Estradiol 50micrograms/24hours transdermal patches |
| 89082998 | Estradiol 25micrograms/24hours transdermal patches |
| 89171979 | Conjugated estrogens & medroxyprogesterone 0.625mg+5mg tablets |
| 89173979 | Conjugated estrogens & medroxyprogesterone 0.625mg+5mg tablets |
| 89176979 | Conjugated estrogens & medroxyprogesterone 0.625mg+5mg tablets |
| 89209996 | Estradiol 75micrograms/24hours transdermal patches |
| 89209997 | Estradiol 50micrograms/24hours transdermal patches |
| 89209998 | Estradiol 25mcg transdermal patches |
| 89212998 | Estradiol and (estradiol with levonorgestrel) 80mcg/24hrs with (50mcg+20mcg/24hr) twice weekly patch |
| 89216998 | Generic nuvelle ts transdermal patches |
| 89253998 | Phyto progesterone cream |
| 89295998 | Ethinylestradiol 33.9micrograms/24hours / Norelgestromin 203micrograms/24hours transdermal patches |
| 89321998 | Generic FemSeven Sequi transdermal patches |
| 89359998 | Estradiol with dydrogesterone 1mg +10mg tablets |
| 89399998 | Estradiol 1mg / Norethisterone acetate 500microgram tablets |
| 89469998 | Generic Novofem tablets |
| 89500998 | Estradiol 50mcg/24hours vaginal ring |
| 89627998 | Estradiol 75micrograms/24hours transdermal patches |
| 89629998 | Estradiol 75micrograms/24hours transdermal patches |
| 89684979 | Conjugated oestrogens 625microgram tablets and norgestrel 150microgram tablets |
| 89685979 | Conjugated oestrogens 625microgram tablets and norgestrel 150microgram tablets |
| 89722979 | Estradiol 50micrograms/24hours / Norethisterone 170micrograms/24hours transdermal patches |
| 89723979 | Estradiol 50micrograms/24hours / Norethisterone 170micrograms/24hours transdermal patches |
| 89725979 | Estradiol 50micrograms/24hours / Norethisterone 170micrograms/24hours transdermal patches |
| 89803998 | Estradiol 1mg / dydrogesterone 5mg tablets |
| 89869998 | Phyto progesterone 1.5% cream |
| 89901998 | Progesterone 3% cream |
| 89907998 | Phyto progesterone 3% cream |
| 89953998 | Estradiol 2mg tablets |
| 90083998 | Generic Evorel Sequi transdermal patches |
| 90241996 | Estradiol 100micrograms/24hours transdermal patches |
| 90241997 | Estradiol 50micrograms/24hours transdermal patches |
| 90241998 | Estradiol 25micrograms/24hours transdermal patches |
| 90247996 | Estradiol 100micrograms/24hours transdermal patches |
| 90247997 | Estradiol 50micrograms/24hours transdermal patches |
| 90247998 | Estradiol 25micrograms/24hours transdermal patches |
| 90523998 | Estradiol valerate & norethisterone 2mg+0.7mg tablets |
| 90617998 | Estradiol valerate 2mg / Medroxyprogesterone 5mg tablets |
| 90618996 | Estradiol valerate 2mg / Medroxyprogesterone 5mg tablets |
| 90618997 | Estradiol valerate 1mg / Medroxyprogesterone 2.5mg tablets |
| 90618998 | Estradiol valerate 1mg / Medroxyprogesterone 5mg tablets |
| 90620998 | Estradiol 40micrograms/24hours transdermal patches and dydrogesterone 10mg tablets |
| 90645998 | Estradiol 50micrograms/24hours / Levonorgestrel 7micrograms/24hours transdermal patches |
| 90646998 | Estradiol 50micrograms/24hours / levonorgestrel 7micrograms/24hours transdermal patches |
| 90770998 | Piperazine oestrone sulphate 1.5mg with medroxyprogesterone 10mg tablet |
| 90771998 | Piperazine oestrone sulphate 1.5mg with medroxyprogesterone 10mg tablet |
| 90813998 | Estradiol acetate 1.25mg vaginal ring |
| 90819997 | Estradiol 100micrograms/24hours transdermal patches |
| 90819998 | Estradiol 50micrograms/24hours transdermal patches |
| 90834996 | Estradiol 25micrograms/24hours transdermal patches |
| 90834997 | Estradiol 100micrograms/24hours transdermal patches |
| 90834998 | Estradiol 75micrograms/24hours transdermal patches |
| 90835996 | Estradiol 100micrograms/24hours transdermal patches |
| 90835997 | Estradiol 50micrograms/24hours transdermal patches |
| 90835998 | Estradiol 50micrograms/24hours transdermal patches |
| 90873997 | Generic Elleste Duet 1mg tablets |
| 90873998 | Generic Elleste Duet 2mg tablets |
| 90875997 | Estradiol 2mg tablets |
| 90875998 | Estradiol 1mg tablets |
| 90894998 | Estradiol 50micrograms/24hours transdermal patches |
| 91052998 | Estradiol 80micrograms/24hours transdermal patches and dydrogesterone 10mg tablets |
| 91054998 | Estradiol 40micrograms/24hours transdermal patches |
| 91086998 | Estradiol valerate 2mg / Norethisterone 1mg tablets |
| 91090996 | Estradiol 75micrograms/24hours transdermal patches |
| 91090997 | Estradiol 50micrograms/24hours transdermal patches |
| 91090998 | Estradiol 37.5micrograms/24hours transdermal patches |
| 91096998 | Conjugat oestrogen equi and (conjugat oestrogen equi with medroxyprogesterone acetate 625 micrograms with (625 microgram |
| 91097998 | Generic premique cycle tablets |
| 91113998 | Conjugated oestrogens 625microgram / medroxyprogesterone 5mg tablets |
| 91114998 | Conjugated estrogens & medroxyprogesterone 0.625mg+5mg tablets |
| 91307997 | Estradiol 40micrograms/24hours transdermal patches and dydrogesterone 10mg tablets |
| 91307998 | Estradiol 80micrograms/24hours transdermal patches and dydrogesterone 10mg tablets |
| 91328998 | Generic adgyn combi tablets |
| 91350996 | Estradiol valerate 1mg / Medroxyprogesterone 2.5mg tablets |
| 91350997 | Estradiol valerate 1mg / Medroxyprogesterone 5mg tablets |
| 91350998 | Estradiol valerate 1mg / Medroxyprogesterone 2.5mg tablets |
| 91351998 | Generic Tridestra tablets |
| 91388996 | Estradiol and (estradiol with dydrogesterone) 2mg with (2mg with 20mg) tablets |
| 91388997 | Estradiol and (estradiol with dydrogesterone) 2mg with (2mg with 10 mg) tablets |
| 91388998 | Estradiol and (estradiol with dydrogesterone) 1mg with (1mg with 10mg) tablets |
| 91389996 | Generic femoston 2/20mg tablets |
| 91389997 | Generic Femoston 2/10mg tablets |
| 91389998 | Generic Femoston 1/10mg tablets |
| 91399997 | Estradiol 1mg gel sachets |
| 91399998 | Estradiol 0.06% gel (750microgram per actuation) |
| 91400998 | Estradiol 1.25g/dose gel |
| 91412996 | Estradiol 1mg / Norethisterone acetate 500microgram tablets |
| 91412997 | Estradiol with norethisterone acetate ( continuous combined) 2mg with 0.7mg tablets |
| 91412998 | Estradiol valerate 2mg / Norethisterone 1mg tablets |
| 91423998 | Estradiol 2mg / Norethisterone acetate 1mg tablets |
| 91457998 | Estradiol 80micrograms/24hours transdermal patches |
| 91469998 | Estradiol and (estradiol with levonorgestrel) 50mcg/24hrs with (50mcg+10mcg/24hrs) once weekly patch |
| 91479998 | Generic Tridestra tablets |
| 91546998 | Estradiol valerate 2mg / Norethisterone 1mg tablets |
| 91560998 | Progesterone 1.5% cream |
| 91620996 | Estradiol 75micrograms/24hours transdermal patches |
| 91620997 | Estradiol 50micrograms/24hours transdermal patches |
| 91620998 | Estradiol 37.5micrograms/24hours transdermal patches |
| 91680998 | Estradiol with norethisterone acetate 50mcg/24hours(4mg/unit) with 1mg patch with tablet |
| 91859998 | Estradiol valerate 1mg tablets |
| 91862998 | Estradiol 2mg / Norethisterone acetate 1mg tablets |
| 91864998 | Generic nuvelle tablets |
| 91865998 | Estradiol valerate 2mg tablets |
| 91871998 | Estradiol valerate with norgestrel 2mg+500micrograms tablets |
| 91878998 | Ethinylestradiol 33.9micrograms/24hours / Norelgestromin 203micrograms/24hours transdermal patches |
| 92065998 | Estradiol 100micrograms/24hours transdermal patches |
| 92171998 | Estradiol 1mg / Dydrogesterone 5mg tablets |
| 92221998 | Estradiol 25micrograms/24hr once weekly patch |
| 92251998 | Estradiol with (estradiol with norethisterone acetate) 1mg with (1mg with 1mg) tablets |
| 92366998 | Estradiol 100micrograms/24hours transdermal patches |
| 92371998 | Estradiol 50micrograms/24hours transdermal patches |
| 92440998 | Estradiol 2mg / Norethisterone acetate 1mg tablets |
| 92585998 | Estradiol with norethisterone 0mcg/24hours(3.2mg/unit) with 1mg patch with tablet |
| 92586998 | Estradiol 50micrograms/24hours transdermal patches and norethisterone 1mg tablets |
| 92962996 | Estradiol 40micrograms/24hours transdermal patches |
| 92962997 | Estradiol 80micrograms/24hours transdermal patches |
| 92962998 | Estradiol 100micrograms/24hours transdermal patches |
| 93073996 | Estradiol 75micrograms/24hours transdermal patches |
| 93073997 | Estradiol 25micrograms/24hours transdermal patches |
| 93073998 | Estradiol 50micrograms/24hours transdermal patches |
| 93164979 | Estradiol valerate & norethisterone 2mg+0.7mg tablets |
| 93165979 | Estradiol valerate & norethisterone 2mg+0.7mg tablets |
| 93169979 | Estradiol 2mg / Norethisterone acetate 1mg tablets |
| 93174979 | Estradiol 2mg / Norethisterone acetate 1mg tablets |
| 93189992 | Estradiol 50micrograms/24hours transdermal patches |
| 93191979 | Estradiol 0.06% gel (750microgram per actuation) |
| 93192979 | Estradiol 0.06% gel (750microgram per actuation) |
| 93193979 | Estradiol 0.06% gel (750microgram per actuation) |
| 93194979 | Estradiol 0.06% gel (750microgram per actuation) |
| 93195979 | Estradiol 0.06% gel (750microgram per actuation) |
| 93197979 | Tibolone 2.5mg tablets |
| 93201979 | Tibolone 2.5mg tablets |
| 93204979 | Tibolone 2.5mg tablets |
| 93211979 | Conjugated oestrogens 625microgram tablets |
| 93251979 | Estradiol 100micrograms/24hours transdermal patches |
| 93254979 | Estradiol 100micrograms/24hours transdermal patches |
| 93260979 | Estradiol 100micrograms/24hours transdermal patches |
| 93262979 | Estradiol 100micrograms/24hours transdermal patches |
| 93267979 | Estradiol 50micrograms/24hours transdermal patches |
| 93269979 | Estradiol 50micrograms/24hours transdermal patches |
| 93276979 | Estradiol 50micrograms/24hours transdermal patches |
| 93278979 | Estradiol 50micrograms/24hours transdermal patches |
| 93281979 | Estradiol 50micrograms/24hours transdermal patches |
| 93283979 | Estradiol 50micrograms/24hours transdermal patches |
| 93284979 | Estradiol 50micrograms/24hours transdermal patches |
| 93285979 | Estradiol 50micrograms/24hours transdermal patches |
| 93287979 | Estradiol 50micrograms/24hours transdermal patches |
| 93288979 | Estradiol 50micrograms/24hours transdermal patches |
| 93293979 | Estradiol 25micrograms/24hours transdermal patches |
| 93296979 | Estradiol 25micrograms/24hours transdermal patches |
| 93303979 | Estradiol 25micrograms/24hours transdermal patches |
| 93308979 | Estradiol 75micrograms/24hours transdermal patches |
| 93311979 | Estradiol 75micrograms/24hours transdermal patches |
| 93315998 | Tibolone 2.5mg tablets |
| 93319998 | Tibolone 2.5mg tablets |
| 93321979 | Estradiol valerate 2mg tablets |
| 93325979 | Estradiol valerate 2mg tablets |
| 93336992 | Ethinyloestradiol 5 mg tab |
| 93341979 | Estradiol valerate 1mg tablets |
| 93352979 | Estradiol 37.5micrograms/24hours transdermal patches |
| 93354979 | Estradiol 80micrograms/24hours transdermal patches |
| 93387992 | Ethinyloestradiol 2 mcg tab |
| 93461992 | Oestradiol 17b |
| 93578998 | Ethinylestradiol 1mg tablets |
| 93696997 | Estradiol valerate 2mg tablets |
| 93696998 | Estradiol valerate 1mg tablets |
| 93764992 | Conjugated oestrogens / norgestrel 1.25 mg tab |
| 94156992 | Ethinyloestradiol 15 mcg tab |
| 94161997 | Estradiol and (estradiol with norethisterone) and (estradiol) triphasic forte 4mg with (4mg with 1mg) with (1mg) tablets |
| 94161998 | Generic Trisequens tablets |
| 94162998 | Generic Cyclo-Progynova 2mg tablets |
| 94252992 | Conjugated oestrogens 625/norgestrel 500 mcg tab |
| 94309992 | Prempak 1.25mg mg tab |
| 94361992 | Tace 12 mg cap |
| 94458992 | Ethinyloestradiol 25 mcg tab |
| 94472997 | Conjugated oestrogens 1.25mg tablets and norgestrel 150microgram tablets |
| 94472998 | Conjugated oestrogens 625microgram tablets and norgestrel 150microgram tablets |
| 94516996 | Estradiol 100micrograms/24hours transdermal patches |
| 94516997 | Estradiol 50micrograms/24hours transdermal patches |
| 94516998 | Estradiol 25micrograms/24hours transdermal patches |
| 94517998 | Estradiol 50micrograms/24hours transdermal patches and norethisterone acetate 1mg tablets |
| 94518996 | Estradiol 25micrograms/24hours transdermal patches |
| 94518997 | Estradiol 100micrograms/24hours transdermal patches |
| 94518998 | Estradiol 25micrograms/24hours transdermal patches |
| 94519996 | Estradiol 75micrograms/24hours transdermal patches |
| 94519997 | Estradiol 25micrograms/24hours transdermal patches |
| 94519998 | Estradiol 50micrograms/24hours transdermal patches |
| 94737997 | Estradiol valerate 2mg tablets |
| 94737998 | Estradiol valerate 1mg tablets |
| 94918998 | Ethinylestradiol 33.9micrograms/24hours / Norelgestromin 203micrograms/24hours transdermal patches |
| 94971998 | Estropipate 1.5mg tablets |
| 94989992 | Ethinyloestradiol 30 mcg tab |
| 94990992 | Ethinyloestradiol 100 mcg tab |
| 95339998 | Quinestradol 250mcg tablets |
| 95351992 | Estradiol 50mg implant |
| 95363992 | Estriol 250mcg tablets |
| 95603998 | Generic nuvelle tablets |
| 95657997 | Estradiol valerate and (estradiol valerate with levonorgestrel) 1mg with (1mg with 250micrograms) tablets |
| 95657998 | Estradiol valerate and (estradiol valerate with levonorgestrel) 2mg with (2mg with 75micrograms) tablets |
| 95698997 | Norgestrel and conjugated oestrogens (equine) 150micrograms + 1.25mg tablet |
| 95698998 | Norgestrel and conjugated oestrogens (equine) 150micrograms + 625micrograms tablet |
| 96371992 | Oestradiol .01 mg tab |
| 96392997 | Dienestrol 5mg tablets |
| 96392998 | Dienestrol 1mg tablets |
| 96609996 | Conjugated oestrogens 2.5mg tablets |
| 96609997 | Conjugated oestrogens 1.25mg tablets |
| 96609998 | Conjugated oestrogens 625microgram tablets |
| 96744997 | Estriol 1mg tablets |
| 96744998 | Estriol 250micrograms tablets |
| 96745998 | Estradiol with estrone and estriol tablets |
| 96746992 | Premarin 1.25mg/norgestrel 0.15mg mg tab |
| 96746998 | Estradiol with estrone and estriol tablets |
| 96747996 | Estradiol 40micrograms/24hours transdermal patches |
| 96747997 | Estradiol 1mg tablets |
| 96747998 | Estradiol 2mg tablets |
| 96748997 | Estradio; 5m/ml injection |
| 96748998 | Estradiol 1mg/ml injection |
| 96892992 | Estradiol 50micrograms/24hours transdermal patches |
| 97387992 | Ethinyloestradiol 20 mcg pes |
| 97397992 | Ethinyloestradiol 5 mcg cap |
| 97404992 | Ethisterone 5 mg tab |
| 97457997 | Estradiol valerate 2mg tablets |
| 97457998 | Estradiol valerate 1mg tablets |
| 97458997 | Generic Cyclo-Progynova 2mg tablets |
| 97458998 | Generic cyclo-progynova 1mg tablets |
| 97482997 | Generic trisequens forte tablets |
| 97482998 | Generic Trisequens tablets |
| 97625997 | Estradiol valerate (2mg) with norethisterone (1 mg) tablets |
| 97625998 | Estradiol valerate and (estradiol valerate with norethisterone) 1mg with (1mg with 1mg) tablets |
| 97732998 | Generic estracombi tts transdermal patches |
| 97759996 | Estradiol 50micrograms/24hours / Norethisterone 170micrograms/24hours transdermal patches |
| 97759998 | Estradiol with (estradiol with norethisterone acetate) 50mcg/24 hr with (50mcg+250mcg/24 hr) twice weekly patch |
| 97762997 | Estradiol 1mg tablets |
| 97762998 | Estradiol 2mg tablets |
| 97765997 | Estradiol & norethisterone acetate 2mg+1mg tablets |
| 97765998 | Generic climagest 1mg tablets |
| 97826992 | Estradiol 1mg tablets |
| 97947992 | Premarin 0.625mg/norgestrel 0.15mg mg tab |
| 97993996 | Ethinylestradiol 50microgram tablets |
| 97993997 | Ethinylestradiol 20micrograms tablet |
| 97993998 | Ethinylestradiol 10microgram tablets |
| 98468989 | Estradiol 50mg implant |
| 98468990 | Estradiol 25mg implant |
| 98728998 | Ethinylestradiol with methyltestosterone 4.4micrograms + 3.6mg tablet |
| 98839998 | Conjugated oestrogens 1.25mg tablets and norgestrel 150microgram tablets |
| 98840998 | Conjugated oestrogens 1.25mg tablets and norgestrel 150microgram tablets |
| 98892998 | Conjugated oestrogens 625microgram tablets and norgestrel 150microgram tablets |
| 98897998 | Mestranol with norethisterone tablet |
| 98911996 | Estradiol 100mg implant |
| 98911997 | Estradiol 50mg implant |
| 98911998 | Estradiol 25mg implant |
| 99219998 | Conjugated oestrogens 625microgram tablets and norgestrel 150microgram tablets |
| 99220996 | Conjugated estrogens 2.5mg tablets |
| 99220997 | Conjugated oestrogens 1.25mg tablets |
| 99220998 | Conjugated oestrogens 625microgram tablets |
| 99295997 | Estriol 1mg tablets |
| 99295998 | Estriol 250mcg tablets |
| 99571998 | Estropipate 1.5mg tablets |
| 99602989 | Ethinylestradiol 50microgram tablets |
| 99602990 | Ethinylestradiol 10microgram tablets |

1. Drugs used to treat inflammatory bowel disease

| Drug code | Generic name |
| --- | --- |
| 30104978 | Adalimumab 40mg/0.4ml solution for injection pre-filled syringes |
| 30105978 | Adalimumab 40mg/0.4ml solution for injection pre-filled syringes |
| 30106978 | Adalimumab 40mg/0.4ml solution for injection pre-filled disposable devices |
| 53189979 | Ciclosporin 50mg capsules |
| 53190979 | Ciclosporin 100mg capsules |
| 53191979 | Ciclosporin 25mg capsules |
| 53913979 | Mesalazine 800mg gastro-resistant tablets |
| 54552979 | Mesalazine 400mg gastro-resistant tablets |
| 55164978 | Mesalazine 4g modified-release granules sachets sugar free |
| 55165978 | Mesalazine 4g modified-release granules sachets sugar free |
| 55193978 | Mercaptopurine 75mg tablets |
| 55575979 | Ciclosporin 50mg capsules |
| 55576979 | Ciclosporin 100mg capsules |
| 55577979 | Ciclosporin 25mg capsules |
| 55799978 | Mercaptopurine 30mg capsules |
| 56901978 | Ciclosporin 100mg capsules |
| 56902978 | Ciclosporin 25mg capsules |
| 58121979 | Mercaptopurine 25mg tablets |
| 58800979 | Mesalazine 1.2g gastro-resistant modified-release tablets |
| 60097979 | Prednisolone 20mg/application foam enema |
| 60124979 | Ciclosporin 50mg capsules |
| 60584979 | Mesalazine 3g gastro-resistant modified-release granules sachets sugar free |
| 60585979 | Mesalazine 3g gastro-resistant modified-release granules sachets sugar free |
| 61544979 | Adalimumab 40mg/0.8ml solution for injection vials |
| 61545979 | Adalimumab 40mg/0.8ml solution for injection vials |
| 62953979 | Sulfasalazine 250mg/5ml oral suspension sugar free |
| 64115979 | Methotrexate 25mg/3ml solution for injection pre-filled syringes |
| 64868979 | Mesalazine 2g modified-release granules sachets sugar free |
| 65098979 | Sulfasalazine 500mg/5ml oral suspension |
| 67089979 | Mesalazine 1.2g gastro-resistant modified-release tablets |
| 69316979 | Azathioprine 125mg/5ml oral suspension |
| 70274978 | Azathioprine 50mg tablets |
| 70879979 | Adalimumab 40mg prefilled pen |
| 70880979 | Adalimumab 40mg/0.8ml solution for injection pre-filled disposable devices |
| 72688978 | Ciclosporin 50mg capsules |
| 73065978 | Methotrexate 7.5mg/0.15ml solution for injection pre-filled disposable devices |
| 73066978 | Methotrexate 7.5mg/0.15ml solution for injection pre-filled disposable devices |
| 73067978 | Methotrexate 30mg/0.6ml solution for injection pre-filled disposable devices |
| 73068978 | Methotrexate 30mg/0.6ml solution for injection pre-filled disposable devices |
| 73069978 | Methotrexate 27.5mg/0.55ml solution for injection pre-filled disposable devices |
| 73071978 | Methotrexate 25mg/0.5ml solution for injection pre-filled disposable devices |
| 73072978 | Methotrexate 25mg/0.5ml solution for injection pre-filled disposable devices |
| 73073978 | Methotrexate 22.5mg/0.45ml solution for injection pre-filled disposable devices |
| 73074978 | Methotrexate 22.5mg/0.45ml solution for injection pre-filled disposable devices |
| 73075978 | Methotrexate 20mg/0.4ml solution for injection pre-filled disposable devices |
| 73076978 | Methotrexate 20mg/0.4ml solution for injection pre-filled disposable devices |
| 73077978 | Methotrexate 17.5mg/0.35ml solution for injection pre-filled disposable devices |
| 73078978 | Methotrexate 17.5mg/0.35ml solution for injection pre-filled disposable devices |
| 73079978 | Methotrexate 15mg/0.3ml solution for injection pre-filled disposable devices |
| 73080978 | Methotrexate 15mg/0.3ml solution for injection pre-filled disposable devices |
| 73081978 | Methotrexate 12.5mg/0.25ml solution for injection pre-filled disposable devices |
| 73082978 | Methotrexate 12.5mg/0.25ml solution for injection pre-filled disposable devices |
| 73083978 | Methotrexate 10mg/0.2ml solution for injection pre-filled disposable devices |
| 73084978 | Methotrexate 10mg/0.2ml solution for injection pre-filled disposable devices |
| 76424978 | Mesalazine 1g modified-release tablets |
| 76878978 | Methotrexate 2.5mg tablets |
| 78442979 | Methotrexate 50mg/2ml solution for injection vials |
| 78447979 | Methotrexate 25mg/1ml solution for injection pre-filled syringes |
| 78449979 | Methotrexate 20mg/0.8ml solution for injection pre-filled syringes |
| 78452979 | Methotrexate 12.5mg/0.5ml solution for injection pre-filled syringes |
| 79522979 | Mercaptopurine 25mg/5ml oral suspension |
| 79524979 | Mercaptopurine 20mg/ml oral suspension |
| 79739978 | Sulfasalazine 500mg gastro-resistant tablets |
| 79867978 | Mesalazine 400mg gastro-resistant tablets |
| 80925979 | Azathioprine 25mg/5ml oral suspension |
| 80927979 | Azathioprine 25mg/5ml oral solution |
| 80928998 | Mesalazine 3g gastro-resistant modified-release granules sachets sugar free |
| 80929979 | Azathioprine 20mg/5ml oral suspension |
| 80929998 | Mesalazine 3g gastro-resistant modified-release granules sachets sugar free |
| 80961979 | Azathioprine 10mg/5ml oral suspension |
| 81193998 | Mesalazine 1g modified-release tablets |
| 81194998 | Mesalazine 1g modified-release tablets |
| 81282998 | Ciclosporin 100mg capsules |
| 81283998 | Ciclosporin 50mg capsules |
| 81284998 | Ciclosporin 25mg capsules |
| 81490998 | Methotrexate 27.5mg/0.55ml prefilled syringes |
| 81491998 | Methotrexate 22.5mg/0.45ml prefilled syringes |
| 81492998 | Methotrexate 17.5mg/0.35ml prefilled syringes |
| 81493998 | Methotrexate 12.5mg/0.25ml prefilled syringes |
| 81494998 | Methotrexate 27.5mg/0.55ml solution for injection pre-filled syringes |
| 81495998 | Methotrexate 22.5mg/0.45ml solution for injection pre-filled syringes |
| 81496998 | Methotrexate 17.5mg/0.35ml solution for injection pre-filled syringes |
| 81498998 | Methotrexate 12.5mg/0.25ml solution for injection pre-filled syringes |
| 81638998 | Methotrexate 30mg/1.5ml solution for injection pre-filled syringes |
| 81640998 | Methotrexate 25mg/1.25ml solution for injection pre-filled syringes |
| 81642998 | Methotrexate 20mg/1ml solution for injection pre-filled syringes |
| 81643998 | Methotrexate 7.5mg/0.75ml prefilled syringes |
| 81683998 | Mesalazine 1g suppositories |
| 81689998 | Mesalazine 500mg gastro-resistant tablets |
| 81690998 | Mesalazine 500mg gastro-resistant tablets |
| 81771998 | Ciclosporin 25mg capsules |
| 81772998 | Mesalazine 800mg gastro-resistant tablets |
| 81773998 | Ciclosporin 100mg capsules |
| 81774998 | Ciclosporin 50mg capsules |
| 81815998 | Methotrexate 30mg/0.6ml prefilled syringes |
| 81816998 | Methotrexate 30mg/0.6ml solution for injection pre-filled syringes |
| 81868998 | Mesalazine 400mg gastro-resistant tablets |
| 82203998 | Azathioprine 50mg tablets |
| 82204998 | Azathioprine 25mg tablets |
| 82480998 | Ciclosporin 100mg capsules |
| 82481998 | Ciclosporin 50mg capsules |
| 82482998 | Ciclosporin 25mg capsules |
| 82840998 | Methotrexate 25mg/0.5ml prefilled syringes |
| 82841998 | Methotrexate 20mg/0.4ml prefilled syringes |
| 82842998 | Methotrexate 15mg/0.3ml prefilled syringes |
| 82843998 | Methotrexate 10mg/0.2ml prefilled syringes |
| 82844998 | Methotrexate 7.5mg/0.15ml prefilled syringes |
| 82845998 | Methotrexate 25mg/0.5ml solution for injection pre-filled syringes |
| 82846998 | Methotrexate 20mg/0.4ml solution for injection pre-filled syringes |
| 82847998 | Methotrexate 15mg/0.3ml solution for injection pre-filled syringes |
| 82848998 | Methotrexate 10mg/0.2ml solution for injection pre-filled syringes |
| 82849998 | Methotrexate 7.5mg/0.15ml solution for injection pre-filled syringes |
| 83228978 | Ciclosporin 50mg capsules |
| 83229978 | Ciclosporin 100mg capsules |
| 83485998 | Azathioprine 10mg capsules |
| 83503998 | Mesalazine 1.5g gastro-resistant modified-release granules sachets sugar free |
| 83504998 | Mesalazine 1.5g gastro-resistant modified-release granules sachets sugar free |
| 83559978 | Mercaptopurine 50mg tablets |
| 83743998 | Mesalazine 2g modified-release granules sachets sugar free |
| 83769998 | Sulfasalazine 250mg/5ml oral suspension sugar free |
| 83987998 | Mesalazine 2g modified-release granules sachets sugar free |
| 84059998 | Mesalazine 1g modified-release granules sachets sugar free |
| 84209998 | Mesalazine 800mg gastro-resistant tablets |
| 84290998 | Mesalazine 1.2g gastro-resistant modified-release tablets |
| 84291998 | Mesalazine 1.2g gastro-resistant modified-release tablets |
| 84438998 | Methotrexate 2mg/ml oral solution sugar free |
| 84439998 | Methotrexate 10mg/5ml oral suspension |
| 84636998 | Budesonide 2mg foam enema |
| 84637998 | Budesonide 2mg foam enema |
| 84741998 | Prednisolone 40mg/100ml enema |
| 84920998 | Ciclosporin 250mg/5ml solution for infusion ampoules |
| 84921998 | Ciclosporin 50mg/1ml solution for infusion ampoules |
| 84927998 | Ciclosporin 250mg/5ml solution for infusion ampoules |
| 84928998 | Ciclosporin 50mg/1ml solution for infusion ampoules |
| 85097998 | Methotrexate 1g/40ml solution for injection vials |
| 85100998 | Methotrexate 7.5mg/5ml oral suspension |
| 85188978 | Methotrexate 2.5mg tablets |
| 85252998 | Azathioprine 50mg/5ml oral suspension |
| 85556998 | Mercaptopurine oral solution |
| 85560998 | Mesalazine 800mg gastro-resistant tablets |
| 85639998 | Methotrexate 7.5mg/0.75ml prefilled syringes |
| 85640998 | Methotrexate 10mg/ml prefilled syringes |
| 85641998 | Methotrexate 15mg/1.5ml prefilled syringes |
| 85642998 | Methotrexate 20mg/2ml prefilled syringes |
| 85643998 | Methotrexate 25mg/2.5ml prefilled syringes |
| 85644998 | Methotrexate 7.5mg/0.75ml solution for injection pre-filled syringes |
| 85645998 | Methotrexate 10mg/1ml solution for injection pre-filled syringes |
| 85646998 | Methotrexate 15mg/1.5ml solution for injection pre-filled syringes |
| 85648998 | Methotrexate 20mg/2ml solution for injection pre-filled syringes |
| 85650998 | Methotrexate 25mg/2.5ml solution for injection pre-filled syringes |
| 85737998 | Methotrexate 5g/50ml solution for infusion vials |
| 85738998 | Methotrexate 1g/10ml solution for injection vials |
| 85776998 | Methotrexate oral solution |
| 85777998 | Methotrexate 12.5mg/5ml oral suspension |
| 86327998 | Methotrexate 2.5mg/5ml oral suspension |
| 86339998 | Methotrexate 5mg/0.2ml solution for injection pre-filled syringes |
| 86342998 | Methotrexate 22.5mg/0.9ml solution for injection pre-filled syringes |
| 86343998 | Methotrexate 15mg/0.6ml solution for injection pre-filled syringes |
| 86344998 | Methotrexate 12.5mg/0.5ml solution for injection pre-filled syringes |
| 86345998 | Methotrexate 10mg/0.4ml solution for injection pre-filled syringes |
| 86427998 | Methotrexate 17.5mg/0.7ml solution for injection pre-filled syringes |
| 86434998 | Methotrexate 20mg/0.8ml solution for injection pre-filled syringes |
| 86435998 | Methotrexate 25mg/1ml solution for injection pre-filled syringes |
| 86436998 | Methotrexate 500mg/20ml solution for injection vials |
| 86437998 | Methotrexate 50mg/2ml solution for injection vials |
| 86438998 | Methotrexate 7.5mg/0.3ml solution for injection pre-filled syringes |
| 86439998 | Methotrexate 30mg/1.2ml solution for injection pre-filled syringes |
| 86440998 | Methotrexate 27.5mg/1.1ml solution for injection pre-filled syringes |
| 86470998 | Azathioprine 250mg/5ml oral solution |
| 86519998 | Azathioprine 50mg/5ml oral solution |
| 87675998 | Mercaptopurine 10mg capsules |
| 87757998 | Mercaptopurine 10mg tablets |
| 87761998 | Mesalazine 400mg gastro-resistant tablets |
| 87859998 | Adalimumab 40mg prefilled syringes |
| 87862998 | Adalimumab 40mg injection |
| 87909998 | Mesalazine 1g gastro-resistant modified-release granules sachets sugar free |
| 87910998 | Mesalazine 500mg gastro-resistant modified-release granules sachets sugar free |
| 87911998 | Mesalazine 500mg gastro-resistant modified-release granules sachets sugar free |
| 88489998 | Balsalazide 750mg capsules |
| 88492998 | Balsalazide 750mg capsules |
| 88517998 | Mesalazine 400mg gastro-resistant tablets |
| 88519998 | Sulfasalazine 500mg gastro-resistant tablets |
| 89238998 | Budesonide 2mg/100ml enema |
| 89239998 | Budesonide 2mg/100ml enema |
| 89244979 | Adalimumab 40mg prefilled syringes |
| 89245979 | Adalimumab 40mg/0.8ml solution for injection pre-filled syringes |
| 89460998 | Hydrocortisone 1% / Pramocaine 1% foam enema |
| 89598997 | Sulfasalazine 3g/100ml retention enema |
| 89598998 | Sulfasalazine 500mg suppositories |
| 89604997 | Sulfasalazine 3g/100ml retention enema |
| 89604998 | Sulfasalazine 500mg suppositories |
| 89610997 | Sulfasalazine 250mg/5ml oral suspension |
| 89610998 | Sulfasalazine 500mg tablets |
| 89616997 | Sulfasalazine 250mg/5ml oral suspension sugar free |
| 89616998 | Sulfasalazine 500mg gastro-resistant tablets |
| 89651998 | Infliximab 100mg powder for solution for infusion vials |
| 89992997 | Mesalazine 2g/59ml enema |
| 89992998 | Mesalazine 500mg suppositories |
| 90310979 | Prednisolone 20mg/application foam enema |
| 91215998 | Azathioprine 50mg tablets |
| 91309998 | Azathioprine capsules |
| 91373997 | Ciclosporin 10mg capsules |
| 91373998 | Ciclosporin 100mg/ml oral solution sugar free |
| 91601979 | Sulfasalazine 500mg gastro-resistant tablets |
| 92244990 | Ciclosporin 100mg capsules |
| 92245990 | Ciclosporin 50mg capsules |
| 92246990 | Ciclosporin 25mg capsules |
| 92346998 | Mesalazine 1g/application foam enema |
| 92347998 | Mesalazine 400mg gastro-resistant tablets |
| 92400998 | Olsalazine 500mg tablets |
| 92401998 | Olsalazine 250mg capsules |
| 92488997 | Methotrexate 2.5mg tablets |
| 92488998 | Methotrexate 5g/200ml solution for infusion vials |
| 92511998 | Infliximab 100mg powder for solution for infusion vials |
| 92544979 | Ciclosporin 50mg capsules |
| 92552979 | Ciclosporin 100mg capsules |
| 92555979 | Ciclosporin 100mg capsules |
| 92558979 | Ciclosporin 25mg capsules |
| 92559979 | Ciclosporin 25mg capsules |
| 92566979 | Azathioprine 25mg tablets |
| 92570979 | Azathioprine 25mg tablets |
| 92571979 | Azathioprine 25mg tablets |
| 92579979 | Azathioprine 50mg tablets |
| 92639979 | Methotrexate 5g/200ml solution for infusion vials |
| 92650979 | Methotrexate 200mg/8ml solution for injection vials |
| 92655979 | Methotrexate 50mg/2ml solution for injection vials |
| 92764997 | Mesalazine 1g modified-release granules sachets sugar free |
| 92764998 | Mesalazine 500mg modified-release tablets |
| 92930998 | Azathioprine 50mg tablets |
| 92989996 | Ciclosporin 100mg capsules |
| 92989997 | Ciclosporin 50mg capsules |
| 92989998 | Ciclosporin 25mg capsules |
| 93074990 | Methotrexate 2.5mg tablets |
| 93623996 | Mesalazine 250mg suppositories |
| 93623997 | Mesalazine 1g/application foam enema |
| 93623998 | Mesalazine 1g/100ml enema |
| 93624996 | Mesalazine 1g suppositories |
| 93624997 | Mesalazine 250mg modified release tablets |
| 93624998 | Mesalazine 1g/100ml enema |
| 93728992 | Mesalazine 500mg modified-release tablets |
| 94042990 | Sulfasalazine 500mg gastro-resistant tablets |
| 94078992 | Azathioprine 100 mg tab |
| 94153998 | Hydrocortisone 10% foam aerosol enema |
| 94155997 | Hydrocortisone retention enema |
| 94155998 | Hydrocortisone 10% foam aerosol enema |
| 94308990 | Azathioprine 50mg tablets |
| 94417992 | Azathioprine 50 mg sus |
| 94437997 | Olsalazine 500mg tablets |
| 94437998 | Olsalazine 250mg capsules |
| 94438997 | Olsalazine 500mg tablets |
| 94438998 | Olsalazine 250mg capsules |
| 94451998 | Prednisolone 20mg/application foam enema |
| 94452998 | Prednisolone 20mg/application foam enema |
| 94564992 | Mesalazine 500mg modified-release tablets |
| 94593997 | Ciclosporin 10mg capsules |
| 94593998 | Ciclosporin 50mg capsules |
| 94600990 | Methotrexate 20mg/0.8ml solution for injection pre-filled syringes |
| 94690992 | Azathioprine 125 mg tab |
| 94691992 | Azathioprine 10 mg tab |
| 94697998 | Azathioprine 50mg tablets |
| 94818998 | Sulfasalazine 250mg/5ml oral suspension |
| 95041990 | Mesalazine 400mg gastro-resistant tablets |
| 95153992 | Azathioprine 10mg tablets |
| 95252990 | Azathioprine 50mg tablets |
| 95255998 | Sulfasalazine 500mg gastro-resistant tablets |
| 95256996 | Sulfasalazine 3g/100ml enema |
| 95256997 | Sulfasalazine 500mg suppositories |
| 95256998 | Sulfasalazine 500mg gastro-resistant tablets |
| 95589998 | Sulfasalazine 250mg/5ml oral suspension |
| 95725990 | Azathioprine 50mg tablets |
| 95866998 | Methotrexate 25mg/ml injection |
| 95867996 | Methotrexate 5mg/2ml solution for injection vials |
| 95867997 | Methotrexate 10mg tablets |
| 95867998 | Methotrexate 2.5mg tablets |
| 95868996 | Methotrexate 50mg/3ml Injection |
| 95868997 | Methotrexate 5g/50ml solution for infusion vials |
| 95868998 | Methotrexate 200mg/8ml solution for injection vials |
| 95869997 | Methotrexate 10mg tablets |
| 95869998 | Methotrexate 2.5mg tablets |
| 95888997 | Mesalazine 250mg gastro-resistant tablets |
| 95888998 | Mesalazine 400mg gastro-resistant tablets |
| 95890998 | Mercaptopurine 50mg tablets |
| 95891998 | Mercaptopurine 50mg tablets |
| 96177998 | Hydrocortisone 1% / pramocaine 1% foam enema |
| 96199990 | Azathioprine 50mg tablets |
| 96279990 | Methotrexate 2.5mg tablets |
| 96580998 | Ciclosporin 50mg/1ml solution for infusion ampoules |
| 96581996 | Ciclosporin 100mg capsules |
| 96581997 | Ciclosporin 25mg capsules |
| 96581998 | Ciclosporin 100mg/ml oral solution sugar free |
| 96608996 | Mesalazine 2g/59ml enema |
| 96608997 | Mesalazine 1g suppositories |
| 96608998 | Mesalazine 500mg suppositories |
| 96659996 | Mesalazine 1g/application foam enema |
| 96659997 | Mesalazine 500mg suppositories |
| 96659998 | Mesalazine 250mg suppositories |
| 96752989 | Methotrexate 10mg tablets |
| 96752990 | Methotrexate 2.5mg tablets |
| 96803990 | Sulfasalazine 500mg gastro-resistant tablets |
| 96820988 | Methotrexate 5mg/2ml solution for injection vials |
| 96883990 | Mesalazine 400mg gastro-resistant tablets |
| 96916992 | Mesalazine 500mg modified-release tablets |
| 96922989 | Azathioprine 50mg tablets |
| 96922990 | Azathioprine 25mg tablets |
| 96932998 | Azathioprine 50mg powder for solution for injection vials |
| 96933998 | Azathioprine 50mg tablets |
| 96934997 | Azathioprine 50mg powder for solution for injection vials |
| 96934998 | Azathioprine 25mg tablets |
| 97036997 | Azathioprine 10mg tablets |
| 97036998 | Azathioprine 50mg tablets |
| 97280998 | Sulfasalazine 500mg gastro-resistant tablets |
| 97281996 | Sulfasalazine 3g/100ml retention enema |
| 97281997 | Sulfasalazine 500mg suppositories |
| 97281998 | Sulfasalazine 500mg tablets |
| 97287998 | Hydrocortisone acetate & pramocaine foam |
| 97362990 | Sulfasalazine 500mg gastro-resistant tablets |
| 97363990 | Sulfasalazine 500mg gastro-resistant tablets |
| 97381998 | Mesalazine 400mg gastro-resistant tablets |
| 97719989 | Sulfasalazine 500mg gastro-resistant tablets |
| 97719990 | Sulfasalazine 500mg gastro-resistant tablets |
| 97764998 | Mesalazine 250mg gastro-resistant tablets |
| 97785990 | Azathioprine 50mg tablets |
| 98001992 | Mesalazine 250mg gastro-resistant tablets |
| 98013988 | Methotrexate 5g/50ml solution for infusion vials |
| 98211990 | Azathioprine 50mg tablets |
| 98238996 | Ciclosporin 25mg capsules |
| 98238998 | Ciclosporin 100mg/ml oral solution sugar free |
| 98365990 | Sulfasalazine 500mg gastro-resistant tablets |
| 98639990 | Azathioprine 50mg tablets |
| 98640990 | Azathioprine 50mg tablets |
| 98950997 | Ciclosporin 50mg capsules |
| 98950998 | Ciclosporin 100mg capsules |
| 98958988 | Methotrexate 2.5mg tablets |
| 98959989 | Methotrexate 10mg tablets |
| 98959990 | Methotrexate 2.5mg tablets |
| 99374990 | Sulfasalazine 500mg gastro-resistant tablets |
| 99394979 | Hydrocortisone 10% foam aerosol enema |
| 99472979 | Sulfasalazine 500mg gastro-resistant tablets |
| 99486979 | Mesalazine 400mg gastro-resistant tablets |
| 99487979 | Mesalazine 400mg gastro-resistant tablets |
| 99488979 | Mesalazine 400mg gastro-resistant tablets |
| 99490979 | Mesalazine 1g modified-release granules sachets sugar free |
| 99492979 | Mesalazine 1g suppositories |
| 99494979 | Mesalazine 1g suppositories |
| 99495979 | Mesalazine 500mg modified-release tablets |
| 99498979 | Mesalazine 500mg modified-release tablets |
| 99583996 | Mesalazine 1g modified-release granules sachets sugar free |
| 99583997 | Mesalazine 500mg modified-release tablets |
| 99583998 | Mesalazine 250mg modified-release tablet |
| 99797989 | Azathioprine 25mg tablets |
| 99797990 | Azathioprine 50mg tablets |
| 99798990 | Azathioprine 50mg tablets |
| 99799990 | Azathioprine 50mg tablets |
| 99956998 | Methotrexate 5mg/2ml solution for injection vials |
| 39760978 | Ustekinumab 90mg/1ml solution for injection pre-filled syringes |
| 62544979 | Ustekinumab 45mg/0.5ml solution for injection pre-filled syringes |
| 62545979 | Ustekinumab 45mg/0.5ml solution for injection pre-filled syringes |
| 64015979 | Ustekinumab 45mg/0.5ml solution for injection vials |
| 72853978 | Vedolizumab 300mg powder for solution for infusion vials |
| 83106998 | Ustekinumab 45mg/0.5ml solution for injection vials |
| 83108998 | Ustekinumab 45mg/0.5ml solution for injection vials |

1. Second generation combined oral contraceptive pills

| Drug code | Generic name |
| --- | --- |
| 38631978 | Ethinylestradiol 35microgram / Norgestimate 250microgram tablets |
| 42618978 | Ethinylestradiol 35microgram / Norgestimate 250microgram tablets |
| 58067979 | Ethinylestradiol 30microgram / Levonorgestrel 150microgram tablets |
| 61424979 | Ethinylestradiol 35microgram / Norgestimate 250microgram tablets |
| 72983978 | Ethinylestradiol 30microgram / Levonorgestrel 150microgram tablets |
| 72984978 | Ethinylestradiol 30microgram / Levonorgestrel 150microgram tablets |
| 81388998 | Ethinylestradiol 30microgram / Levonorgestrel 150microgram tablets |
| 81713998 | Ethinylestradiol 30microgram / Levonorgestrel 150microgram tablets |
| 82039998 | Generic Logynon tablets |
| 82040998 | Ethinylestradiol 30microgram / Levonorgestrel 150microgram tablets |
| 82343998 | Ethinylestradiol 30microgram / Levonorgestrel 150microgram tablets |
| 83562978 | Ethinylestradiol 35microgram / Norgestimate 250microgram tablets |
| 89080998 | Generic Microgynon 30 ED tablets |
| 89213998 | Ethinylestradiol 30microgram / Levonorgestrel 150microgram tablets |
| 90566998 | Ethinylestradiol with norethisterone - biphasic 7 x 35mcg+500mcg; 14 x 35mcg+1mg tablet |
| 90641998 | Generic Logynon ED tablets |
| 90644998 | Generic Logynon tablets |
| 90647998 | Levonorgestrel 250microgram / ethinylestradiol 50microgram tablets |
| 90650998 | Levonorgestrel 250microgram / ethinylestradiol 30microgram tablets |
| 90654998 | Ethinylestradiol 30microgram / Levonorgestrel 150microgram tablets |
| 90658998 | Ethinylestradiol & levonorgestrel 50mcg+250mcg tablets |
| 90703997 | Ethinylestradiol with norethisterone - triphasic 7 x 35+500mcg; 7 x 35+750mcg; 7 x 35mcg+1mg tablet |
| 90972998 | Ethinylestradiol 35microgram / Norgestimate 250microgram tablets |
| 92682998 | Mestranol 50microgram / Norethisterone 1mg tablets |
| 92860979 | Ethinylestradiol & levonorgestrel 50mcg+250mcg tablets |
| 92862979 | Ethinylestradiol 30microgram / Levonorgestrel 150microgram tablets |
| 94158996 | Ethinylestradiol 30microgram / Norethisterone acetate 1.5mg tablets |
| 94158997 | Ethinylestradiol 20microgram / Norethisterone acetate 1mg tablets |
| 94997992 | Ethinylestradiol 30microgram / Levonorgestrel 150microgram tablets |
| 95885998 | Mestranol 50microgram / Norethisterone 1mg tablets |
| 97456998 | Ethinylestradiol & levonorgestrel 50mcg+250mcg tablets |
| 97462998 | Generic Logynon ED tablets |
| 97464998 | Ethinylestradiol 30microgram / Levonorgestrel 150microgram tablets |
| 97466998 | Ethinylestradiol & levonorgestrel 30mcg+250mcg tablets |
| 97563998 | Generic Synphase tablets |
| 98085997 | Ethinylestradiol 35microgram / Norethisterone 1mg tablets |
| 98085998 | Ethinylestradiol 35microgram / Norethisterone 500microgram tablets |
| 98181997 | Ethinylestradiol with norethisterone - triphasic and placebo 7 x 35+500mcg; 7 x 35+750mcg; 7 x 35mcg+1mg tablet |
| 98181998 | Generic trinovum tablets |
| 98183998 | Ethinylestradiol 35microgram / Norethisterone 500microgram tablets |
| 98185998 | Mestranol & norethisterone 50mcg+1mg tablets |
| 98187998 | Ethinylestradiol & norethisterone 35mcg+1mg tablets |
| 98189998 | Generic binovum tablets |
| 98191998 | Mestranol 50microgram / Norethisterone 1mg tablets |
| 98193998 | Ethinylestradiol 35microgram / Norethisterone 500microgram tablets |
| 98195998 | Ethinylestradiol 35microgram / Norethisterone 1mg tablets |
| 98197998 | Generic Logynon tablets |
| 98199998 | Ethinylestradiol 30microgram / Levonorgestrel 150microgram tablets |
| 98201998 | Ethinylestradiol & levonorgestrel 30mcg+250mcg tablets |
| 98203998 | Ethinylestradiol & levonorgestrel 50mcg+250mcg tablets |
| 98205998 | Generic Logynon tablets |
| 98207998 | Ethinylestradiol 30microgram / Norethisterone acetate 1.5mg tablets |
| 98209998 | Ethinylestradiol 20microgram / Norethisterone acetate 1mg tablets |
| 99036998 | Ethinylestradiol 35microgram / Norgestimate 250microgram tablets |
| 99047998 | Ethinylestradiol 35microgram / Norgestimate 250microgram tablets |
| 89341998 | Ethinylestradiol with levonorgestrel 30micrograms + 50micrograms tablet |
| 90703998 | Ethinylestradiol with norethisterone - triphasic 7x35+500mcg; 9x35mcg+1mg; 5x35+500mcg tablet |
| 93781998 | Ethinylestradiol with levonorgestrel tablet |
| 94995992 | Ethinyloestradiol/norethisterone 35 mcg tab |
| 95002992 | Ethinylestradiol & levonorgestrel 50mcg+250mcg tablets |

1. Third generation combined oral contraceptive pills

| Drug code | Generic name |
| --- | --- |
| 39702978 | Ethinylestradiol 30microgram / Desogestrel 150microgram tablets |
| 47222978 | Ethinylestradiol 20microgram / Desogestrel 150microgram tablets |
| 52480979 | Ethinylestradiol 30microgram / Desogestrel 150microgram tablets |
| 52481979 | Ethinylestradiol 20microgram / Desogestrel 150microgram tablets |
| 53192979 | Ethinylestradiol 30microgram / Desogestrel 150microgram tablets |
| 59313978 | Ethinylestradiol 30microgram / Desogestrel 150microgram tablets |
| 72985978 | Ethinylestradiol 30microgram / Desogestrel 150microgram tablets |
| 72986978 | Ethinylestradiol 30microgram / Desogestrel 150microgram tablets |
| 82024998 | Ethinylestradiol 20microgram / Gestodene 75microgram tablets |
| 82029998 | Ethinylestradiol 30microgram / Gestodene 75microgram tablets |
| 82032998 | Ethinylestradiol 20microgram / Desogestrel 150microgram tablets |
| 82041998 | Ethinylestradiol 30microgram / Desogestrel 150microgram tablets |
| 84491998 | Ethinylestradiol 20microgram / Gestodene 75microgram tablets |
| 84492998 | Ethinylestradiol 30microgram / Gestodene 75microgram tablets |
| 90747998 | Ethinylestradiol 30microgram / Desogestrel 150microgram tablets |
| 90750998 | Ethinylestradiol 20microgram / Desogestrel 150microgram tablets |
| 90760998 | Generic Femodene ED tablets |
| 90969997 | Ethinylestradiol 20microgram / Gestodene 75microgram tablets |
| 90969998 | Ethinylestradiol 30microgram / Gestodene 75microgram tablets |
| 92485998 | Ethinylestradiol 20microgram / Gestodene 75microgram tablets |
| 92863979 | Ethinylestradiol 30microgram / Desogestrel 150microgram tablets |
| 93263998 | Generic Femodene ED tablets |
| 94398997 | Ethinylestradiol 20microgram / Gestodene 75microgram tablets |
| 94398998 | Ethinylestradiol 30microgram / Gestodene 75microgram tablets |
| 94745998 | Ethinylestradiol 20microgram / Desogestrel 150microgram tablets |
| 94773998 | Ethinylestradiol 30microgram / Gestodene 75microgram tablets |
| 96439997 | Ethinylestradiol 30microgram / Desogestrel 150microgram tablets |
| 96439998 | Ethinylestradiol 20microgram / Desogestrel 150microgram tablets |
| 96922998 | Ethinylestradiol 30microgram / Gestodene 75microgram tablets |
| 98178998 | Ethinylestradiol 30microgram / Desogestrel 150microgram tablets |
| 90757998 | Ethinylestradiol with gestodene - triphasic 6 x 30+50mcg; 5 x 40+70mcg; 10 x 30+100mcg tablet |
| 97670998 | Generic tri-minulet tablets |
| 97702998 | Generic tri-minulet tablets |

1. Fourth generation combined oral contraceptive pills

| Drug code | Generic name |
| --- | --- |
| 45866978 | Ethinylestradiol 20microgram / Drospirenone 3mg tablets |
| 46090978 | Ethinylestradiol 20microgram / Drospirenone 3mg tablets |
| 47150978 | Ethinylestradiol 30microgram / Drospirenone 3mg tablets |
| 53008979 | Ethinylestradiol 20microgram / Drospirenone 3mg tablets |
| 53009979 | Ethinylestradiol 20microgram / Drospirenone 3mg tablets |
| 72966978 | Ethinylestradiol 30microgram / Drospirenone 3mg tablets |
| 74455978 | Ethinylestradiol 30microgram / Drospirenone 3mg tablets |
| 78546978 | Ethinylestradiol 30microgram / Drospirenone 3mg tablets |
| 83634998 | Ethinylestradiol 20microgram / Drospirenone 3mg tablets |
| 83740978 | Estradiol 1.5mg / Nomegestrol 2.5mg tablets |
| 83741978 | Estradiol 1.5mg / Nomegestrol 2.5mg tablets |
| 84583978 | Ethinylestradiol 20microgram / Drospirenone 3mg tablets |
| 59254978 | Ethinylestradiol 30microgram / Drospirenone 3mg tablets |
| 59255978 | Ethinylestradiol 30microgram / Drospirenone 3mg tablets |
| 82867998 | Generic Qlaira tablets |
| 82869998 | Generic Qlaira tablets |
| 89914979 | Ethinylestradiol 30microgram / Drospirenone 3mg tablets |
| 92571998 | Ethinylestradiol 30microgram / Drospirenone 3mg tablets |
| 98852998 | Ethinylestradiol 30microgram / Drospirenone 3mg tablets |

1. Co-cyprindiol

| Drug code | Generic name |
| --- | --- |
| 47175978 | Co-cyprindiol 2000microgram/35microgram tablets |
| 85864998 | Co-cyprindiol 2000microgram/35microgram tablets |
| 86466998 | Co-cyprindiol 2000microgram/35microgram tablets |
| 86925998 | Co-cyprindiol 2000microgram/35microgram tablets |
| 87351998 | Co-cyprindiol 2000microgram/35microgram tablets |
| 90826979 | Co-cyprindiol 2000microgram/35microgram tablets |
| 90828979 | Co-cyprindiol 2000microgram/35microgram tablets |
| 90833979 | Co-cyprindiol 2000microgram/35microgram tablets |
| 91068998 | Co-cyprindiol 2000microgram/35microgram tablets |
| 91069998 | Co-cyprindiol 2000microgram/35microgram tablets |
| 94832990 | Co-cyprindiol 2000microgram/35microgram tablets |
| 94920998 | Co-cyprindiol 2000microgram/35microgram tablets |
| 95220990 | Co-cyprindiol 2000microgram/35microgram tablets |
| 95396990 | Co-cyprindiol 2000microgram/35microgram tablets |
| 96577998 | Co-cyprindiol 2000microgram/35microgram tablets |
| 97520998 | Co-cyprindiol 2000microgram/35microgram tablets |

1. Progesterone-only pills

| Drug code | Generic name |
| --- | --- |
| 72965978 | Desogestrel 75microgram tablets |
| 53167979 | Desogestrel 75microgram tablets |
| 90581998 | Desogestrel 75microgram tablets |
| 98172998 | Norethisterone 350microgram tablets |
| 83545978 | Desogestrel 75microgram tablets |
| 61400979 | Desogestrel 75microgram tablets |
| 98170998 | Levonorgestrel 30microgram tablets |
| 90580998 | Desogestrel 75microgram tablets |
| 97451998 | Levonorgestrel 75mcg tablets |
| 95699998 | Norgestrel 75microgram tablets |
| 53171979 | Desogestrel 75microgram tablets |
| 85168978 | Desogestrel 75microgram tablets |
| 97599998 | Etynodiol diacetate 500mcg tablets |
| 93986998 | Levonorgestrel 30microgram tablets |
| 53168979 | Desogestrel 75microgram tablets |
| 83189978 | Desogestrel 75microgram tablets |
| 97452998 | Levonorgestrel 30microgram tablets |
| 82528978 | Desogestrel 75microgram tablets |
| 96765998 | Etynodiol 500microgram tablets |
| 91333998 | Levonorgestrel 750microgram tablets |
| 93893998 | Norethisterone 350microgram tablets |
| 53169979 | Desogestrel 75microgram tablets |
| 98174998 | Norethisterone 350microgram tablets |
| 53166979 | Desogestrel 75microgram tablets |

1. Long-acting reversible contraception

| Drug code | Generic name |
| --- | --- |
| 20364978 | Levonorgestrel 19.5mg intrauterine device |
| 20365978 | Levonorgestrel 19.5mg intrauterine device |
| 50916978 | Levonorgestrel 20micrograms/24hours intrauterine device |
| 58042979 | Intrauterine contraceptive device |
| 58043979 | Intrauterine contraceptive device |
| 58044979 | Intrauterine contraceptive device |
| 59356979 | Intrauterine contraceptive device |
| 59358979 | Intrauterine contraceptive device |
| 59359979 | Intrauterine contraceptive device |
| 59360979 | Intrauterine contraceptive device |
| 71058994 | Intrauterine contraceptive device |
| 75898978 | Levonorgestrel 13.5mg intrauterine device |
| 75899978 | Levonorgestrel 13.5mg intrauterine device |
| 80741994 | Intrauterine contraceptive device |
| 83855994 | Intrauterine contraceptive device |
| 83856994 | Intrauterine contraceptive device |
| 83858994 | Intrauterine contraceptive device |
| 83859994 | Intrauterine contraceptive device |
| 84171994 | Intrauterine contraceptive device |
| 86053994 | Intrauterine contraceptive device |
| 87355979 | Intrauterine contraceptive device |
| 87911994 | Intrauterine contraceptive device |
| 89797994 | Intrauterine contraceptive device |
| 89798994 | Intrauterine contraceptive device |
| 90662994 | Intrauterine contraceptive device |
| 91073994 | Intrauterine contraceptive device |
| 91074994 | Intrauterine contraceptive device |
| 91271994 | Intrauterine contraceptive device |
| 91324998 | Levonorgestrel 20micrograms/24hours intrauterine device |
| 91325998 | Levonorgestrel 20micrograms/24hours intrauterine device |
| 92849979 | Levonorgestrel 20micrograms/24hours intrauterine device |
| 92851979 | Levonorgestrel 20micrograms/24hours intrauterine device |
| 95678994 | Intrauterine contraceptive device |
| 97021994 | Intrauterine contraceptive device |
| 97339992 | Intrauterine contraceptive device |
| 97916994 | Intrauterine contraceptive device |
| 97917994 | Intrauterine contraceptive device |
| 98212994 | Intrauterine contraceptive device |
| 99235994 | Intrauterine contraceptive device |
| 99880994 | Intrauterine contraceptive device |
| 81886998 | Etonogestrel 68mg implant |
| 90908998 | Etonogestrel 68mg implant |
| 90909998 | Etonogestrel 68mg implant |
| 92888998 | Levonorgestrel 38mg implant |
| 98222998 | Levonorgestrel 228mg implant |
| 84519978 | Medroxyprogesterone 104mg/0.65ml suspension for injection pre-filled disposable devices |
| 84520978 | Medroxyprogesterone 104mg/0.65ml suspension for injection pre-filled disposable devices |
| 85241998 | Medroxyprogesterone 150mg/1ml suspension for injection pre-filled syringes |
| 85242998 | Medroxyprogesterone 150mg/1ml suspension for injection pre-filled syringes |
| 92842979 | Medroxyprogesterone 150mg/1ml suspension for injection pre-filled syringes |
| 92843979 | Medroxyprogesterone 150mg/1ml suspension for injection pre-filled syringes |
| 92844979 | Medroxyprogesterone 150mg/1ml suspension for injection pre-filled syringes |
| 92846979 | Medroxyprogesterone 150mg/1ml suspension for injection pre-filled syringes |
| 92847979 | Medroxyprogesterone 150mg/1ml suspension for injection pre-filled syringes |
| 95700998 | Norethisterone 200mg/1ml solution for injection ampoules |
| 97454998 | Norethisterone 200mg/1ml solution for injection ampoules |
| 97920998 | Medroxyprogesterone 150mg/1ml suspension for injection pre-filled syringes |
