# Supplementary material for: Use of contraceptives and risk of inflammatory bowel disease: a nested case–control study
Source: Aliment Pharmacol Ther. Author manuscript; Available in PMC 2022 Jun 28. (PMC7612921; doi:10.1111/apt.16647)
Supplement: Supp info 2 [file EMS146182-supplement-Supp_info_2.docx]

**Supporting information - Read code Lists**

1. Any Inflammatory bowel Disease

| Read code | Description |
| --- | --- |
| 14C4.11 | H/O: ulcerative colitis |
| 8Cc5.00 | Management of inflammatory bowel disease |
| 8Cc5.11 | Management of IBD (inflammatory bowel disease) |
| J08z900 | Orofacial Crohn's disease |
| J4...12 | Inflammatory bowel disease |
| J40..00 | Regional enteritis - Crohn's disease |
| J40..11 | Crohn's disease |
| J400.00 | Regional enteritis of the small bowel |
| J400000 | Regional enteritis of the duodenum |
| J400100 | Regional enteritis of the jejunum |
| J400200 | Crohn's disease of the terminal ileum |
| J400300 | Crohn's disease of the ileum unspecified |
| J400400 | Crohn's disease of the ileum NOS |
| J400500 | Exacerbation of Crohn's disease of small intestine |
| J400z00 | Crohn's disease of the small bowel NOS |
| J401.00 | Regional enteritis of the large bowel |
| J401000 | Regional enteritis of the colon |
| J401100 | Regional enteritis of the rectum |
| J401200 | Exacerbation of Crohn's disease of large intestine |
| J401z00 | Crohn's disease of the large bowel NOS |
| J401z11 | Crohn's colitis |
| J402.00 | Regional ileocolitis |
| J40z.00 | Regional enteritis NOS |
| J40z.11 | Crohn's disease NOS |
| J41..12 | Ulcerative colitis and/or proctitis |
| J410.00 | Ulcerative proctocolitis |
| J410100 | Ulcerative colitis |
| J410200 | Ulcerative rectosigmoiditis |
| J410300 | Ulcerative proctitis |
| J410400 | Exacerbation of ulcerative colitis |
| J410z00 | Ulcerative proctocolitis NOS |
| J413.00 | Ulcerative pancolitis |
| J41y.00 | Other idiopathic proctocolitis |
| J41yz00 | Other idiopathic proctocolitis NOS |
| J41z.00 | Idiopathic proctocolitis NOS |
| J4z6.00 | Indeterminate colitis |
| Jyu4000 | [X]Other Crohn's disease |
| Jyu4100 | [X]Other ulcerative colitis |

1. Crohn’s disease

| Read code | Description |
| --- | --- |
| J08z900 | Orofacial Crohn's disease |
| J40..00 | Regional enteritis - Crohn's disease |
| J40..11 | Crohn's disease |
| J400.00 | Regional enteritis of the small bowel |
| J400000 | Regional enteritis of the duodenum |
| J400100 | Regional enteritis of the jejunum |
| J400200 | Crohn's disease of the terminal ileum |
| J400300 | Crohn's disease of the ileum unspecified |
| J400400 | Crohn's disease of the ileum NOS |
| J400500 | Exacerbation of Crohn's disease of small intestine |
| J400z00 | Crohn's disease of the small bowel NOS |
| J401.00 | Regional enteritis of the large bowel |
| J401000 | Regional enteritis of the colon |
| J401100 | Regional enteritis of the rectum |
| J401200 | Exacerbation of Crohn's disease of large intestine |
| J401z00 | Crohn's disease of the large bowel NOS |
| J401z11 | Crohn's colitis |
| J402.00 | Regional ileocolitis |
| J40z.00 | Regional enteritis NOS |
| J40z.11 | Crohn's disease NOS |
| Jyu4000 | [X]Other Crohn's disease |

1. Ulcerative colitis

| Read code | Description |
| --- | --- |
| 14C4.11 | H/O: ulcerative colitis |
| J41..12 | Ulcerative colitis and/or proctitis |
| J410.00 | Ulcerative proctocolitis |
| J410100 | Ulcerative colitis |
| J410200 | Ulcerative rectosigmoiditis |
| J410300 | Ulcerative proctitis |
| J410400 | Exacerbation of ulcerative colitis |
| J410z00 | Ulcerative proctocolitis NOS |
| J413.00 | Ulcerative pancolitis |
| J41y.00 | Other idiopathic proctocolitis |
| J41yz00 | Other idiopathic proctocolitis NOS |
| J41z.00 | Idiopathic proctocolitis NOS |
| Jyu4100 | [X]Other ulcerative colitis |

1. Acne

| Read code | Description |
| --- | --- |
| 2FG5.00 | Acne scar |
| 679g000 | Acne management education |
| M153.00 | Rosacea |
| M153000 | Acne rosacea |
| M153200 | Rosacea hypertrophica |
| M153400 | Ocular rosacea |
| M153z00 | Rosacea NOS |
| M25y600 | Acne keloid |
| M261.00 | Other acne |
| M261000 | Acne vulgaris |
| M261100 | Acne conglobata |
| M261600 | Cystic acne |
| M261A00 | Pustular acne |
| M261B00 | Steroid acne |
| M261C00 | Tropical acne |
| M261F00 | Acne fulminans |
| M261H00 | Acne keloid |
| M261K00 | Acne keloidalis |
| M261L00 | Excoriated acne |
| M261X00 | Acne, unspecified |
| M261z00 | Other acne NOS |
| Myu6800 | [X]Other acne |
| Myu6900 | [X]Other rosacea |
| Myu6F00 | [X]Acne, unspecified |

1. Polycystic ovarian syndrome codes

| Read code | Description |
| --- | --- |
| C164.00 | Polycystic ovaries |
| C164.12 | Stein - Leventhal syndrome |
| C164.13 | Multicystic ovaries |
| C165.00 | Polycystic ovarian syndrome |

1. Endometriosis codes

| Read code | Description |
| --- | --- |
| 7E0D800 | Laparoscopic laser destruction of endometriosis |
| BBL1.11 | [M]Stromal endometriosis |
| K50..00 | Endometriosis |
| K500.00 | Endometriosis of uterus |
| K500000 | Internal endometriosis |
| K500100 | Endometriosis of myometrium |
| K500200 | Endometriosis of cervix |
| K500z00 | Endometriosis of uterus NOS |
| K501.00 | Endometriosis of ovary |
| K502.00 | Endometriosis of the fallopian tube |
| K503.00 | Endometriosis of the pelvic peritoneum |
| K503000 | Endometriosis of the broad ligament |
| K503100 | Endometriosis of the pouch of Douglas |
| K503200 | Endometriosis of the parametrium |
| K503300 | Endometriosis of the round ligament |
| K503z00 | Endometriosis of the pelvic peritoneum NOS |
| K504.00 | Endometriosis of the rectovaginal septum and vagina |
| K504000 | Endometriosis of the rectovaginal septum |
| K504100 | Endometriosis of the vagina |
| K504z00 | Endometriosis of the rectovaginal septum and vagina NOS |
| K505.00 | Endometriosis of the intestine |
| K505000 | Endometriosis of the appendix |
| K505100 | Endometriosis of the colon |
| K505200 | Endometriosis of the rectum |
| K505z00 | Endometriosis of the intestine NOS |
| K506.00 | Endometriosis in scar of skin |
| K50y.00 | Other endometriosis |
| K50y000 | Endometriosis of the bladder |
| K50y100 | Endometriosis of the lung |
| K50y200 | Endometriosis of the umbilicus |
| K50y300 | Endometriosis of the vulva |
| K50yz00 | Other endometriosis NOS |
| K50z.00 | Endometriosis NOS |

1. Pregnancy codes

| Read code | Description |
| --- | --- |
| 62...00 | Patient pregnant |
| 62...11 | Antenatal care |
| 62...12 | Maternity care |
| 62...13 | Pregnancy care |
| 621..00 | Patient currently pregnant |
| 621..11 | Pregnancy confirmed |
| 6211.00 | Pregnant - urine test confirms |
| 6212.00 | Pregnant - blood test confirms |
| 6213.00 | Pregnant - V.E. confirms |
| 6214.00 | Pregnant - on history |
| 6215.00 | Pregnant - on abdom. palpation |
| 6216.00 | Pregnant - planned |
| 6217.00 | Pregnant - unplanned - wanted |
| 6218.00 | Pregnant -unplanned-not wanted |
| 6219.00 | Patient ? pregnant |
| 621A.00 | Pregnancy unplanned ? wanted |
| 621B.00 | Pregnant - ? planned |
| 621C.00 | Unplanned pregnancy |
| 621D.00 | Concealed pregnancy |
| 621Z.00 | Patient pregnant NOS |
| 622..00 | Antenatal care: gravida No. |
| 6221.00 | Antenatal care: primigravida |
| 6222.00 | Antenatal care: 2nd pregnancy |
| 6223.00 | Antenatal care: 3rd pregnancy |
| 6224.00 | Antenatal care: multip |
| 622Z.00 | Antenatal care: gravida NOS |
| 623..00 | A/N care: obstetric risk |
| 6231.00 | A/N care: uncertain dates |
| 6232.00 | A/N care: recurrent aborter |
| 6233.00 | A/N care: grand multip |
| 6234.00 | A/N care: H/O stillbirth |
| 6235.00 | A/N care: H/O perinatal death |
| 6236.00 | A/N care: poor obstetr history |
| 6237.00 | A/N care: H/O trophoblast.dis. |
| 623Z.00 | A/N care: obstetric risk NOS |
| 624..00 | A/N care: precious pregnancy |
| 6241.00 | A/N care: elderly primip. |
| 6242.00 | A/N care: H/O infertility |
| 624Z.00 | A/N care: precious preg. NOS |
| 625..00 | A/N care: social risk |
| 6251.00 | A/N care: poor home conditions |
| 6252.00 | A/N care: poor A/N attender |
| 6253.00 | A/N care: late booker |
| 6254.00 | A/N care: H/O child abuse |
| 625Z.00 | A/N care: social risk NOS |
| 626..00 | A/N care: medical risk |
| 627..00 | A/N care: gynae. risk |
| 628..00 | A/N care: risk NOS |
| 6281.00 | A/N care: under 5ft tall |
| 6282.00 | A/N care:10yrs+since last preg |
| 6283.00 | A/N care: primip. < 17 years |
| 6284.00 | A/N care: primip. > 30 years |
| 6285.00 | A/N care: multip. > 35 years |
| 628Z.00 | A/N risk NOS |
| 629..00 | No ante-natal care |
| 6291.00 | Ante-natal care: not offered |
| 6292.00 | Ante-natal care: not wanted |
| 6293.00 | Ante-natal care: not attended |
| 6294.00 | No A/N care: not known preg. |
| 629Z.00 | No ante-natal care NOS |
| 62A..00 | A/N care provider |
| 62A1.00 | A/N care from G.P. |
| 62A2.00 | A/N care from consultant |
| 62A3.00 | A/N - shared care |
| 62A4.00 | A/N care midwifery led |
| 62AZ.00 | A/N care provider NOS |
| 62B..00 | Delivery booking place |
| 62B1.00 | Delivery: no place booked |
| 62B2.00 | Home delivery booked |
| 62B3.00 | G.P. unit delivery booking |
| 62B4.00 | Consultant unit booking |
| 62B5.00 | Private home delivery booking |
| 62B6.00 | Delivery booking place changed |
| 62B7.00 | Domino delivery |
| 62B8.00 | Midwife unit delivery booking |
| 62BZ.00 | Delivery booking - place NOS |
| 62C..00 | Deliv.booking - length of stay |
| 62C1.00 | Short stay delivery booking |
| 62C2.00 | Full stay delivery booking |
| 62CZ.00 | Delivery booking - stay NOS |
| 62D..00 | Parent craft classes |
| 62D1.00 | Parent craft classes offered |
| 62D2.00 | Parent craft class not offered |
| 62D3.00 | Parent craft not wanted |
| 62D4.00 | Parent craft class attended |
| 62D5.00 | Parent craft -individual class |
| 62D6.00 | Parent craft - group class |
| 62DZ.00 | Parent craft class NOS |
| 62E..00 | Feeding intention |
| 62E..11 | Feeding intention -baby |
| 62E1.00 | Feeding intention - not known |
| 62E2.00 | Feeding intention - unsure |
| 62E3.00 | Feeding intention - breast |
| 62E3.11 | Intends to breast feed |
| 62E4.00 | Feeding intention - bottle |
| 62E4.11 | Intends to bottle feed |
| 62EZ.00 | Feeding intention - NOS |
| 62F..00 | Antenatal amniocentesis |
| 62F1.00 | A/N amniocentesis -not offered |
| 62F2.00 | A/N amniocentesis - offered |
| 62F3.00 | A/N amniocentesis - not wanted |
| 62F4.00 | A/N amniocentesis wanted |
| 62F5.00 | A/N amniocentesis - awaited |
| 62F6.00 | A/N amniocentesis - normal |
| 62F7.00 | A/N amniocentesis - abnormal |
| 62F8.00 | A/N amnio. for ? chrom.abnorm. |
| 62F9.00 | A/N amnio. for ? neural tube |
| 62FZ.00 | Antenatal amniocentesis NOS |
| 62G..00 | Antenatal ultrasound scan |
| 62G1.00 | A/N U/S scan not offered |
| 62G2.00 | A/N U/S scan offered |
| 62G3.00 | A/N U/S scan not wanted |
| 62G4.00 | A/N U/S scan wanted |
| 62G5.00 | A/N U/S scan awaited |
| 62G6.00 | A/N U/S scan normal += dates |
| 62G7.00 | A/N U/S scan normal +? dates |
| 62G8.00 | A/N U/S scan abnormal |
| 62G9.00 | A/N U/S scan for ? abnormality |
| 62GA.00 | A/N U/S scan for slow growth |
| 62GB.00 | Antenatal ultrasounds scan at 4-8 weeks |
| 62GC.00 | Antenatal ultrasound scan at 9-16 weeks |
| 62GD.00 | Antenatal ultrasound scan at 17-22 weeks |
| 62GE.00 | Antenatal ultrasound scan at 22-40 weeks |
| 62GZ.00 | Antenatal ultrasound scan NOS |
| 62H..00 | A/N Rh antibody screen |
| 62H1.00 | A/N Rh screen not offered |
| 62H2.00 | A/N Rh screen offered |
| 62H3.00 | Rh screen - 1st preg. sample |
| 62H4.00 | Rh screen - 2nd preg. sample |
| 62H5.00 | Rh screen - 3rd preg. sample |
| 62H6.00 | Rh screen - cord blood sample |
| 62H7.00 | Rh - 6/12 after anti-D sample |
| 62H8.00 | Rh - random, non-preg. sample |
| 62HZ.00 | A/N Rh antibody screen NOS |
| 62I..00 | Alpha-feto protein blood test |
| 62I..11 | AFP test - antenatal |
| 62I..12 | Alpha-feto protein test - A/N |
| 62I1.00 | AFP blood test offered |
| 62I2.00 | AFP blood test not offered |
| 62I3.00 | AFP blood test wanted |
| 62I4.00 | AFP blood test not wanted |
| 62I5.00 | AFP - blood sent |
| 62IZ.00 | AFP blood test NOS |
| 62J..00 | Rubella screen |
| 62J1.00 | Rubella screen not offered |
| 62J2.00 | Rubella screen offered |
| 62J3.00 | Rubella screen not wanted |
| 62J4.00 | Rubella screen wanted |
| 62J5.00 | Rubella screen - blood sent |
| 62J6.00 | Rubella status not known |
| 62JZ.00 | Rubella screen NOS |
| 62K..00 | Antenatal syphilis screen |
| 62K1.00 | A/N syphilis screen not done |
| 62K2.00 | A/N syphilis screen-blood sent |
| 62KZ.00 | Antenatal syphilis screen NOS |
| 62L..00 | Antenatal blood group screen |
| 62L1.00 | A/N blood gp screen not done |
| 62L2.00 | A/N blood group screen done |
| 62LZ.00 | A/N blood group screen NOS |
| 62M..00 | Antenatal sickle cell screen |
| 62M1.00 | A/N sickle screen not done |
| 62M2.00 | A/N sickle cell screen done |
| 62MZ.00 | A/N sickle cell screen NOS |
| 62N..00 | Antenatal examinations |
| 62N1.00 | A/N booking examination |
| 62N2.00 | A/N 12 weeks examination |
| 62N3.00 | A/N 16 week examination |
| 62N4.00 | A/N 20 week examination |
| 62N5.00 | A/N 24 week examination |
| 62N6.00 | A/N 28 week examination |
| 62N7.00 | A/N 30 week examination |
| 62N8.00 | A/N 32 week examination |
| 62N9.00 | A/N 34 week examination |
| 62NA.00 | A/N 35 week examination |
| 62NB.00 | A/N 36 week examination |
| 62NC.00 | A/N 37 week examination |
| 62ND.00 | A/N 38 week examination |
| 62NE.00 | A/N 39 week examination |
| 62NF.00 | A/N 40 week examination |
| 62NG.00 | A/N 41 week examination |
| 62NH.00 | A/N 42 week examination |
| 62NJ.00 | Antenatal 22 week examination |
| 62NK.00 | Antenatal 25 week examination |
| 62NL.00 | Antenatal 31 week examination |
| 62NZ.00 | Antenatal examination NOS |
| 62O..00 | Misc. antenatal data |
| 62O..11 | Fetal maturity - A/N |
| 62O..12 | Static weight gain pregnancy |
| 62O1.00 | Fetal movements felt |
| 62O1.11 | Quickening |
| 62O2.00 | Fetal movements seen |
| 62O3.00 | Fetal maturity: dates = size |
| 62O4.00 | Fetal maturity: dates not=size |
| 62O5.00 | Spontaneous membrane rupture |
| 62O6.00 | Vaginal "show" |
| 62O6.11 | Vaginal "show" - A/N |
| 62O7.00 | Pregnancy prolonged - 41 weeks |
| 62O8.00 | Pregnancy prolonged - 42 weeks |
| 62O9.00 | Initial booking of patient |
| 62OZ.00 | Misc. antenatal data NOS |
| 62P..00 | Infant feeding method |
| 62P1.00 | Breast fed |
| 62P1.11 | Infant breast fed |
| 62P2.00 | Bottle fed |
| 62P2.11 | Infant bottle fed |
| 62P3.00 | Breast feeding with supplement |
| 62P3000 | Breastfeeding and supplementary bottle feed at dis from hosp |
| 62P4.00 | Breast changed to bottle feed |
| 62P5.00 | Breast feeding started |
| 62P6.00 | Breast feeding stopped |
| 62P7.00 | Bottle feeding started |
| 62P8.00 | Bottle feeding stopped |
| 62P9.00 | Infant weaned |
| 62PA.00 | Mother currently breast feeding |
| 62PB.00 | Bottle changed to breast |
| 62PC.00 | Breast feeding problem |
| 62PD.00 | Lactation established |
| 62PE.00 | Breastfeeding at discharge from hospital |
| 62PF.00 | Bottle feeding at discharge from hospital |
| 62PZ.00 | Infant feeding method NOS |
| 62Q..00 | Postnatal care provider |
| 62Q1.00 | P/N care from consultant |
| 62Q2.00 | P/N care from G.P. |
| 62Q3.00 | P/N - shared care |
| 62Q4.00 | No post natal care |
| 62Q5.00 | P/N care refused |
| 62Q6.00 | Postnatal care |
| 62QZ.00 | Post natal care NOS |
| 62R..00 | Postnatal visits |
| 62R..11 | Postnatal visit |
| 62R..12 | New birth visit |
| 62R1.00 | P/N - first day visit |
| 62R2.00 | P/N - second day visit |
| 62R3.00 | P/N - third day visit |
| 62R4.00 | P/N - fourth day visit |
| 62R5.00 | P/N - fifth day visit |
| 62R6.00 | P/N - sixth day visit |
| 62R7.00 | P/N - seventh day visit |
| 62R8.00 | P/N - eighth day visit |
| 62R9.00 | P/N - ninth day visit |
| 62RA.00 | P/N - tenth day visit |
| 62RB.00 | P/N care started at birth |
| 62RC.00 | P/N care <48hrs after birth |
| 62RD.00 | P/N care >48hrs after birth |
| 62RZ.00 | Postnatal visit NOS |
| 62S..00 | Maternal P/N 6 week exam. |
| 62S..11 | Postnatal exam. - maternal |
| 62S1.00 | Maternal P/N exam. not offered |
| 62S2.00 | Maternal P/N exam. offered |
| 62S3.00 | Maternal P/N exam. refused |
| 62S4.00 | Maternal P/N exam. defaulted |
| 62S5.00 | Maternal P/N exam. done |
| 62S6.00 | Postnatal examination minor problem found |
| 62S7.00 | Postnatal examination normal |
| 62SZ.00 | Maternal P/N 6 week exam. NOS |
| 62T..00 | Misc. postnatal data |
| 62T1.00 | Puerperal depression |
| 62TZ.00 | Misc. post natal data NOS |
| 62U..00 | Downs screen - blood test |
| 62U..11 | Barts test |
| 62U..12 | Triple test |
| 62U..13 | Double test |
| 62U0.00 | Triple test offered |
| 62U1.00 | Double test offered |
| 62U2.00 | Triple test not offered |
| 62U3.00 | Double test not offered |
| 62U4.00 | Triple test wanted |
| 62U5.00 | Double test wanted |
| 62U6.00 | Triple test not wanted |
| 62U7.00 | Double test not wanted |
| 62U8.00 | Downs screening - blood sent |
| 62U9.00 | Downs screen blood test normal |
| 62UA.00 | Downs screen blood test abnormal |
| 62Uz.00 | Downs screening blood test NOS |
| 62V..00 | Delivery place planned |
| 62V0.00 | Home delivery planned |
| 62W..00 | Antenatal blood tests |
| 62X..00 | Length of gestation |
| 62X0.00 | Gestation <24 weeks |
| 62X1.00 | Gestation = 24 weeks |
| 62X2.00 | Gestation >24 weeks |
| 62X3.00 | Full term gestation - 40 weeks |
| 62X4.00 | Length of gestation at birth |
| 62X5.00 | Length of gestation at time of test |
| 62X6.00 | Gestation less than 28 weeks |
| 62Y..00 | Routine antenatal care |
| 62Z..00 | Maternal care NOS |
| 62a..00 | Pregnancy review |
| 62a..11 | Review of pregnancy |
| 62b..00 | Antenatal HIV screening |
| 62c..00 | Antenatal screening |
| 62c0.00 | Crown rump length |
| 63...00 | Birth details |
| 631..00 | Place of birth |
| 631..11 | Born - place delivered |
| 6311.00 | Home birth |
| 6312.00 | GP unit birth |
| 6313.00 | Consultant unit birth |
| 6314.00 | Nursing home birth |
| 6315.00 | Ambulance birth |
| 6316.00 | Born before arrival |
| 6317.00 | Born in transit to hospital |
| 6318.00 | Born in hospital |
| 631Z.00 | Place of birth NOS |
| 632..00 | Length of labour |
| 6321.00 | 1st stage of labour length |
| 6322.00 | 2nd stage of labour length |
| 6323.00 | 3rd stage of labour length |
| 632Z.00 | Length of labour NOS |
| 633..00 | Outcome of delivery |
| 633..11 | Livebirth |
| 633..12 | Stillbirth [prevention record] |
| 633..13 | Triplet birth |
| 633..14 | Twin birth |
| 6331.00 | Single live birth |
| 6332.00 | Single stillbirth |
| 6333.00 | Twins - both live born |
| 6334.00 | Twins - 1 still + 1 live born |
| 6335.00 | Twins - both still born |
| 6336.00 | Triplets - all live born |
| 6337.00 | Triplets -2 live+ 1 still born |
| 6338.00 | Triplets-1 live+ 2 still born |
| 6339.00 | Triplets - 3 still born |
| 633A.00 | Live birth surviving more than one year |
| 633B.00 | Antepartum stillbirth |
| 633C.00 | Intrapartum stillbirth |
| 633D.00 | Order of birth at delivery |
| 633Z.00 | Outcome of delivery NOS |
| 633a.00 | Birth of child |
| 634..00 | Sex of baby |
| 634..11 | Delivery - sex of baby |
| 634..12 | Female baby |
| 634..13 | Male baby |
| 6341.00 | Baby male |
| 6342.00 | Baby female |
| 6343.00 | 2 male babies |
| 6344.00 | 2 female babies |
| 6345.00 | 1 male + 1 female baby |
| 6346.00 | 3 male babies |
| 6347.00 | 2 male + 1 female babies |
| 6348.00 | 1 male + 2 female babies |
| 6349.00 | 3 female babies |
| 634Z.00 | Sex of baby NOS |
| 635..00 | Maturity of baby |
| 635..11 | Full term baby |
| 635..12 | Postmature baby |
| 635..13 | Premature baby |
| 6351.00 | Baby premature 36-38 weeks |
| 6352.00 | Baby v. premature 32-36 weeks |
| 6353.00 | Baby extremely prem.28-32 week |
| 6354.00 | Baby full term maturity |
| 6355.00 | Baby post-mature |
| 6356.00 | Baby premature 26-28 weeks |
| 6357.00 | Baby premature 24-26 weeks |
| 6358.00 | Baby premature 39 weeks |
| 6359.00 | Baby premature 38 weeks |
| 635A.00 | Baby premature 37 weeks |
| 635B.00 | Baby premature 36 weeks |
| 635C.00 | Preterm infant status |
| 635C.11 | Preterm |
| 635Z.00 | Baby maturity NOS |
| 636..00 | Birthweight of baby |
| 636..11 | Birthweight |
| 636..12 | Weight - baby |
| 6361.00 | Baby BW = < 3% (under 2500g) |
| 6362.00 | Baby BW = 3% - 9% (2500-2849g) |
| 6363.00 | Baby BW = 10%-24% (2850-3149g) |
| 6364.00 | Baby BW = 25%-49% (3150-3449g) |
| 6365.00 | Baby BW = 50%-74% (3450-3749g) |
| 6366.00 | Baby BW = 75%-89% (3750-4049g) |
| 6367.00 | Baby BW = 90%-96% (4050-4399g) |
| 6368.00 | Baby BW = > 96% (over 4499g) |
| 6369.00 | Baby BW = 4400 - 4499g |
| 636A.00 | Baby BW = below 751gm |
| 636B.00 | Baby BW = 751g-1kg |
| 636C.00 | Baby BW = 1.0-1.5kg |
| 636D.00 | Baby BW = 1.5-2.0kg |
| 636E.00 | Baby BW = 2.0 - 2.5kg |
| 636F.00 | Baby BW = above 2.5kg |
| 636Z.00 | Birthweight of baby NOS |
| 637..00 | Birth head circumference |
| 6371.00 | Birth HC = < 3rd centile |
| 6372.00 | Birth HC = 3rd-9th centile |
| 6373.00 | Birth HC = 10th-24th centile |
| 6374.00 | Birth HC = 25th-49th centile |
| 6375.00 | Birth HC = 50th-74th centile |
| 6376.00 | Birth HC = 75th-89th centile |
| 6377.00 | Birth HC = 90th-96th centile |
| 6378.00 | Birth HC = > 97th centile |
| 637Z.00 | Birth head circumference NOS |
| 638..00 | Birth length |
| 6381.00 | Birth length = < 3rd centile |
| 6382.00 | Birth length=3rd-9th centile |
| 6383.00 | Birth length=10th-24th centile |
| 6384.00 | Birth length=25th-49th centile |
| 6385.00 | Birth length=50th-74th centile |
| 6386.00 | Birth length=75th-89th centile |
| 6387.00 | Birth length=90th-96th centile |
| 638Z.00 | Birth length = > 97th centile |
| 639..00 | Apgar at 1 minute |
| 6391.00 | Apgar at 1 minute = 0 |
| 6392.00 | Apgar at 1 minute = 1 |
| 6393.00 | Apgar at 1 minute = 2 |
| 6394.00 | Apgar at 1 minute = 3 |
| 6395.00 | Apgar at 1 minute = 4 |
| 6396.00 | Apgar at 1 minute = 5 |
| 6397.00 | Apgar at 1 minute = 6 |
| 6398.00 | Apgar at 1 minute = 7 |
| 6399.00 | Apgar at 1 minute = 8 |
| 639A.00 | Apgar at 1 minute = 9 |
| 639B.00 | Apgar at 1 minute = 10 |
| 639Z.00 | Apgar at 1 minute NOS |
| 63A..00 | Apgar at 5 minutes |
| 63A1.00 | Apgar at 5 minutes = 0 |
| 63A2.00 | Apgar at 5 minutes = 1 |
| 63A3.00 | Apgar at 5 minutes = 2 |
| 63A4.00 | Apgar at 5 minutes = 3 |
| 63A5.00 | Apgar at 5 minutes = 4 |
| 63A6.00 | Apgar at 5 minutes = 5 |
| 63A7.00 | Apgar at 5 minutes = 6 |
| 63A8.00 | Apgar at 5 minutes = 7 |
| 63A9.00 | Apgar at 5 minutes = 8 |
| 63AA.00 | Apgar at 5 minutes = 9 |
| 63AB.00 | Apgar at 5 minutes = 10 |
| 63AZ.00 | Apgar at 5 minutes NOS |
| 63B..00 | Apgar at 10 minutes |
| 63B1.00 | Apgar at 10 minutes = 0 |
| 63B2.00 | Apgar at 10 minutes = 1 |
| 63B3.00 | Apgar at 10 minutes = 2 |
| 63B4.00 | Apgar at 10 minutes = 3 |
| 63B5.00 | Apgar at 10 minutes = 4 |
| 63B6.00 | Apgar at 10 minutes = 5 |
| 63B7.00 | Apgar at 10 minutes = 6 |
| 63B8.00 | Apgar at 10 minutes = 7 |
| 63B9.00 | Apgar at 10 minutes = 8 |
| 63BA.00 | Apgar at 10 minutes = 9 |
| 63BB.00 | Apgar at 10 minutes = 10 |
| 63BZ.00 | Apgar at 10 minutes NOS |
| 63C..00 | Baby misc. "at-risk" factors |
| 63C1.00 | Risk factor - been on SCBU |
| 63C1.11 | Risk factor - been on special care unit |
| 63C2.00 | Bonding problems |
| 63C3.00 | Cot death liability |
| 63C4.00 | Battered baby suspect - FH |
| 63C5.00 | Maternal tobacco abuse |
| 63C6.00 | Maternal drug abuse |
| 63C6.11 | Maternal drug misuse |
| 63C7.00 | Maternal alcohol abuse |
| 63C8.00 | Mother < 20 years old |
| 63C9.00 | Mother has a social worker |
| 63CA.00 | H.V.: mother not managing well |
| 63CB.00 | Risk of non-accidental injury |
| 63CC.00 | Difficult to establish feeding |
| 63CD.00 | High risk infant |
| 63CE.00 | One of twins |
| 63CE.11 | Fraternal twin |
| 63CE.12 | Heterozygous twin |
| 63CE.13 | Identical twin |
| 63CE.14 | Monozygous twin |
| 63CF.00 | One of triplets |
| 63CG.00 | One of quadruplets |
| 63CH.00 | One of quintuplets |
| 63CJ.00 | One of sextuplets |
| 63CK.00 | One of septuplets |
| 63CL.00 | One of multiple birth |
| 63CM.00 | Paternal alcohol abuse |
| 63CZ.00 | Baby "at-risk" factors NOS |
| 63D..00 | Placental details |
| 63D1.00 | Placental weight |
| 63D2.00 | Placenta normal O/E |
| 63D3.00 | Placenta diameter |
| 63D4.00 | Placental infarct |
| 63D5.00 | Placental abnormality |
| 63D6.00 | Placenta incomplete |
| 63D7.00 | Complete placenta at delivery |
| 63DZ.00 | Placental details NOS |
| 63E..00 | Labour details |
| 63E1.00 | Spontaneous onset of labour |
| 63E2.00 | Normal birth |
| 63E3.00 | Normal labour |
| 63F..00 | Birth details not known |
| 63G..00 | Uterine membrane observations |
| 63G0.00 | Membranes complete |
| 63G1.00 | Membranes incomplete |
| 63H..00 | Time of delivery |
| 63Z..00 | Birth details NOS |
| 63Z..11 | Apgar normal |
| 66AX.00 | Diabetes: shared care in pregnancy - diabetol and obstet |
| 7F...00 | Obstetric operations |
| 7F...11 | Childbirth operations |
| 7F...12 | Pregnancy operations |
| 7F...13 | Puerperium operations |
| 7F0..00 | Fetus and gravid uterus operations |
| 7F0..11 | Fetus operations |
| 7F0..12 | Fetus & gravid uterus ops |
| 7F00.00 | Therapeutic fetoscopic operations on fetus |
| 7F00.11 | Therapeutic endoscopic operations on fetus |
| 7F00.12 | Therapeutic foetoscopic operations on fetus |
| 7F00000 | Fetoscopic blood transfusion of fetus |
| 7F00100 | Fetoscopic insert tracheal plug congen diaphragmatic hernia |
| 7F00y00 | Other specified therapeutic fetoscopic operation |
| 7F00z00 | Therapeutic fetoscopic operation NOS |
| 7F01.00 | Diagnostic endoscopic examination of fetus using fetoscope |
| 7F01.11 | Diagnostic endoscopic examination of foetus using fetoscope |
| 7F01000 | Fetoscopic examination of fetus and biopsy of fetus |
| 7F01100 | Fetoscopic examination of fetus and sampling of fetal blood |
| 7F01111 | Foetoscopic examination foetus and sampling of foetal blood |
| 7F01y00 | Diagnostic endoscopic examination fetus using fetoscope OS |
| 7F01z00 | Diagnostic endoscopic examination fetus using fetoscope NOS |
| 7F01z11 | Diagnost endoscopic examination foetus using foetoscope NOS |
| 7F01z12 | Fetoscopy NEC |
| 7F02.00 | Selective destruction of fetus |
| 7F02.11 | Feticide |
| 7F02000 | Early selective feticide |
| 7F02100 | Late selective feticide |
| 7F02200 | Selective feticide NEC |
| 7F02y00 | Other specified selective destruction of fetus |
| 7F02z00 | Selective destruction of fetus NOS |
| 7F03.00 | Therapeutic percutaneous operations on fetus |
| 7F03000 | Percutaneous insertion of fetal vesicoamniotic shunt |
| 7F03100 | Percutaneous insertion of fetal pleuroamniotic shunt |
| 7F03200 | Percutaneous blood transfusion of fetus |
| 7F03300 | Percutaneous insertion of fetal pleural drain |
| 7F03400 | Percutaneous insertion of bladder drain to fetus |
| 7F03500 | Percut insertion trach plug cong diaphragmatic hernia fetus |
| 7F03600 | Percutaneous laser ablation of lesion of fetus |
| 7F03y00 | Other specified therapeutic percutaneous operation on fetus |
| 7F03z00 | Therapeutic percutaneous operation on fetus NOS |
| 7F04.00 | Diagnostic percutaneous examination of fetus |
| 7F04.11 | Diagnostic percutaneous examination of placenta |
| 7F04000 | Percutaneous biopsy of fetus |
| 7F04100 | Percutaneous sampling of fetal blood |
| 7F04111 | Percutaneous sampling of foetal blood |
| 7F04200 | Percutaneous sampling of chorionic villus |
| 7F04300 | Electrode applied to fetal scalp |
| 7F04y00 | Other specified diagnostic percutaneous examination of fetus |
| 7F04z00 | Diagnostic percutaneous examination of fetus NOS |
| 7F05.00 | Other operations on amniotic cavity |
| 7F05000 | Drainage of amniotic cavity |
| 7F05100 | Diagnostic amniocentesis |
| 7F05111 | Amniocentesis NEC |
| 7F05200 | Amnioscopy |
| 7F05300 | Sampling of chorionic villus NEC |
| 7F05400 | Biopsy of placenta NEC |
| 7F05y00 | Other specified other operation on amniotic cavity |
| 7F05z00 | Other operation on amniotic cavity NOS |
| 7F06.00 | Operations on gravid uterus |
| 7F06000 | Cerclage of cervix of gravid uterus |
| 7F06011 | McDonald cerclage of cervix |
| 7F06012 | Shirodkar suture in pregnancy |
| 7F06100 | Removal of cerclage from cervix of gravid uterus |
| 7F06111 | Removal of Shirodkar suture |
| 7F06200 | Repositioning of retroverted gravid uterus |
| 7F06300 | External version of breech |
| 7F06400 | External cephalic version |
| 7F06y00 | Other specified operation on gravid uterus |
| 7F06z00 | Operation on gravid uterus NOS |
| 7F07.00 | Therapeutic endoscopic operations for twin-twin transfusion |
| 7F07.11 | Therap endoscopic operations for twin-twin transfusion syn |
| 7F07000 | Endoscop laser ablation placental arterio-venous anastomosis |
| 7F07100 | Endoscopic serial amnio drainage |
| 7F07200 | End serial drain amnio fluid for twin-twin transfusion synd |
| 7F07y00 | OS therapeutic endoscopic operations twin-twin transfusion |
| 7F07z00 | Therapeutic endoscopic operations twin-twin transfusion NOS |
| 7F08.00 | Therapeutic percutaneous operations twin-twin transfusion |
| 7F08.11 | Therapeutic percutaneous operation twin-twin transfusion syn |
| 7F08000 | Percutan laser ablation placental arterio-venous anastomosis |
| 7F08100 | Percutaneous serial amnio drainage |
| 7F08200 | Perc serial drain amnio fluid for twin-twin transfusion synd |
| 7F08y00 | OS therapeutic percutaneous operations twin-twin transfusion |
| 7F08z00 | Therapeut percutaneous operations twin-twin transfusion NOS |
| 7F09.00 | Destruction of fetus |
| 7F09y00 | Other specified destruction of fetus |
| 7F09z00 | Unspecified destruction of fetus |
| 7F0y.00 | Other specified operations on fetus or gravid uterus |
| 7F0z.00 | Fetus and gravid uterus operations NOS |
| 7F1..00 | Induction and delivery operations |
| 7F1..11 | Labour operations |
| 7F10.00 | Surgical induction of labour |
| 7F10000 | Fore water rupture of amniotic membrane |
| 7F10100 | Hind water rupture of amniotic membrane |
| 7F10y00 | Other specified surgical induction of labour |
| 7F10z00 | Surgical induction of labour NOS |
| 7F10z11 | Artificial rupture of membranes |
| 7F10z12 | ARM |
| 7F11.00 | Other induction of labour |
| 7F11000 | Oxytocic induction of labour |
| 7F11100 | Induction of labour using prostaglandins |
| 7F11200 | Syntocinon induction of labour |
| 7F11300 | Medical induction of labour |
| 7F11400 | Sweeping of membrane |
| 7F11y00 | Other specified other induction of labour |
| 7F11z00 | Other induction of labour NOS |
| 7F12.00 | Elective caesarean delivery |
| 7F12000 | Elective upper uterine segment caesarean delivery |
| 7F12100 | Elective lower uterine segment caesarean delivery |
| 7F12111 | Elective lower uterine segment caesarean section (LSCS) |
| 7F12y00 | Other specified elective caesarean delivery |
| 7F12z00 | Elective caesarean delivery NOS |
| 7F13.00 | Other caesarean delivery |
| 7F13000 | Upper uterine segment caesarean delivery NEC |
| 7F13100 | Lower uterine segment caesarean delivery NEC |
| 7F13111 | Lower uterine segment caesarean section (LSCS) NEC |
| 7F13200 | Extraperitoneal caesarean section |
| 7F13300 | Emergency caesarean section |
| 7F13y00 | Other specified other caesarean delivery |
| 7F13z00 | Other caesarean delivery NOS |
| 7F14.00 | Breech extraction delivery |
| 7F14000 | Breech extraction delivery with version |
| 7F14100 | Forceps to aftercoming head (breech) |
| 7F14y00 | Other specified breech extraction delivery |
| 7F14z00 | Breech extraction delivery NOS |
| 7F15.00 | Other breech delivery |
| 7F15000 | Spontaneous breech delivery |
| 7F15100 | Assisted breech delivery |
| 7F15y00 | Other specified other breech delivery |
| 7F15z00 | Other breech delivery NOS |
| 7F16.00 | Forceps cephalic delivery |
| 7F16000 | High forceps cephalic delivery with rotation |
| 7F16100 | High forceps cephalic delivery NEC |
| 7F16200 | Mid forceps cephalic delivery with rotation |
| 7F16300 | Mid forceps cephalic delivery NEC |
| 7F16400 | Low forceps cephalic delivery |
| 7F16500 | Trial of forceps delivery |
| 7F16600 | Failed forceps delivery |
| 7F16700 | Barton forceps cephalic delivery with rotation |
| 7F16800 | Dehee forceps cephalic delivery with rotation |
| 7F16900 | Kielland forceps cephalic delivery with rotation |
| 7F16A00 | Scanzoni forceps cephalic delivery with rotation |
| 7F16B00 | Piper forceps delivery |
| 7F16y00 | Other specified forceps cephalic delivery |
| 7F16z00 | Forceps cephalic delivery NOS |
| 7F17.00 | Vacuum delivery |
| 7F17.11 | Ventouse delivery |
| 7F17.12 | Ventouse extraction |
| 7F17000 | High vacuum delivery |
| 7F17100 | Low vacuum delivery |
| 7F17200 | Vacuum delivery before full dilation of cervix |
| 7F17300 | Trial of vacuum delivery |
| 7F17y00 | Other specified vacuum delivery |
| 7F17z00 | Vacuum delivery NOS |
| 7F18.00 | Cephalic vaginal deliv abnorm presentation head - no instrum |
| 7F18000 | Manip cephalic vaginal deliv abnorm pres head without instrm |
| 7F18100 | Nonmanip cephal vagin deliv abnorm pres head without instrum |
| 7F18y00 | Cephalic vagin deliv abnorm pres head without instrument OS |
| 7F18z00 | Cephalic vagin deliv abnorm pres head without instrument NOS |
| 7F19.00 | Normal delivery |
| 7F19000 | Manually assisted vaginal delivery |
| 7F19100 | Water birth delivery |
| 7F19y00 | Other specified normal delivery |
| 7F19z00 | Normal delivery NOS |
| 7F1A.00 | Other methods of delivery |
| 7F1A000 | Caesarean hysterectomy |
| 7F1A100 | Destructive operation to facilitate delivery |
| 7F1A200 | Cleidotomy of fetus to facilitate delivery |
| 7F1A300 | Drainage of hydrocephalus of fetus to facilitate delivery |
| 7F1A400 | Trial of labour NEC |
| 7F1Ay00 | Other specified other method of delivery |
| 7F1Az00 | Other method of delivery NOS |
| 7F1B.00 | Other operations to facilitate delivery |
| 7F1B000 | Episiotomy to facilitate delivery |
| 7F1B100 | Symphysiotomy to facilitate delivery |
| 7F1B200 | Pubiotomy to facilitate delivery |
| 7F1B300 | Manual dilatation of cervix |
| 7F1B400 | Incision of cervix to facilitate delivery |
| 7F1B500 | Deinfibulation of vulva to facilitate delivery |
| 7F1By00 | Other specified other operation to facilitate delivery |
| 7F1Bz00 | Other operation to facilitate delivery NOS |
| 7F1y.00 | Other specified induction or delivery operations |
| 7F1z.00 | Induction and delivery operations NOS |
| 7F2..00 | Other obstetric operations |
| 7F20.00 | Instrument removal retained products conception deliv uterus |
| 7F20000 | Curettage of delivered uterus |
| 7F20y00 | Instrumental removal products of concep delivered uterus OS |
| 7F20z00 | Instrumental removal products of concep delivered uterus NOS |
| 7F21.00 | Manual removal retained products conception delivered uterus |
| 7F21000 | Manual removal of placenta from delivered uterus |
| 7F21y00 | Manual removal products of conception delivered uterus OS |
| 7F21z00 | Manual removal products of conception delivered uterus NOS |
| 7F22.00 | Other operations on delivered uterus |
| 7F22000 | Repositioning of inverted delivered uterus |
| 7F22100 | Expression of placenta |
| 7F22111 | Brandt-Andrews expression of placenta |
| 7F22200 | Instrumental exploration of delivered uterus NEC |
| 7F22300 | Manual exploration of delivered uterus NEC |
| 7F22500 | Normal delivery of placenta |
| 7F22600 | Crede placental expression |
| 7F22700 | Pack to control postnatal vaginal bleeding |
| 7F22711 | Pack to control postnatal vaginal bleeding |
| 7F22712 | Pack to control postnatal haemorrhage |
| 7F22713 | Pack to control postpartum haemorrhage |
| 7F22y00 | Other specified other operation on delivered uterus |
| 7F22z00 | Other operation on delivered uterus NOS |
| 7F23.00 | Immediate repair of obstetric laceration |
| 7F23.11 | Immediate repair of obstetric tear |
| 7F23.12 | Immediate suture of obstetric laceration |
| 7F23000 | Immed repair obstetric laceration of uterus or cervix uteri |
| 7F23100 | Immed repair obstetric laceration perineum & anal sphincter |
| 7F23200 | Immed repair obstetric laceration vagina and floor of pelvis |
| 7F23300 | Immediate repair of minor obstetric laceration |
| 7F23400 | Repair of episiotomy |
| 7F23500 | Repair of ruptured uterus |
| 7F23600 | Rep obstet lacerat perineum and sphincter and mucosa of anus |
| 7F23611 | Rep obstet lacer perineum and anal sphinct and mucosa rectum |
| 7F23y00 | Other specified immediate repair of obstetric laceration |
| 7F23z00 | Immediate repair of obstetric laceration NOS |
| 7F24.00 | Other obstetric operations |
| 7F24000 | Secondary repair of obstetric laceration |
| 7F24100 | Obstetric uterine tamponade |
| 7F24200 | Repositioning of umbilical cord |
| 7F24y00 | Other specified other obstetric operation |
| 7F24z00 | Other obstetric operation NOS |
| 7F25.00 | Obstetric monitoring |
| 7F25.11 | Fetal monitoring |
| 7F25.12 | Foetal monitoring |
| 7F25.13 | Monitoring during labour |
| 7F25000 | Fetal heart monitoring NEC |
| 7F25100 | Fetal heart monitoring in labour |
| 7F25200 | Cardiotocography |
| 7F25211 | Cardiotochogram |
| 7F25y00 | Other specified obstetric monitoring |
| 7F25z00 | Obstetric monitoring NOS |
| 7F26.00 | Routine obstetric scan |
| 7F26000 | Dating scan |
| 7F26100 | Viability scan |
| 7F26200 | Mid trimester scan |
| 7F26y00 | Other specified routine obstetric scan |
| 7F26z00 | Routine obstetric scan NOS |
| 7F27.00 | Non routine obstetric scan for fetal observations |
| 7F27000 | Biophysical profile |
| 7F27100 | Detailed structural scan |
| 7F27200 | Fetal biometry |
| 7F27300 | Nuchal translucency scan |
| 7F27400 | Fetal ascites scan |
| 7F27500 | Rhesus detailed scan |
| 7F27y00 | OS non routine obstetric scan for fetal observations |
| 7F27z00 | Non routine obstetric scan for fetal observations NOS |
| 7F28.00 | Other non routine obstetric scan |
| 7F28000 | Placental localisation scan |
| 7F28100 | Liquor volume scan |
| 7F28y00 | Other specified other non routine obstetric scan |
| 7F28z00 | Other non routine obstetric scan NOS |
| 7F29.00 | Other maternal physiological assessments |
| 7F29000 | Maternal cervical assessment |
| 7F29100 | Cervical length scanning at 24 weeks |
| 7F29y00 | Other specified other maternal physiological assessments |
| 7F29z00 | Other maternal physiological assessments NOS |
| 7F2A.00 | Obstetric Doppler studies |
| 7F2A000 | Obstetric umbilical artery Doppler |
| 7F2A011 | Doppler ultrasound scan of umbilical artery |
| 7F2A100 | Obstetric uterine artery Doppler |
| 7F2A111 | Doppler ultrasound scan of uterine artery |
| 7F2A200 | Obstetric middle cerebral artery Doppler |
| 7F2A211 | Doppler ultrasound scan of middle cerebral artery of fetus |
| 7F2Ay00 | Other specified obstetric Doppler studies |
| 7F2Ay11 | Other specified obstetric doppler ultrasound |
| 7F2Az00 | Obstetric Doppler studies NOS |
| 7F2Az11 | Obstetric doppler ultrasound NOS |
| 7F2B.00 | Obstetric ultrasound monitoring |
| 7F2B000 | Ultrasound monitoring of luteal phase |
| 7F2B100 | Ultrasound monitoring of early pregnancy |
| 7F2By00 | Other specified obstetric ultrasound monitoring |
| 7F2Bz00 | Obstetric ultrasound monitoring NOS |
| 7F2y.00 | Other specified obstetric operations |
| 7F2z.00 | Other obstetric operations NOS |
| 7Fy..00 | Other specified obstetric operations |
| 7Fz..00 | Obstetric operations NOS |
| L0...00 | Pregnancy with abortive outcome |
| L00..00 | Hydatidiform mole |
| L00..11 | Trophoblastic disease |
| L00..12 | Vesicular mole |
| L000.00 | Classical hydatidiform mole |
| L001.00 | Incomplete and partial hydatidiform mole |
| L002.00 | Complete hydatidiform mole |
| L01..00 | Other abnormal product of conception |
| L010.00 | Blighted ovum |
| L010.11 | Anembryonic pregnancy |
| L011.00 | Carneous mole |
| L011.11 | Fleshy mole |
| L01z.00 | Other abnormal product of conception NOS |
| L02..00 | Missed abortion |
| L02..11 | Missed miscarriage |
| L02..12 | Silent miscarriage |
| L03..00 | Ectopic pregnancy |
| L030.00 | Abdominal pregnancy |
| L030000 | Delivery of viable fetus in abdominal pregnancy |
| L031.00 | Tubal pregnancy |
| L031000 | Fallopian tube pregnancy |
| L031100 | Gravid fallopian tube rupture |
| L031200 | Tubal abortion |
| L031z00 | Tubal pregnancy NOS |
| L032.00 | Ovarian pregnancy |
| L03y.00 | Other ectopic pregnancy |
| L03y000 | Cervical pregnancy |
| L03y100 | Cornual pregnancy |
| L03y200 | Membranous pregnancy |
| L03y300 | Combined or heterotopic pregnancy |
| L03y400 | Mural pregnancy |
| L03y500 | Intraligamentous pregnancy |
| L03y600 | Mesenteric pregnancy |
| L03y700 | Angular pregnancy |
| L03y800 | Mesometric pregnancy |
| L03yz00 | Other ectopic pregnancy NOS |
| L03z.00 | Ectopic pregnancy NOS |
| L04..00 | Spontaneous abortion |
| L04..11 | Miscarriage |
| L040.00 | Spontaneous abortion unspecified |
| L040000 | Unspec spontaneous abortion + genital tract/pelvic infection |
| L040011 | Spontaneous abortion with sepsis |
| L040100 | Unspec spontaneous abortion + delayed/excessive haemorrhage |
| L040111 | Spontaneous abortion with heavy bleeding |
| L040200 | Unspec spontaneous abortion + pelvic organ/tissue damage |
| L040300 | Unspecified spontaneous abortion with renal failure |
| L040400 | Unspecified spontaneous abortion with metabolic disorder |
| L040500 | Unspecified spontaneous abortion with shock |
| L040600 | Unspecified spontaneous abortion with embolism |
| L040900 | Inevitable miscarriage |
| L040w00 | Unspec spontaneous abortion + other specified complication |
| L040x00 | Unspecified spontaneous abortion with complication NOS |
| L040y00 | Unspec spontaneous abortion without mention of complication |
| L040z00 | Unspecified spontaneous abortion NOS |
| L041.00 | Spontaneous abortion incomplete |
| L041000 | Incomp spontaneous abortion + genital tract/pelvic infection |
| L041100 | Incomp spontaneous abortion + delayed/excessive haemorrhage |
| L041200 | Incomplete spontaneous abortion + pelvic organ/tissue damage |
| L041300 | Incomplete spontaneous abortion with renal failure |
| L041400 | Incomplete spontaneous abortion with metabolic disorder |
| L041500 | Incomplete spontaneous abortion with shock |
| L041600 | Incomplete spontaneous abortion with embolism |
| L041w00 | Incomp spontaneous abortion + other specified complication |
| L041x00 | Incomplete spontaneous abortion with complication NOS |
| L041y00 | Incomp spontaneous abortion with no mention of complication |
| L041z00 | Incomplete spontaneous abortion NOS |
| L041z11 | Retained products after spontaneous abortion |
| L042.00 | Spontaneous abortion complete |
| L042000 | Complete spontaneous abortion + genital tract/pelvic infect |
| L042100 | Complete spontaneous abortion +delayed/excessive haemorrhage |
| L042200 | Complete spontaneous abortion + pelvic organ/tissue damage |
| L042300 | Complete spontaneous abortion with renal failure |
| L042400 | Complete spontaneous abortion with metabolic disorder |
| L042500 | Complete spontaneous abortion with shock |
| L042600 | Complete spontaneous abortion with embolism |
| L042w00 | Complete spontaneous abortion + other specified complication |
| L042x00 | Complete spontaneous abortion with complication NOS |
| L042y00 | Complete spontaneous abortion + no mention of complication |
| L042z00 | Complete spontaneous abortion NOS |
| L043.00 | Inevitable abortion unspecified |
| L043.11 | Inevitable miscarriage unspecified |
| L043000 | Unspec inev abor comp by genital tract and pelvic infect |
| L043011 | Unspec inev miscarriage comp by genital tract pelvic infec |
| L043100 | Unspec inevit abortion comp by delayed or excessive haemorr |
| L043111 | Unsp inevitable mis comp by delayed or excessive haemorrhage |
| L043200 | Unspecified inevitable abortion complicated by embolism |
| L043211 | Unspecified inevitable miscarriage complicated by embolism |
| L043x00 | Unspecified inevitable abortion with unspec complication |
| L043x11 | Unspecified inevitable miscarriage with unspec complication |
| L043y00 | Unspecified inevitable abortion with OS complication |
| L043y11 | Unspecified inevitable miscarriage with OS complication |
| L043z00 | Unspecified inevitable abortion without complication |
| L043z11 | Unspecified inevitable miscarriage without complication |
| L044.00 | Inevitable abortion incomplete |
| L044.11 | Inevitable miscarriage incomp |
| L044000 | Incomp inev abor comp by genital tract and pelvic infection |
| L044011 | Incomp inev mis complicated by genital tract pelvic infect |
| L044100 | Incom inev abor complicated by delayed or excessive haemorr |
| L044111 | Incomplete inev mis comp by delayed or excessive haemorrhage |
| L044200 | Incomplete inevitable abortion complicated by embolism |
| L044211 | Incomplete inevitable abortion complicated by embolism |
| L044x00 | Incomplete inevitable abortion with unspecified complication |
| L044x11 | Incomplete inevitable miscarriage with unspecified comp |
| L044y00 | Incomplete inevitable abortion with OS complication |
| L044y11 | Incomplete inevitable miscarriage with other specified comp |
| L044z00 | Incomplete inevitable abortion without complication |
| L044z11 | Incomplete inevitable miscarriage without complication |
| L045.00 | Inevitable abortion complete |
| L045.11 | Inevitable miscarriage complete |
| L045000 | Complete inev abor comp by genital tract and pelvic infec |
| L045011 | Complete inev misc compl by genital tract and pelvic infec |
| L045100 | Complete inevitable abor comp by delayed or excessive haem |
| L045111 | Complete inevitable miscar comp by delayed or excessive haem |
| L045200 | Complete inevitable abortion complicated by embolism |
| L045211 | Complete inevitable miscarriage complicated by embolism |
| L045x00 | Complete inevitable abortion with unspecified complication |
| L045x11 | Complete inevitable miscarriage with unspecified comp |
| L045y00 | Complete inevitable abortion with OS complication |
| L045y11 | Complete inevitable miscarriage with OS complication |
| L045z00 | Complete inevitable abortion without complication |
| L045z11 | Complete inevitable miscarriage without complication |
| L04z.00 | Spontaneous abortion NOS |
| L05..00 | Legally induced abortion |
| L05..11 | Elective abortion |
| L05..12 | Termination of pregnancy |
| L05..13 | Therapeutic abortion |
| L050.00 | Legal abortion unspecified |
| L050000 | Unspecified legal abortion + genital tract/pelvic infection |
| L050100 | Unspecified legal abortion + delayed/excessive haemorrhage |
| L050200 | Unspecified legal abortion + damage to pelvic organs/tissues |
| L050300 | Unspecified legal abortion with renal failure |
| L050400 | Unspecified legal abortion with metabolic disorder |
| L050500 | Unspecified legal abortion with shock |
| L050600 | Unspecified legal abortion with embolism |
| L050w00 | Unspecified legal abortion with other specified complication |
| L050x00 | Unspecified legal abortion with complication NOS |
| L050y00 | Unspecified legal abortion with no mention of complication |
| L050z00 | Unspecified legal abortion NOS |
| L051.00 | Legal abortion incomplete |
| L051.11 | Medal abortion - incomplete |
| L051.12 | Surgical abortion - incomplete |
| L051000 | Incomplete legal abortion + genital tract/pelvic infection |
| L051100 | Incomplete legal abortion + delayed or excessive haemorrhage |
| L051200 | Incomplete legal abortion + damage to pelvic organs/tissues |
| L051300 | Incomplete legal abortion with renal failure |
| L051400 | Incomplete legal abortion with metabolic disorder |
| L051500 | Incomplete legal abortion with shock |
| L051600 | Incomplete legal abortion with embolism |
| L051700 | Incomplete medical abortion |
| L051711 | Incomplete termination of pregnancy |
| L051w00 | Incomplete legal abortion with other specified complication |
| L051x00 | Incomplete legal abortion with complication NOS |
| L051y00 | Incomplete legal abortion with no mention of complication |
| L051z00 | Incomplete legal abortion NOS |
| L052.00 | Legal abortion complete |
| L052.11 | Medical abortion - complete |
| L052.12 | Surgical abortion - complete |
| L052000 | Complete legal abortion + genital tract or pelvic infection |
| L052100 | Complete legal abortion with delayed/excessive haemorrhage |
| L052200 | Complete legal abortion + damage to pelvic organs or tissues |
| L052300 | Complete legal abortion with renal failure |
| L052400 | Complete legal abortion with metabolic disorder |
| L052500 | Complete legal abortion with shock |
| L052600 | Complete legal abortion with embolism |
| L052w00 | Complete legal abortion with other specified complication |
| L052x00 | Complete legal abortion with complication NOS |
| L052y00 | Complete legal abortion with no mention of complication |
| L052z00 | Complete legal abortion NOS |
| L05z.00 | Legally induced abortion NOS |
| L06..00 | Illegally induced abortion |
| L06..11 | Criminal abortion |
| L06..12 | Self-induced abortion |
| L060.00 | Illegal abortion unspecified |
| L060000 | Unspec illegal abortion + genital tract or pelvic infection |
| L060100 | Unspec illegal abortion + delayed or excessive haemorrhage |
| L060200 | Unspecified illegal abortion + pelvic organ/tissue damage |
| L060300 | Unspecified illegal abortion with renal failure |
| L060400 | Unspecified illegal abortion with metabolic disorder |
| L060500 | Unspecified illegal abortion with shock |
| L060600 | Unspecified illegal abortion with embolism |
| L060w00 | Unspecified illegal abortion + other specified complication |
| L060x00 | Unspecified illegal abortion with complication NOS |
| L060y00 | Unspecified illegal abortion with no mention of complication |
| L060z00 | Unspecified illegal abortion NOS |
| L061.00 | Illegal abortion incomplete |
| L061000 | Incomplete illegal abortion + genital tract/pelvic infection |
| L061100 | Incomplete illegal abortion + delayed/excessive haemorrhage |
| L061200 | Incomplete illegal abortion + pelvic organ/tissue damage |
| L061300 | Incomplete illegal abortion with renal failure |
| L061400 | Incomplete illegal abortion with metabolic disorder |
| L061500 | Incomplete illegal abortion with shock |
| L061600 | Incomplete illegal abortion with embolism |
| L061w00 | Incomplete illegal abortion + other specified complication |
| L061x00 | Incomplete illegal abortion with complication NOS |
| L061y00 | Incomplete illegal abortion with no mention of complication |
| L061z00 | Incomplete illegal abortion NOS |
| L062.00 | Illegal abortion complete |
| L062000 | Complete illegal abortion + genital tract/pelvic infection |
| L062100 | Complete illegal abortion + delayed or excessive haemorrhage |
| L062200 | Complete illegal abortion + pelvic organ/tissue damage |
| L062300 | Complete illegal abortion with renal failure |
| L062400 | Complete illegal abortion with metabolic disorder |
| L062500 | Complete illegal abortion with shock |
| L062600 | Complete illegal abortion with embolism |
| L062w00 | Complete illegal abortion with other specified complication |
| L062x00 | Complete illegal abortion with complication NOS |
| L062y00 | Complete illegal abortion with no mention of complication |
| L062z00 | Complete illegal abortion NOS |
| L06z.00 | Illegally induced abortion NOS |
| L07..00 | Unspecified abortion |
| L070.00 | Unspecified abortion |
| L070000 | Unspecified abortion with genital tract or pelvic infection |
| L070100 | Unspecified abortion with delayed or excessive haemorrhage |
| L070200 | Unspecified abortion with damage to pelvic organs or tissues |
| L070300 | Unspecified abortion with renal failure |
| L070400 | Unspecified abortion with metabolic disorder |
| L070500 | Unspecified abortion with shock |
| L070600 | Unspecified abortion with embolism |
| L070w00 | Unspecified abortion with other specified complication |
| L070x00 | Unspecified abortion with complication NOS |
| L070y00 | Unspecified abortion with no mention of complication |
| L070z00 | Unspecified abortion NOS |
| L071.00 | Unspecified abortion incomplete |
| L071000 | Unspecified incomplete abortion +genital tract/pelvic infect |
| L071100 | Unspecified incomplete abortion + delayed/excess haemorrhage |
| L071200 | Unspecified incomplete abortion + pelvic organ/tissue damage |
| L071300 | Unspecified incomplete abortion with renal failure |
| L071400 | Unspecified incomplete abortion with metabolic disorder |
| L071500 | Unspecified incomplete abortion with shock |
| L071600 | Unspecified incomplete abortion with embolism |
| L071w00 | Unspec incomplete abortion with other specified complication |
| L071x00 | Unspecified incomplete abortion with complication NOS |
| L071y00 | Unspecified incomplete abortion + no mention of complication |
| L071z00 | Unspecified incomplete abortion NOS |
| L072.00 | Unspecified abortion complete |
| L072000 | Unspecified complete abortion + genital tract/pelvic infect |
| L072100 | Unspecified complete abortion +delayed/excessive haemorrhage |
| L072200 | Unspecified complete abortion + pelvic organ/tissue damage |
| L072300 | Unspecified complete abortion with renal failure |
| L072400 | Unspecified complete abortion with metabolic disorder |
| L072500 | Unspecified complete abortion with shock |
| L072600 | Unspecified complete abortion with embolism |
| L072w00 | Unspecified complete abortion + other specified complication |
| L072x00 | Unspecified complete abortion with complication NOS |
| L072y00 | Unspecified complete abortion + no mention of complication |
| L072z00 | Unspecified complete abortion NOS |
| L07z.00 | Unspecified abortion NOS |
| L08..00 | Failed attempted abortion |
| L080.00 | Failed attempted abortion + genital tract/pelvic infection |
| L081.00 | Failed attempted abortion + delayed or excessive haemorrhage |
| L082.00 | Failed attempted abortion + damage to pelvic organs/tissues |
| L083.00 | Failed attempted abortion with renal failure |
| L084.00 | Failed attempted abortion with metabolic disorder |
| L085.00 | Failed attempted abortion with shock |
| L086.00 | Failed attempted abortion with embolism |
| L08w.00 | Failed attempted abortion with other specified complication |
| L08x.00 | Failed attempted abortion with complication NOS |
| L08y.00 | Failed attempted abortion with no mention of complication |
| L08z.00 | Failed attempted abortion NOS |
| L09..00 | Complications following abortion/ectopic/molar pregnancies |
| L09..11 | Complications following abortion/ectopic/molar pregnancies |
| L090.00 | Genital or pelvic infection following abortive pregnancy |
| L090000 | Endometritis following abortive pregnancy |
| L090100 | Parametritis following abortive pregnancy |
| L090200 | Pelvic peritonitis following abortive pregnancy |
| L090300 | Salpingitis following abortive pregnancy |
| L090400 | Salpingo-oophoritis following abortive pregnancy |
| L090y00 | Sepsis NOS following abortion/ectopic/molar pregnancy |
| L090z00 | Septicaemia NOS following abortive pregnancy |
| L091.00 | Delayed/excessive haemorrhage following abortive pregnancy |
| L091000 | Afibrinogenaemia following abortive pregnancy |
| L091100 | Defibrination syndrome following abortive pregnancy |
| L091200 | Intravascular haemolysis following abortive pregnancy |
| L091z00 | Delayed/excess haemorrhage NOS following abortive pregnancy |
| L092.00 | Pelvic organ or tissue damage following abortive pregnancy |
| L092000 | Bladder damage following abortive pregnancy |
| L092100 | Bowel damage following abortive pregnancy |
| L092200 | Broad ligament damage following abortive pregnancy |
| L092300 | Cervix damage following abortive pregnancy |
| L092400 | Periurethral tissue damage following abortive pregnancy |
| L092500 | Uterus damage following abortive pregnancy |
| L092600 | Vaginal damage following abortive pregnancy |
| L092z00 | Pelvic organ or tissue damage NOS follow abortive pregnancy |
| L093.00 | Renal failure following abortive pregnancy |
| L093000 | Oliguria following abortive pregnancy |
| L093100 | Acute renal failure following abortive pregnancy |
| L093200 | Renal shutdown following abortive pregnancy |
| L093300 | Renal tubular necrosis following abortive pregnancy |
| L093400 | Uraemia following abortive pregnancy |
| L093z00 | Renal failure NOS following abortive pregnancy |
| L094.00 | Metabolic disorder following abortive pregnancy |
| L095.00 | Shock following abortive pregnancy |
| L096.00 | Embolism following abortive pregnancy |
| L096.11 | Embolus following abortive pregnancy |
| L096000 | Air embolism following abortive pregnancy |
| L096100 | Amniotic fluid embolism following abortive pregnancy |
| L096200 | Blood-clot embolism following abortive pregnancy |
| L096300 | Fat embolism following abortive pregnancy |
| L096400 | Pulmonary embolism following abortive pregnancy |
| L096500 | Pyaemic embolism following abortive pregnancy |
| L096600 | Septic embolism following abortive pregnancy |
| L096700 | Soap embolism following abortive pregnancy |
| L096z00 | Embolism NOS following abortive pregnancy |
| L097.00 | Readmission for abortive pregnancy (NHS codes) |
| L097.11 | Readmission for retained products of conception (NHS codes) |
| L097000 | Readmis for retain products of concept, spontaneous abortion |
| L097100 | Readmission for retained produc of concept, legal abortion |
| L097200 | Readmission for retained produc of concept, illegal abortion |
| L097300 | Readmission for retained produc of concept, unspec abortion |
| L09y.00 | Other specified complication following abortive pregnancy |
| L09y000 | Acute liver necrosis following abortive pregnancy |
| L09y100 | Cardiac arrest following abortive pregnancy |
| L09y200 | Cardiac failure following abortive pregnancy |
| L09y300 | Cerebral anoxia following abortive pregnancy |
| L09y400 | Urinary tract infection following abortive pregnancy |
| L09yz00 | Other specified complication NOS follow abortive pregnancy |
| L09z.00 | Complication NOS following abortion/ectopic/molar pregnancy |
| L0A..00 | Failed attempted abortion |
| L0A1.00 | Failed medical abortion complic by genital tract/pelvic infn |
| L0A2.00 | Failed medical abortion comp by delayed/excessive haem'ge |
| L0A3.00 | Failed medical abortion, complicated by embolism |
| L0A4.00 | Failed medical abortion, without complication |
| L0y..00 | Other specified pregnancy with abortive outcome |
| L0z..00 | Pregnancy with abortive outcome NOS |
| L1...00 | Pregnancy complications |
| L10..00 | Haemorrhage in early pregnancy |
| L100.00 | Threatened abortion |
| L100000 | Threatened abortion unspecified |
| L100100 | Threatened abortion - delivered |
| L100200 | Threatened abortion - not delivered |
| L100z00 | Threatened abortion NOS |
| L10y.00 | Other haemorrhage in early pregnancy |
| L10y.11 | Bleeding in early pregnancy |
| L10y000 | Other haemorrhage in early pregnancy unspecified |
| L10y100 | Other haemorrhage in early pregnancy - delivered |
| L10y200 | Other haemorrhage in early pregnancy - not delivered |
| L10yz00 | Other haemorrhage in early pregnancy NOS |
| L10z.00 | Early pregnancy haemorrhage NOS |
| L10z000 | Early pregnancy haemorrhage NOS unspecified |
| L10z100 | Early pregnancy haemorrhage NOS - delivered |
| L10z200 | Early pregnancy haemorrhage NOS - not delivered |
| L10zz00 | Early pregnancy haemorrhage NOS |
| L10zz11 | Inevitable abortion |
| L11..00 | Antepartum haemorrhage, abruptio placentae, placenta praevia |
| L11..11 | Antepartum haemorrhage |
| L11..12 | Antepartum bleeding |
| L110.00 | Placenta praevia without haemorrhage |
| L110000 | Placenta praevia without haemorrhage unspecified |
| L110100 | Placenta praevia without haemorrhage - delivered |
| L110200 | Placenta praevia without haemorrhage - not delivered |
| L110z00 | Placenta praevia without haemorrhage NOS |
| L111.00 | Placenta praevia with haemorrhage |
| L111000 | Placenta praevia with haemorrhage unspecified |
| L111100 | Placenta praevia with haemorrhage - delivered |
| L111200 | Placenta praevia with haemorrhage - not delivered |
| L111z00 | Placenta praevia with haemorrhage NOS |
| L112.00 | Placental abruption |
| L112.11 | Ablatio placentae |
| L112.12 | Couvelaire uterus |
| L112000 | Placental abruption unspecified |
| L112100 | Placental abruption - delivered |
| L112200 | Placental abruption - not delivered |
| L112300 | Premature separation of placenta with coagulation defect |
| L112z00 | Placental abruption NOS |
| L113.00 | Antepartum haemorrhage with coagulation defect |
| L113.11 | Antepartum haemorrhage with afibrinogenaemia |
| L113.12 | Antepartum haemorrhage with hyperfibrinolysis |
| L113.13 | Antepartum haemorrhage with hypofibrinogenaemia |
| L113000 | Antepartum haemorrhage with coagulation defect unspecified |
| L113100 | Antepartum haemorrhage with coagulation defect - delivered |
| L113200 | Antepartum haemorrhage with coagulation defect - not deliv |
| L113z00 | Antepartum haemorrhage with coagulation defect NOS |
| L114.00 | Antepartum haemorrhage with trauma |
| L114000 | Antepartum haemorrhage with trauma unspecified |
| L114100 | Antepartum haemorrhage with trauma - delivered |
| L114200 | Antepartum haemorrhage with trauma - not delivered |
| L114z00 | Antepartum haemorrhage with trauma NOS |
| L115.00 | Antepartum haemorrhage with uterine leiomyoma |
| L115.11 | Antepartum haemorrhage with fibroid |
| L115.12 | Antepartum haemorrhage with uterine fibroid |
| L115000 | Antepartum haemorrhage with uterine leiomyoma unspecified |
| L115100 | Antepartum haemorrhage with uterine leiomyoma - delivered |
| L115200 | Antepartum haemorrhage with uterine leiomyoma - not deliv |
| L115z00 | Antepartum haemorrhage with uterine leiomyoma NOS |
| L116.00 | Placenta praevia |
| L11y.00 | Other antepartum haemorrhage |
| L11y000 | Other antepartum haemorrhage unspecified |
| L11y100 | Other antepartum haemorrhage - delivered |
| L11y200 | Other antepartum haemorrhage - not delivered |
| L11yz00 | Other antepartum haemorrhage NOS |
| L11z.00 | Antepartum haemorrhage NOS |
| L11z000 | Antepartum haemorrhage NOS, unspecified |
| L11z100 | Antepartum haemorrhage NOS - delivered |
| L11z200 | Antepartum haemorrhage NOS - not deliv |
| L11zz00 | Antepartum haemorrhage NOS |
| L12..00 | Hypertension complicating pregnancy/childbirth/puerperium |
| L120.00 | Benign essential hypertension in pregnancy/childbirth/puerp |
| L120000 | Benign essential hypertension in preg/childb/puerp unspec |
| L120100 | Benign essential hypertension in preg/childb/puerp - deliv |
| L120200 | Benign ess hypert in preg/childb/puerp - deliv with p/n comp |
| L120300 | Benign essential hypertension in preg/childb/puerp-not deliv |
| L120400 | Benign essential hypertension in preg/childb/puerp +p/n comp |
| L120z00 | Benign essential hypertension in preg/childb/puerp NOS |
| L121.00 | Renal hypertension in pregnancy/childbirth/puerperium |
| L121000 | Renal hypertension in pregnancy/childbirth/puerp unspecified |
| L121100 | Renal hypertension in pregnancy/childbirth/puerp - delivered |
| L121200 | Renal hypertension in preg/childb/puerp -deliv with p/n comp |
| L121300 | Renal hypertension in preg/childbirth/puerp - not delivered |
| L121400 | Renal hypertension in preg/childb/puerp + p/n complication |
| L121z00 | Renal hypertension in pregnancy/childbirth/puerperium NOS |
| L122.00 | Other pre-existing hypertension in preg/childbirth/puerp |
| L122000 | Other pre-existing hypertension in preg/childb/puerp unspec |
| L122100 | Other pre-existing hypertension in preg/childb/puerp - deliv |
| L122200 | Oth pre-exist hypert in preg/childb/puerp -del with p/n comp |
| L122300 | Other pre-exist hypertension in preg/childb/puerp-not deliv |
| L122400 | Other pre-exist hypertension in preg/childb/puerp + p/n comp |
| L122z00 | Other pre-existing hypertension in preg/childb/puerp NOS |
| L123.00 | Transient hypertension of pregnancy |
| L123000 | Transient hypertension of pregnancy unspecified |
| L123100 | Transient hypertension of pregnancy - delivered |
| L123200 | Transient hypertension of pregnancy - deliv with p/n comp |
| L123300 | Transient hypertension of pregnancy - not delivered |
| L123400 | Transient hypertension of pregnancy + postnatal complication |
| L123500 | Gestational hypertension |
| L123600 | Transient hypertension of pregnancy |
| L123z00 | Transient hypertension of pregnancy NOS |
| L124.00 | Mild or unspecified pre-eclampsia |
| L124.11 | Mild pre-eclampsia |
| L124.12 | Toxaemia NOS |
| L124000 | Mild or unspecified pre-eclampsia unspecified |
| L124100 | Mild or unspecified pre-eclampsia - delivered |
| L124200 | Mild or unspecified pre-eclampsia - delivered with p/n comp |
| L124300 | Mild or unspecified pre-eclampsia - not delivered |
| L124400 | Mild or unspecified pre-eclampsia with p/n complication |
| L124500 | Mild pre-eclampsia |
| L124600 | Pre-eclampsia, unspecified |
| L124z00 | Mild or unspecified pre-eclampsia NOS |
| L125.00 | Severe pre-eclampsia |
| L125000 | Severe pre-eclampsia unspecified |
| L125100 | Severe pre-eclampsia - delivered |
| L125200 | Severe pre-eclampsia - delivered with postnatal complication |
| L125300 | Severe pre-eclampsia - not delivered |
| L125400 | Severe pre-eclampsia with postnatal complication |
| L125z00 | Severe pre-eclampsia NOS |
| L126.00 | Eclampsia |
| L126000 | Eclampsia unspecified |
| L126100 | Eclampsia - delivered |
| L126200 | Eclampsia - delivered with postnatal complication |
| L126300 | Eclampsia - not delivered |
| L126400 | Eclampsia with postnatal complication |
| L126500 | Eclampsia in pregnancy |
| L126600 | Eclampsia in labour |
| L126z00 | Eclampsia NOS |
| L127.00 | Pre-eclampsia or eclampsia with pre-existing hypertension |
| L127000 | Pre-eclampsia or eclampsia with hypertension unspecified |
| L127100 | Pre-eclampsia or eclampsia with hypertension - delivered |
| L127200 | Pre-eclampsia or eclampsia with hypertension - del+p/n comp |
| L127300 | Pre-eclampsia or eclampsia with hypertension - not delivered |
| L127400 | Pre-eclampsia or eclampsia with hypertension + p/n comp |
| L127z00 | Pre-eclampsia or eclampsia + pre-existing hypertension NOS |
| L128.00 | Pre-exist hypertension compl preg childbirth and puerperium |
| L128000 | Pre-exist hyperten heart dis compl preg childbth+puerperium |
| L128100 | Pre-exist hyperten heart renal dis comp preg chldbirth/puerp |
| L128200 | Pre-exist 2ndry hypertens comp preg childbth and puerperium |
| L129.00 | Moderate pre-eclampsia |
| L12A.00 | HELLP - Syndrome haemolysis, elev liver enzyme low platelets |
| L12B.00 | Proteinuric hypertension of pregnancy |
| L12z.00 | Unspecified hypertension in pregnancy/childbirth/puerperium |
| L12z000 | Unspecified hypertension in preg/childb/puerp unspecified |
| L12z100 | Unspecified hypertension in preg/childb/puerp - delivered |
| L12z200 | Unspecified hypertension in preg/childb/puerp -del +p/n comp |
| L12z300 | Unspecified hypertension in preg/childb/puerp - not deliv |
| L12z400 | Unspecified hypertension in preg/childb/puerp with p/n comp |
| L12zz00 | Unspecified hypertension in preg/childb/puerp NOS |
| L13..00 | Excessive pregnancy vomiting |
| L13..11 | Hyperemesis gravidarum |
| L13..12 | Hyperemesis of pregnancy |
| L130.00 | Mild hyperemesis gravidarum |
| L130.11 | Morning sickness |
| L130000 | Mild hyperemesis unspecified |
| L130100 | Mild hyperemesis-delivered |
| L130200 | Mild hyperemesis-not delivered |
| L130z00 | Mild hyperemesis gravidarum NOS |
| L131.00 | Hyperemesis gravidarum with metabolic disturbance |
| L131000 | Hyperemesis gravidarum with metabolic disturbance unsp |
| L131100 | Hyperemesis gravidarum with metabolic disturbance - deliv |
| L131200 | Hyperemesis gravidarum with metabolic disturbance - not del |
| L131z00 | Hyperemesis gravidarum with metabolic disturbance NOS |
| L132.00 | Late vomiting of pregnancy |
| L132000 | Late pregnancy vomiting unspecified |
| L132100 | Late pregnancy vomiting - delivered |
| L132200 | Late pregnancy vomiting - not delivered |
| L132z00 | Late pregnancy vomiting NOS |
| L13y.00 | Other pregnancy vomiting |
| L13y000 | Other pregnancy vomiting unspecified |
| L13y100 | Other pregnancy vomiting - delivered |
| L13y200 | Other pregnancy vomiting - not delivered |
| L13yz00 | Other pregnancy vomiting NOS |
| L13z.00 | Unspecified pregnancy vomiting |
| L13z000 | Unspecified pregnancy vomiting unspecified |
| L13z100 | Unspecified pregnancy vomiting - delivered |
| L13z200 | Unspecified pregnancy vomiting - not delivered |
| L13zz00 | Unspecified pregnancy vomiting NOS |
| L14..00 | Early or threatened labour |
| L14..11 | Premature labour |
| L140.00 | Threatened premature labour |
| L140.11 | False labour |
| L140000 | Threatened premature labour unspecified |
| L140100 | Threatened premature labour - not delivered |
| L140200 | False labour at or after 37 completed weeks of gestation |
| L140z00 | Threatened premature labour NOS |
| L141.00 | Other threatened labour |
| L141000 | Other threatened labour unspecified |
| L141100 | Other threatened labour - not delivered |
| L141z00 | Other threatened labour NOS |
| L142.00 | Early onset of delivery |
| L142.11 | Premature delivery |
| L142000 | Early onset of delivery unspecified |
| L142100 | Early onset of delivery - delivered |
| L142z00 | Early onset of delivery NOS |
| L143.00 | Premature labour and delivery |
| L143000 | Premature labour without delivery |
| L143100 | Premature labour with premature delivery |
| L143200 | Premature labour with term delivery |
| L143300 | Premature delivery without labour |
| L14z.00 | Early or threatened labour NOS |
| L15..00 | Prolonged or post-term pregnancy |
| L15..11 | Post-term pregnancy |
| L150.00 | Post-term pregnancy |
| L150000 | Post-term pregnancy unspecified |
| L150100 | Post-term pregnancy - delivered |
| L150200 | Post-term pregnancy - not delivered |
| L150z00 | Post-term pregnancy NOS |
| L15z.00 | Prolonged pregnancy NOS |
| L16..00 | Other pregnancy complication NEC |
| L160.00 | Papyraceous fetus |
| L160000 | Papyraceous fetus unspecified |
| L160100 | Papyraceous fetus - delivered |
| L160200 | Papyraceous fetus - not delivered |
| L160z00 | Papyraceous fetus NOS |
| L161.00 | Oedema or excessive weight gain in pregnancy no hypertension |
| L161.11 | Excessive weight gain in pregnancy |
| L161.12 | Maternal obesity syndrome |
| L161.13 | Gestational oedema |
| L161000 | Oedema or excessive weight gain in pregnancy, unspecified |
| L161100 | Oedema or excessive weight gain in pregnancy, delivered |
| L161200 | Oedema/excess weight gain preg - delivered + postnatal compl |
| L161300 | Oedema or excessive weight gain in pregnancy - not delivered |
| L161400 | Oedema/excessive weight gain in preg+postnatal complication |
| L161z00 | Oedema or excessive weight gain in pregnancy NOS |
| L162.00 | Unspecified renal disease in pregnancy |
| L162.11 | Albuminuria in pregnancy without hypertension |
| L162.12 | Nephropathy NOS in pregnancy without hypertension |
| L162.13 | Uraemia in pregnancy without hypertension |
| L162000 | Unspecified renal disease in pregnancy unspecified |
| L162100 | Unspecified renal disease in pregnancy - delivered |
| L162200 | Unspecified renal disease in pregnancy - del with p/n comp |
| L162300 | Unspecified renal disease in pregnancy - not delivered |
| L162400 | Unspecified renal disease in pregnancy with p/n complication |
| L162z00 | Unspecified renal disease in pregnancy NOS |
| L163.00 | Habitual aborter |
| L163000 | Habitual aborter - unspecified |
| L163100 | Habitual aborter - delivered |
| L163200 | Habitual aborter - not delivered |
| L163300 | Pregnancy care of habitual aborter |
| L163z00 | Habitual aborter NOS |
| L164.00 | Peripheral neuritis in pregnancy |
| L164000 | Peripheral neuritis in pregnancy unspecified |
| L164100 | Peripheral neuritis in pregnancy - delivered |
| L164200 | Peripheral neuritis in pregnancy - delivered with p/n comp |
| L164300 | Peripheral neuritis in pregnancy - not delivered |
| L164400 | Peripheral neuritis in pregnancy with postnatal complication |
| L164z00 | Peripheral neuritis in pregnancy NOS |
| L165.00 | Asymptomatic bacteriuria in pregnancy |
| L165000 | Asymptomatic bacteriuria in pregnancy unspecified |
| L165100 | Asymptomatic bacteriuria in pregnancy - delivered |
| L165200 | Asymptomatic bacteriuria in pregnancy - del with p/n comp |
| L165300 | Asymptomatic bacteriuria in pregnancy - not delivered |
| L165400 | Asymptomatic bacteriuria in pregnancy with postnatal comp |
| L165z00 | Asymptomatic bacteriuria in pregnancy NOS |
| L166.00 | Genitourinary tract infections in pregnancy |
| L166.11 | Cystitis of pregnancy |
| L166000 | Genitourinary tract infection in pregnancy unspecified |
| L166100 | Genitourinary tract infection in pregnancy - delivered |
| L166200 | Genitourinary tract infection in pregnancy - deliv +p/n comp |
| L166300 | Genitourinary tract infection in pregnancy - not delivered |
| L166400 | Genitourinary tract infection in pregnancy with p/n comp |
| L166500 | Infections of kidney in pregnancy |
| L166600 | Urinary tract infection following delivery |
| L166700 | Infections of the genital tract in pregnancy |
| L166800 | Urinary tract infection complicating pregnancy |
| L166z00 | Genitourinary tract infection in pregnancy NOS |
| L166z11 | UTI - urinary tract infection in pregnancy |
| L167.00 | Liver disorder in pregnancy |
| L167000 | Liver disorder in pregnancy unspecified |
| L167100 | Liver disorder in pregnancy - delivered |
| L167200 | Liver disorder in pregnancy - not delivered |
| L167z00 | Liver disorder in pregnancy NOS |
| L168.00 | Fatigue during pregnancy |
| L168000 | Fatigue during pregnancy unspecified |
| L168100 | Fatigue during pregnancy - delivered |
| L168200 | Fatigue during pregnancy - delivered with postnatal comp |
| L168300 | Fatigue during pregnancy - not delivered |
| L168400 | Fatigue during pregnancy with postnatal complication |
| L168z00 | Fatigue during pregnancy NOS |
| L169.00 | Herpes gestationis |
| L169000 | Herpes gestationis unspecified |
| L169100 | Herpes gestationis - delivered |
| L169200 | Herpes gestationis - delivered with postnatal complication |
| L169300 | Herpes gestationis - not delivered |
| L169400 | Herpes gestationis with postnatal complication |
| L169z00 | Herpes gestationis NOS |
| L16A.00 | Glycosuria during pregnancy |
| L16A000 | Glycosuria during pregnancy unspecified |
| L16A100 | Glycosuria during pregnancy - delivered |
| L16A200 | Glycosuria during pregnancy - delivered with p/n comp |
| L16A300 | Glycosuria during pregnancy - not delivered |
| L16A400 | Glycosuria during pregnancy with postnatal complication |
| L16Az00 | Glycosuria during pregnancy NOS |
| L16B.00 | Braxton-Hicks contractions |
| L16C.00 | Pregnancy induced oedema+proteinuria without hypertension |
| L16C000 | Gestational proteinuria |
| L16C100 | Gestational oedema with proteinuria |
| L16D.00 | Excessive weight gain in pregnancy |
| L16E.00 | Pregnancy pruritus |
| L16y.00 | Other pregnancy complications |
| L16y000 | Other pregnancy complication unspecified |
| L16y100 | Other pregnancy complication - delivered |
| L16y200 | Other pregnancy complication - delivered with postnatal comp |
| L16y300 | Other pregnancy complication - not delivered |
| L16y400 | Other pregnancy complication with postnatal complication |
| L16y500 | Abdominal pain in pregnancy |
| L16yz00 | Other pregnancy complication NOS |
| L16z.00 | Pregnancy complication NOS |
| L17..00 | Infective/parasitic disease in preg/childbirth/puerperium |
| L170.00 | Maternal syphilis in pregnancy/childbirth/puerperium |
| L170000 | Maternal syphilis, unspec whether in pregnancy or puerperium |
| L170100 | Maternal syphilis during pregnancy - baby delivered |
| L170200 | Maternal syphilis in puerperium - baby delivered |
| L170300 | Maternal syphilis during pregnancy - baby not yet delivered |
| L170400 | Maternal syphilis in puerperium - baby previously delivered |
| L170z00 | Maternal syphilis in pregnancy/childbirth/puerperium NOS |
| L171.00 | Maternal gonorrhoea during pregnancy/childbirth/puerperium |
| L171000 | Maternal gonorrhoea, unspec whether in pregnancy/puerperium |
| L171100 | Maternal gonorrhoea during pregnancy - baby delivered |
| L171200 | Maternal gonorrhoea in puerperium - baby delivered |
| L171300 | Maternal gonorrhoea in pregnancy - baby not yet delivered |
| L171400 | Maternal gonorrhoea in puerperium- baby previously delivered |
| L171z00 | Maternal gonorrhoea in pregnancy/childbirth/puerperium NOS |
| L172.00 | Other venereal diseases in pregnancy/childbirth/puerperium |
| L172000 | Other maternal venereal disease, unspec pregnancy/puerperium |
| L172100 | Other maternal venereal disease during pregnancy- baby deliv |
| L172200 | Other maternal venereal disease in puerperium-baby delivered |
| L172300 | Other maternal venereal dis. in pregnancy-baby not delivered |
| L172400 | Other mat. venereal dis. in puerperium-baby previously deliv |
| L172z00 | Other mat. venereal dis. in pregnancy/childbirth/puerp. NOS |
| L173.00 | Maternal tuberculosis in pregnancy/childbirth/puerperium |
| L173000 | Maternal tuberculosis,unspec whether in pregnancy/puerperium |
| L173100 | Maternal tuberculosis during pregnancy - baby delivered |
| L173200 | Maternal tuberculosis in puerperium - baby delivered |
| L173300 | Maternal tuberculosis in pregnancy - baby not yet delivered |
| L173400 | Maternal tuberculosis in puerperium - baby previously deliv. |
| L173z00 | Maternal tuberculosis in pregnancy/childbirth/puerperium NOS |
| L174.00 | Maternal malaria in pregnancy, childbirth and the puerperium |
| L174000 | Maternal malaria, unspec whether during pregnancy/puerperium |
| L174100 | Maternal malaria during pregnancy - baby delivered |
| L174200 | Maternal malaria in puerperium - baby delivered |
| L174300 | Maternal malaria during pregnancy - baby not yet delivered |
| L174400 | Maternal malaria in puerperium - baby previously delivered |
| L174z00 | Maternal malaria during pregnancy/childbirth/puerperium NOS |
| L175.00 | Maternal rubella in pregnancy, childbirth and the puerperium |
| L175.11 | Rubella contact in pregnancy |
| L175000 | Maternal rubella, unspecified whether pregnancy/puerperium |
| L175100 | Maternal rubella during pregnancy - baby delivered |
| L175200 | Maternal rubella in puerperium - baby delivered |
| L175300 | Maternal rubella during pregnancy - baby not yet delivered |
| L175400 | Maternal rubella in puerperium - baby previously delivered |
| L175z00 | Maternal rubella in pregnancy/childbirth/puerperium NOS |
| L176.00 | Other maternal viral dis. in pregnancy/childbirth/puerperium |
| L176000 | Other maternal viral disease, unspec in pregnancy/puerperium |
| L176100 | Other maternal viral disease in pregnancy - baby delivered |
| L176200 | Other maternal viral disease in puerperium - baby delivered |
| L176300 | Other maternal viral dis.in pregnancy-baby not yet delivered |
| L176400 | Other mat.viral dis. in puerperium-baby previously delivered |
| L176500 | Viral hepatitis comp pregnancy, childbirth & the puerperium |
| L176z00 | Other maternal viral dis. in pregnancy/childbirth/puerp. NOS |
| L177.00 | Infections of bladder in pregnancy |
| L178.00 | Infections of urethra in pregnancy |
| L179.00 | HIV disease complicating pregnancy childbirth puerperium |
| L17y.00 | Other mat.infective/parasitic disease in preg/childb/puerp. |
| L17y000 | Other mat. infective/parasitic disease in preg/puerp unspec |
| L17y100 | Other mat.infective/parasitic dis in pregnancy - delivered |
| L17y200 | Other mat.infect/parasit dis in puerperium - baby delivered |
| L17y300 | Other mat infective/parasit dis in pregnancy - not delivered |
| L17y400 | Other mat.infective/parasit dis in puerp-baby previously del |
| L17yz00 | Other mat.infective/parasitic dis in preg/childb/puerp NOS |
| L17z.00 | Maternal infect/parasitic dis NOS in pregnancy/childb/puerp |
| L17z000 | Mat infect/parasitic dis NOS - pregnancy/puerperium unspec |
| L17z100 | Mat infect/parasitic dis NOS in pregnancy - baby delivered |
| L17z200 | Mat infect/parasitic dis NOS in puerperium - baby delivered |
| L17z300 | Mat infect/parasitic dis NOS in pregnancy-baby not delivered |
| L17z400 | Mat infect/parasitic dis NOS in puerp-baby previously deliv |
| L17zz00 | Mat infect/parasitic dis NOS in preg/childbirth/puerp NOS |
| L18..00 | Other medical condition in pregnancy/childbirth/puerperium |
| L180.00 | Diabetes mellitus during pregnancy/childbirth/puerperium |
| L180000 | Diabetes mellitus - unspec whether in pregnancy/puerperium |
| L180100 | Diabetes mellitus during pregnancy - baby delivered |
| L180200 | Diabetes mellitus in puerperium - baby delivered |
| L180300 | Diabetes mellitus during pregnancy - baby not yet delivered |
| L180400 | Diabetes mellitus in pueperium - baby previously delivered |
| L180500 | Pre-existing diabetes mellitus, insulin-dependent |
| L180600 | Pre-existing diabetes mellitus, non-insulin-dependent |
| L180700 | Pre-existing malnutrition-related diabetes mellitus |
| L180800 | Diabetes mellitus arising in pregnancy |
| L180811 | Gestational diabetes mellitus |
| L180900 | Gestational diabetes mellitus |
| L180A00 | Pre-exst typ 1 diab mll in prg |
| L180B00 | Pre-exst typ 2 diab mll in prg |
| L180X00 | Pre-existing diabetes mellitus, unspecified |
| L180z00 | Diabetes mellitus in pregnancy/childbirth/puerperium NOS |
| L181.00 | Thyroid dysfunction in pregnancy/childbirth/puerperium |
| L181000 | Thyroid dysfunction - unspec whether in pregnancy/puerperium |
| L181100 | Thyroid dysfunction during pregnancy - baby delivered |
| L181200 | Thyroid dysfunction in puerperium - baby delivered |
| L181300 | Thyroid dysfunction in pregnancy - baby not yet delivered |
| L181400 | Thyroid dysfunction in puerperium- baby previously delivered |
| L181500 | Postpartum thyroiditis |
| L181z00 | Thyroid dysfunction in pregnancy/childbirth/puerperium NOS |
| L182.00 | Anaemia during pregnancy, childbirth and the puerperium |
| L182000 | Anaemia - unspecified whether in pregnancy or the puerperium |
| L182100 | Anaemia during pregnancy - baby delivered |
| L182200 | Anaemia in the puerperium - baby delivered |
| L182300 | Anaemia during pregnancy - baby not yet delivered |
| L182400 | Anaemia in the puerperium - baby previously delivered |
| L182500 | Iron deficiency anaemia of pregnancy |
| L182z00 | Anaemia during pregnancy/childbirth/puerperium NOS |
| L183.00 | Drug dependence in pregnancy, childbirth and the puerperium |
| L183.11 | Pregnancy and drug dependence |
| L183000 | Drug dependence - unspec whether during pregnancy/puerperium |
| L183100 | Drug dependence during pregnancy - baby delivered |
| L183200 | Drug dependence in the puerperium - baby delivered |
| L183300 | Drug dependence during pregnancy - baby not yet delivered |
| L183400 | Drug dependence in puerperium - baby previously delivered |
| L183z00 | Drug dependence during pregnancy/childbirth/puerperium NOS |
| L184.00 | Mental disorders in pregnancy, childbirth and the puerperium |
| L184000 | Mental disorder - unspec whether in pregnancy/puerperium |
| L184100 | Mental disorder during pregnancy - baby delivered |
| L184200 | Mental disorder in the puerperium - baby delivered |
| L184300 | Mental disorder during pregnancy - baby not yet delivered |
| L184400 | Mental disorder in puerperium - baby previously delivered |
| L184z00 | Mental disorder during pregnancy/childbirth/puerperium NOS |
| L185.00 | Congenital cardiovascular disorders in preg/childb/puerp |
| L185.11 | Congenital heart disease in pregnancy |
| L185000 | Congenital cardiovasc dis - unsp whether in preg/puerperium |
| L185100 | Congenital cardiovasc dis in pregnancy - baby delivered |
| L185200 | Congenital cardiovasc dis in puerp - baby delivered |
| L185300 | Congenital cardiovasc dis in pregnancy - baby not delivered |
| L185400 | Congenital cardiovasc dis in puerp - baby previously deliv |
| L185z00 | Congenital cardiovascular disorder in preg/childb/puerp NOS |
| L186.00 | Other cardiovascular diseases in pregnancy/childbirth/puerp |
| L186.11 | Heart disease during pregnancy |
| L186000 | Other cardiovascular dis - unsp whether in preg/puerperium |
| L186100 | Other cardiovascular disease in pregnancy - baby delivered |
| L186200 | Other cardiovasc dis in puerperium - baby delivered |
| L186300 | Other cardiovascular dis in pregnancy - baby not delivered |
| L186400 | Other cardiovasc dis in puerp - baby previously delivered |
| L186500 | Cardiomyopathy in the puerperium |
| L186z00 | Other cardiovascular disease in pregnancy/childb/puerp NOS |
| L187.00 | Orthopaedic disorders in pregnancy/childbirth/puerperium |
| L187000 | Orthopaedic disorder - unsp whether in pregnancy/puerperium |
| L187100 | Orthopaedic disorder during pregnancy - baby delivered |
| L187200 | Orthopaedic disorder in puerperium - baby delivered |
| L187300 | Orthopaedic disorder in pregnancy - baby not yet delivered |
| L187400 | Orthopaedic disorder in puerperium-baby previously delivered |
| L187z00 | Orthopaedic disorder in pregnancy/childbirth/puerperium NOS |
| L188.00 | Abnormal glucose tolerance test in pregnancy/childb/puerp |
| L188.11 | GTT - glucose tolerance test abnormal in preg/childb/puerp |
| L188000 | Abnormal GTT - unspec whether during pregnancy/puerperium |
| L188100 | Abnormal GTT during pregnancy - baby delivered |
| L188200 | Abnormal GTT in puerperium - baby delivered |
| L188300 | Abnormal GTT during pregnancy - baby not yet delivered |
| L188400 | Abnormal GTT in puerperium - baby previously delivered |
| L188z00 | Abnormal GTT in pregnancy/childbirth/puerperium NOS |
| L189.00 | Dis resp syst comp pregnancy, childbirth & puerperium |
| L18A.00 | Dis of the digestive sys comp preg childbirth and puerp |
| L18A000 | Cholestasis of pregnancy |
| L18B.00 | Dis of the skin and subcut tis comp preg childbrth puerp |
| L18C.00 | Endocrine nutrition+metab dis complic pregn,childbirth+puerp |
| L18D.00 | Dis nervous syst complic pregnancy,childbirth and puerperium |
| L18z.00 | Medical condition NOS in pregnancy/childbirth/puerperium |
| L18z000 | Medical condition NOS - unsp whether in pregnancy/puerperium |
| L18z100 | Medical condition NOS during pregnancy - baby delivered |
| L18z200 | Medical condition NOS in puerperium - baby delivered |
| L18z300 | Medical condition NOS in pregnancy - baby not yet delivered |
| L18z400 | Medical condition NOS in puerperium - baby previously deliv |
| L18zz00 | Medical condition NOS in pregnancy/childb/puerp NOS |
| L19..00 | Complications specific to multiple gestation |
| L191.00 | Continuing pregnancy after abortion of one fetus or more |
| L192.00 | Continuing preg after intrauterine death one fetus or more |
| L1A..00 | Sublux of symphysis pubis in preg childbirth and puerp |
| L1y..00 | Complications of pregnancy/childbirth/puerperium OS |
| L1z..00 | Complications of pregnancy/childbirth/puerperium NOS |
| L2...00 | Risk factors in pregnancy |
| L20..00 | Normal delivery in a completely normal case |
| L20..11 | Spontaneous vaginal delivery |
| L200.00 | Normal delivery but ante- or post- natal conditions present |
| L20z.00 | Normal delivery in completely normal case NOS |
| L21..00 | Multiple pregnancy |
| L21..11 | Gestation - multiple |
| L210.00 | Twin pregnancy |
| L210000 | Twin pregnancy unspecified |
| L210100 | Twin pregnancy - delivered |
| L210200 | Twin pregnancy with antenatal problem |
| L210z00 | Twin pregnancy NOS |
| L211.00 | Triplet pregnancy |
| L211000 | Triplet pregnancy unspecified |
| L211100 | Triplet pregnancy - delivered |
| L211200 | Triplet pregnancy with antenatal problem |
| L211z00 | Triplet pregnancy NOS |
| L212.00 | Quadruplet pregnancy |
| L212000 | Quadruplet pregnancy unspecified |
| L212100 | Quadruplet pregnancy - delivered |
| L212200 | Quadruplet pregnancy with antenatal problem |
| L212z00 | Quadruplet pregnancy NOS |
| L213.00 | Multiple delivery |
| L213000 | Multiple delivery, all spontaneous |
| L213100 | Multiple delivery, all by forceps and vacuum extractor |
| L213200 | Multiple delivery, all by caesarean section |
| L21y.00 | Other multiple pregnancy |
| L21y000 | Other multiple pregnancy unspecified |
| L21y100 | Other multiple pregnancy - delivered |
| L21y200 | Other multiple pregnancy with antenatal problem |
| L21yz00 | Other multiple pregnancy NOS |
| L21z.00 | Multiple pregnancy NOS |
| L21z000 | Multiple pregnancy NOS, unspecified |
| L21z100 | Multiple pregnancy NOS - delivered |
| L21z200 | Multiple pregnancy NOS with antenatal problem |
| L21zz00 | Multiple pregnancy NOS |
| L22..00 | Malposition and malpresentation of fetus |
| L22..11 | Malpresentation of fetus |
| L220.00 | Fetus - unstable lie |
| L220000 | Unstable lie unspecified |
| L220100 | Unstable lie - delivered |
| L220200 | Unstable lie with antenatal problem |
| L220z00 | Unstable lie NOS |
| L221.00 | Cephalic version NOS |
| L221000 | Cephalic version NOS, unspecified |
| L221100 | Cephalic version NOS - delivered |
| L221200 | Cephalic version NOS with antenatal problem |
| L221z00 | Cephalic version NOS |
| L222.00 | Breech presentation |
| L222.11 | Assisted breech delivery |
| L222.12 | Breech delivery |
| L222.13 | Spontaneous breech delivery |
| L222000 | Breech presentation unspecified |
| L222100 | Breech presentation - delivered |
| L222200 | Breech presentation with antenatal problem |
| L222z00 | Breech presentation NOS |
| L223.00 | Oblique presentation |
| L223000 | Oblique lie unspecified |
| L223100 | Oblique lie - delivered |
| L223200 | Oblique lie with antenatal problem |
| L223z00 | Oblique lie NOS |
| L224.00 | Transverse presentation |
| L224000 | Transverse lie unspecified |
| L224100 | Transverse lie - delivered |
| L224200 | Transverse lie with antenatal problem |
| L224z00 | Transverse lie NOS |
| L224z11 | Shoulder presentation |
| L225.00 | Face presentation |
| L225000 | Face presentation unspecified |
| L225100 | Face presentation - delivered |
| L225200 | Face presentation with antenatal problem |
| L225z00 | Face presentation NOS |
| L226.00 | Brow presentation |
| L226000 | Brow presentation unspecified |
| L226100 | Brow presentation - delivered |
| L226200 | Brow presentation with antenatal problem |
| L226z00 | Brow presentation NOS |
| L226z11 | Mentum presentation |
| L227.00 | High head at term |
| L227000 | High head at term unspecified |
| L227100 | High head at term - delivered |
| L227200 | High head at term with antenatal problem |
| L227z00 | High head at term NOS |
| L228.00 | Multiple pregnancy with malpresentation |
| L228000 | Multiple pregnancy with malpresentation unspecified |
| L228100 | Multiple pregnancy with malpresentation - delivered |
| L228200 | Multiple pregnancy with malpresentation with antenatal prob |
| L228z00 | Multiple pregnancy with malpresentation NOS |
| L229.00 | Prolapsed arm presentation |
| L229000 | Prolapsed arm unspecified |
| L229100 | Prolapsed arm - delivered |
| L229200 | Prolapsed arm with antenatal problem |
| L229z00 | Prolapsed arm NOS |
| L22y.00 | Other fetal malposition and malpresentation |
| L22y.11 | Compound presentation |
| L22y000 | Other fetal malposition and malpresentation unspecified |
| L22y100 | Other fetal malposition and malpresentation - delivered |
| L22y200 | Other fetal malposition and malpresentation with a/n prob |
| L22yz00 | Other fetal malposition and malpresentation NOS |
| L22z.00 | Fetal malposition and malpresentation NOS |
| L22z000 | Fetal malposition and malpresentation NOS, unspecified |
| L22z100 | Fetal malposition and malpresentation NOS - delivered |
| L22z200 | Fetal malposition and malpresentation NOS with a/n problem |
| L22zz00 | Fetal malposition and malpresentation NOS |
| L23..00 | Cephalo-pelvic disproportion |
| L230.00 | Disproportion - major pelvic abnormality |
| L230000 | Disproportion - major pelvic abnormality unspecified |
| L230100 | Disproportion - major pelvic abnormality - delivered |
| L230200 | Disproportion - major pelvic abnormality with antenatal prob |
| L230z00 | Disproportion - major pelvic abnormality NOS |
| L231.00 | Generally contracted pelvis |
| L231000 | Generally contracted pelvis unspecified |
| L231100 | Generally contracted pelvis - delivered |
| L231200 | Generally contracted pelvis with antenatal problem |
| L231z00 | Generally contracted pelvis NOS |
| L232.00 | Inlet pelvic contraction |
| L232000 | Inlet pelvic contraction unspecified |
| L232100 | Inlet pelvic contraction - delivered |
| L232200 | Inlet pelvic contraction with antenatal problem |
| L232z00 | Inlet pelvic contraction NOS |
| L233.00 | Outlet pelvic contraction |
| L233000 | Outlet pelvic contraction unspecified |
| L233100 | Outlet pelvic contraction - delivered |
| L233200 | Outlet pelvic contraction with antenatal problem |
| L233z00 | Outlet pelvic contraction NOS |
| L234.00 | Mixed feto-pelvic disproportion |
| L234000 | Mixed feto-pelvic disproportion unspecified |
| L234100 | Mixed feto-pelvic disproportion - delivered |
| L234200 | Mixed feto-pelvic disproportion with antenatal problem |
| L234z00 | Mixed feto-pelvic disproportion NOS |
| L235.00 | Large fetus causing disproportion |
| L235000 | Large fetus causing disproportion unspecified |
| L235100 | Large fetus causing disproportion - delivered |
| L235200 | Large fetus causing disproportion with antenatal problem |
| L235z00 | Large fetus causing disproportion NOS |
| L236.00 | Hydrocephalic disproportion |
| L236000 | Hydrocephalic disproportion unspecified |
| L236100 | Hydrocephalic disproportion - delivered |
| L236200 | Hydrocephalic disproportion with antenatal problem |
| L236z00 | Hydrocephalic disproportion NOS |
| L237.00 | Other fetal abnormality causing disproportion |
| L237.11 | Conjoined twins causing disproportion |
| L237000 | Other fetal abnormality causing disproportion unspecified |
| L237100 | Other fetal abnormality causing disproportion - delivered |
| L237200 | Other fetal abnormality causing disproportion with a/n prob |
| L237z00 | Other fetal abnormality causing disproportion NOS |
| L23y.00 | Other disproportion |
| L23y000 | Other disproportion unspecified |
| L23y100 | Other disproportion - delivered |
| L23y200 | Other disproportion with antenatal problem |
| L23yz00 | Other disproportion NOS |
| L23z.00 | Disproportion NOS |
| L23z000 | Disproportion NOS, unspecified |
| L23z100 | Disproportion NOS - delivered |
| L23z200 | Disproportion NOS with antenatal problem |
| L23zz00 | Disproportion NOS |
| L24..00 | Pelvic soft tissue abnormality in pregnancy/childbirth/puerp |
| L240.00 | Congenital abnormality of uterus in preg/childbirth/puerp |
| L240.11 | Bicornuate uterus in pregnancy, childbirth and puerperium |
| L240.12 | Double uterus in pregnancy, childbirth and the puerperium |
| L240000 | Congenital abnormality of uterus affecting obstetric care |
| L240011 | Bicornuate uterus affecting obstetric care |
| L240100 | Congenital abnormality of uterus - baby delivered |
| L240111 | Bicornuate uterus - baby delivered |
| L240200 | Cong abnormality uterus - baby delivered + postpartum compl |
| L240211 | Bicornuate uterus - baby delivered + postpartum complication |
| L240300 | Cong abnorm uterus complicating a/n care, baby not delivered |
| L240311 | Bicornuate uterus complicating a/n care, baby not delivered |
| L240400 | Cong abnorm uterus complic p/n care - baby previously deliv |
| L240411 | Bicornuate uterus complic p/n care - baby previously deliv |
| L240z00 | Congenital abnormality uterus in pregnancy/childb/puerp NOS |
| L240z11 | Bicornuate uterus in pregnancy, childbirth or puerperium NOS |
| L241.00 | Tumour of uterine body in pregnancy/childbirth/puerperium |
| L241.11 | Uterine fibroids in pregnancy, childbirth and the puerperium |
| L241000 | Tumour of uterine body affecting obstetric care |
| L241011 | Uterine fibroid affecting obstetric care |
| L241100 | Tumour of uterine body - baby delivered |
| L241111 | Uterine fibroid - baby delivered |
| L241200 | Tumour of uterine body - baby delivered + p/n complication |
| L241211 | Uterine fibroid - baby delivered + postpartum complication |
| L241300 | Tumour of uterine body complicating a/n care, baby not deliv |
| L241311 | Uterine fibroid complicating a/n care, baby not delivered |
| L241400 | Tumour of uterine body complic p/n care, baby prev delivered |
| L241411 | Uterine fibroid complicating p/n care - baby delivered prev |
| L241z00 | Uterine body tumour in pregnancy/childbirth/puerperium NOS |
| L241z11 | Uterine fibroid in pregnancy/childbirth/puerperium NOS |
| L242.00 | Uterine scar from previous surgery in pregnancy/childb/puerp |
| L242000 | Uterine operation scar in pregnancy/childbirth/puerp unspec |
| L242100 | Uterine operation scar in pregnancy/childbirth/puerp - deliv |
| L242200 | Uterine operation scar in pregnancy/childb/puerp + a/n prob |
| L242z00 | Uterine operation scar in pregnancy/childbirth/puerp NOS |
| L243.00 | Retroverted incarcerated gravid uterus |
| L243000 | Retroverted incarcerated gravid uterus unspecified |
| L243100 | Retroverted incarcerated gravid uterus - delivered |
| L243200 | Retroverted incarcerated gravid uterus - delivered +p/n comp |
| L243300 | Retroverted incarcerated gravid uterus with antenatal prob |
| L243400 | Retroverted incarcerated gravid uterus with postnatal comp |
| L243z00 | Retroverted incarcerated gravid uterus NOS |
| L244.00 | Other uterine/pelvic floor abnormality in preg/childb/puerp |
| L244.11 | Cystocele in pregnancy, childbirth and the puerperium |
| L244.12 | Pendulous abdomen in pregnancy,childbirth and the puerperium |
| L244.13 | Rectocele in pregnancy, childbirth and the puerperium |
| L244000 | Other uterine/pelvic floor abnormal affecting obstetric care |
| L244011 | Cystocele affecting obstetric care |
| L244012 | Rectocele affecting obstetric care |
| L244100 | Other uterine/pelvic floor abnormality - baby delivered |
| L244111 | Cystocele - baby delivered |
| L244112 | Rectocele - baby delivered |
| L244200 | Other uterine/pelvic floor abn - delivered+postpartum compl |
| L244211 | Cystocele - delivered with postpartum complication |
| L244212 | Rectocele - delivered with postpartum complication |
| L244300 | Other uterine/pelvic floor abnormal - baby not yet delivered |
| L244311 | Cystocele complicating antenatal care - baby not delivered |
| L244312 | Rectocele complicating antenatal care - baby not delivered |
| L244400 | Other uterine/pelvic floor abn - baby delivered previously |
| L244411 | Cystocele complicating postpartum care - baby delivered prev |
| L244412 | Rectocele complicating postpartum care - baby delivered prev |
| L244z00 | Other uterine/pelvic floor abn in preg/childb/puerp NOS |
| L244z11 | Cystocele in pregnancy, childbirth or the puerperium NOS |
| L244z12 | Rectocele in pregnancy, childbirth or the puerperium NOS |
| L245.00 | Cervical incompetence |
| L245.11 | Shirodkar suture present |
| L245000 | Cervical incompetence unspecified |
| L245100 | Cervical incompetence - delivered |
| L245200 | Cervical incompetence - delivered with postnatal comp |
| L245300 | Cervical incompetence with antenatal problem |
| L245400 | Cervical incompetence with postnatal complication |
| L245z00 | Cervical incompetence NOS |
| L246.00 | Other cervical abnormality in pregnancy/childbirth/puerp |
| L246.11 | Polyp of cervix in pregnancy, childbirth and the puerperium |
| L246.12 | Stenosis of cervix in pregnancy, childbirth, puerperium |
| L246000 | Other cervical abnormality affecting obstetric care |
| L246100 | Other cervical abnormality - baby delivered |
| L246200 | Other cervical abnormality - baby delivered+postpartum compl |
| L246211 | Polyp of cervix - baby delivered+postpartum complication |
| L246212 | Stenosis of cervix - baby delivered+postpartum complication |
| L246300 | Other cervical abn complicating a/n care- baby not delivered |
| L246311 | Polyp of cervix complicating a/n care- baby not delivered |
| L246312 | Stenosis of cervix complicating a/n care- baby not delivered |
| L246400 | Other cervical abn complicating p/n care - baby deliv prev |
| L246411 | Polyp of cervix complicating p/n care - baby deliv prev |
| L246412 | Stenosis of cervix complicating p/n care - baby deliv prev |
| L246z00 | Other cervical abnormality in pregnancy/childbirth/puerp NOS |
| L246z11 | Polyp of cervix in pregnancy/childbirth/puerperium NOS |
| L246z12 | Stenosis of cervix in pregnancy/childbirth/puerperium NOS |
| L247.00 | Congenital/acquired abnormality vagina in preg/childb/puerp |
| L247.11 | Septate vagina in pregnancy, childbirth and the puerperium |
| L247.12 | Stenosis of vagina in pregnancy/childbirth/puerperium |
| L247.13 | Vaginal abnormality in pregnancy/childbirth/puerperium |
| L247000 | Vaginal abnormality affecting obstetric care |
| L247011 | Septate vagina affecting obstetric care |
| L247012 | Stenosis of vagina affecting obstetric care |
| L247100 | Vaginal abnormality - baby delivered |
| L247111 | Septate vagina - baby delivered |
| L247112 | Stenosis of vagina - baby delivered |
| L247200 | Vaginal abnormality - baby delivered+postpartum complication |
| L247211 | Septate vagina - baby delivered with postpartum complication |
| L247212 | Stenosis of vagina - baby delivered+postpartum complication |
| L247300 | Vaginal abnormality complicating a/n care-baby not delivered |
| L247311 | Septate vagina complicating a/n care- baby not yet delivered |
| L247312 | Stenosis of vagina complicating a/n care- baby not delivered |
| L247400 | Vaginal abnormality complicating p/n care - baby deliv prev |
| L247411 | Septate vagina complicating p/n care - baby delivered prev |
| L247412 | Stenosis of vagina complicating p/n care - baby deliv prev |
| L247z00 | Vaginal abnormality in pregnancy/childbirth/puerperium NOS |
| L247z11 | Septate vagina in pregnancy/childbirth/puerperium NOS |
| L247z12 | Stenosis of vagina in pregnancy/childbirth/puerperium NOS |
| L248.00 | Congenital/acquired abnormality vulva in preg/childb/puerp |
| L248.11 | Persistent hymen in pregnancy, childbirth and the puerperium |
| L248.12 | Rigid perineum in pregnancy, childbirth and the puerperium |
| L248.13 | Vulval abnormality in pregnancy/childbirth/puerperium |
| L248000 | Vulval abnormality affecting obstetric care |
| L248011 | Persistent hymen affecting obstetric care |
| L248012 | Rigid perineum affecting obstetric care |
| L248100 | Vulval abnormality - baby delivered |
| L248111 | Persistent hymen - baby delivered |
| L248112 | Rigid perineum - baby delivered |
| L248200 | Vulval abnormality - baby delivered+postpartum complication |
| L248211 | Persistent hymen - baby delivered+postpartum complication |
| L248212 | Rigid perineum - baby delivered with postpartum complication |
| L248300 | Vulval abn complicating a/n care - baby not yet delivered |
| L248311 | Persistent hymen complicating a/n care - baby not delivered |
| L248312 | Rigid perineum complicating a/n care - baby not delivered |
| L248400 | Vulval abn complicating p/n care - baby delivered previously |
| L248411 | Persistent hymen complicating p/n care - baby delivered prev |
| L248412 | Rigid perineum complicating p/n care - baby delivered prev |
| L248z00 | Vulval abnormality in pregnancy/childbirth/puerperium NOS |
| L248z11 | Persistent hymen in pregnancy/childbirth/puerperium NOS |
| L248z12 | Rigid perineum in pregnancy/childbirth/puerperium NOS |
| L24z.00 | Pelvic soft tissue abnormality in pregnancy/childbirth/puerp |
| L24z000 | Pelvic soft tissue abnormality in preg/childb/puerp unspec |
| L24z100 | Pelvic soft tissue abnormality in preg/childb/puerp - deliv |
| L24z200 | Pelvic soft tissue abnorm in preg/childb/puerp -del+p/n comp |
| L24z300 | Pelvic soft tissue abnorm in preg/childb/puerp with a/n prob |
| L24z400 | Pelvic soft tissue abnorm in preg/childb/puerp with p/n comp |
| L24zz00 | Pelvic soft tissue abnormality in preg/childb/puerp NOS |
| L25..00 | Known or suspected fetal abnormality |
| L250.00 | Fetus with central nervous system malformation |
| L250.11 | Suspect fetal anencephaly |
| L250.12 | Suspect fetal hydrocephaly |
| L250.13 | Suspect fetal spina bifida |
| L250000 | Fetus with central nervous system malformation unspecified |
| L250100 | Fetus with central nervous system malformation - delivered |
| L250200 | Fetus with central nervous system malformation + a/n problem |
| L250300 | Maternal care for suspected CNS malformation in fetus |
| L250400 | Maternal care for CNS malformation in fetus |
| L250z00 | Fetus with central nervous system malformation NOS |
| L251.00 | Fetus with chromosomal abnormality |
| L251.11 | Suspect cystic fibrosis fetus |
| L251.12 | Suspect mongol fetus |
| L251000 | Fetus with chromosomal abnormality unspecified |
| L251100 | Fetus with chromosomal abnormality - delivered |
| L251200 | Fetus with chromosomal abnormality with antenatal problem |
| L251300 | Maternal care for suspected chromosomal abnormality in fetus |
| L251400 | Maternal care for chromosomal abnormality in fetus |
| L251z00 | Fetus with chromosomal abnormality NOS |
| L252.00 | Fetus with hereditary disease |
| L252000 | Fetus with hereditary disease unspecified |
| L252100 | Fetus with hereditary disease - delivered |
| L252200 | Fetus with hereditary disease with antenatal problem |
| L252z00 | Fetus with hereditary disease NOS |
| L253.00 | Fetus with viral damage via mother |
| L253.11 | Fetus with suspected rubella damage via mother |
| L253000 | Fetus with viral damage via mother unspecified |
| L253100 | Fetus with viral damage via mother - delivered |
| L253200 | Fetus with viral damage via mother with antenatal problem |
| L253300 | Maternal care for damage to fetus from maternal rubella |
| L253z00 | Fetus with viral damage via mother NOS |
| L254.00 | Fetus with damage due to other maternal disease |
| L254.11 | Suspect fetal damage from maternal alcohol |
| L254.12 | Suspect fetal damage from maternal toxoplasmosis |
| L254000 | Fetus with damage due to other maternal disease unspecified |
| L254100 | Fetus with damage due to other maternal disease - delivered |
| L254200 | Fetus with damage due to other maternal disease + a/n prob |
| L254z00 | Fetus with damage due to other maternal disease NOS |
| L255.00 | Fetus with drug damage |
| L255000 | Fetus with drug damage unspecified |
| L255100 | Fetus with drug damage - delivered |
| L255200 | Fetus with drug damage with antenatal problem |
| L255300 | Maternal care for (suspected) damage to fetus from alcohol |
| L255z00 | Fetus with drug damage NOS |
| L256.00 | Fetus with radiation damage |
| L256000 | Fetus with radiation damage unspecified |
| L256100 | Fetus with radiation damage - delivered |
| L256200 | Fetus with radiation damage with antenatal problem |
| L256z00 | Fetus with radiation damage NOS |
| L257.00 | Fetus with damage due to intra-uterine contraceptive device |
| L257.11 | Fetus with damage due to coil |
| L257.12 | Fetus with damage due to intra-uterine contraceptive device |
| L257000 | Fetus with damage due to IUCD unspecified |
| L257100 | Fetus with damage due to IUCD - delivered |
| L257200 | Fetus with damage due to IUCD with antenatal problem |
| L257z00 | Fetus with damage due to IUCD NOS |
| L258.00 | Fetus with cardiovascular abnormality |
| L25y.00 | Fetus with other damage NEC |
| L25y000 | Fetus with other damage NEC, unspecified |
| L25y100 | Fetus with other damage NEC - delivered |
| L25y200 | Fetus with other damage NEC with antenatal problem |
| L25yz00 | Fetus with other damage NEC NOS |
| L25z.00 | Fetus with damage NOS |
| L25z000 | Fetus with damage NOS, unspecified |
| L25z100 | Fetus with damage NOS - delivered |
| L25z200 | Fetus with damage NOS with antenatal problem |
| L25z300 | Maternal care for suspect fetal abnormal and damage, unspec |
| L25z400 | Maternal care for fetal abnormality and damage, unspecified |
| L25zz00 | Fetus with damage NOS |
| L26..00 | Other fetal and placental problems |
| L260.00 | Fetal-maternal haemorrhage |
| L260000 | Fetal-maternal haemorrhage unspecified |
| L260100 | Fetal-maternal haemorrhage - delivered |
| L260200 | Fetal-maternal haemorrhage with antenatal problem |
| L260z00 | Fetal-maternal haemorrhage NOS |
| L261.00 | Rhesus isoimmunisation |
| L261.11 | Anti-D antibodies |
| L261000 | Rhesus isoimmunisation unspecified |
| L261100 | Rhesus isoimmunisation - delivered |
| L261200 | Rhesus isoimmunisation with antenatal problem |
| L261z00 | Rhesus isoimmunisation NOS |
| L262.00 | Other blood-group isoimmunisation |
| L262.11 | Other blood-group isoimmunisation |
| L262000 | Other blood-group isoimmunisation unspecified |
| L262100 | Other blood-group isoimmunisation - delivered |
| L262200 | Other blood-group isoimmunisation with antenatal problem |
| L262z00 | Other blood-group isoimmunisation NOS |
| L263.00 | Fetal distress - affecting management |
| L263.11 | Fetal acidosis |
| L263.12 | Fetal bradycardia |
| L263.13 | Fetal tachycardia |
| L263.14 | Meconium stained liquor |
| L263000 | Fetal distress unspecified |
| L263100 | Fetal distress - delivered |
| L263200 | Fetal distress with antenatal problem |
| L263300 | Labour and delivery complicated by fetal heart rate anomaly |
| L263311 | Maternal care for fetal hypoxia |
| L263400 | Labour and delivery complic by meconium in amniotic fluid |
| L263500 | Lab+del comp fetal ht rate anom wth meconium in amnio fluid |
| L263600 | Labour+delivery complicatd by biochem evidence/fetal stress |
| L263700 | Maternal care for fetal hypoxia |
| L263800 | Maternal care for fetal decelerations during pregnancy |
| L263900 | Maternal care for fetal tachycardia during pregnancy |
| L263A00 | Maternal care for fetal bradycardia during pregnancy |
| L263A11 | Maternal care for reduced fetal heart rate during pregnancy |
| L263B00 | Maternal care for fetal acidosis during pregnancy |
| L263z00 | Fetal distress NOS |
| L264.00 | Intrauterine death |
| L264.11 | Fetal death in utero |
| L264000 | Intrauterine death unspecified |
| L264100 | Intrauterine death - delivered |
| L264200 | Intrauterine death with antenatal problem |
| L264z00 | Intrauterine death NOS |
| L265.00 | Small-for-dates fetus in pregnancy |
| L265.11 | Placental insufficiency |
| L265000 | Small-for-dates unspecified |
| L265100 | Small-for-dates - delivered |
| L265200 | Small-for-dates with antenatal problem |
| L265300 | Maternal care for poor fetal growth |
| L265311 | Maternal care for intrauterine growth retardation |
| L265z00 | Small-for-dates NOS |
| L266.00 | Large-for-dates fetus in pregnancy |
| L266000 | Large-for-dates unspecified |
| L266100 | Large-for-dates - delivered |
| L266200 | Large-for-dates with antenatal problem |
| L266300 | Suspected macroscopic fetus |
| L266z00 | Large-for-dates NOS |
| L267.00 | Other placental conditions |
| L267.11 | Placental infarct |
| L267000 | Other placental conditions unspecified |
| L267100 | Other placental conditions - delivered |
| L267200 | Other placental conditions with antenatal problem |
| L267300 | Placental transfusion syndromes |
| L267400 | Malformation of placenta |
| L267500 | Other fetal problems |
| L267600 | Placental infarction |
| L267700 | Ragged placenta |
| L267800 | Placenta gritty |
| L267900 | Morbidly adherent placenta |
| L267A00 | Placenta accreta |
| L267z00 | Other placental conditions NOS |
| L268.00 | Other fetal problems |
| L268000 | Reduced fetal movements |
| L26y.00 | Other feto-placental problems |
| L26y.11 | Lithopaedian |
| L26y000 | Other feto-placental problems unspecified |
| L26y100 | Other feto-placental problems - delivered |
| L26y200 | Other feto-placental problems with antenatal problem |
| L26yz00 | Other feto-placental problems NOS |
| L26z.00 | Feto-placental problems NOS |
| L26z000 | Feto-placental problems NOS, unspecified |
| L26z100 | Feto-placental problems NOS - delivered |
| L26z200 | Feto-placental problems NOS with antenatal problem |
| L26zz00 | Feto-placental problems NOS |
| L27..00 | Polyhydramnios and hydramnios |
| L27..11 | Hydramnios |
| L270.00 | Polyhydramnios |
| L270000 | Polyhydramnios unspecified |
| L270100 | Polyhydramnios - delivered |
| L270200 | Polyhydramnios with antenatal problem |
| L270z00 | Polyhydramnios NOS |
| L27z.00 | Polyhydramnios NOS |
| L28..00 | Other problems of amniotic cavity and membranes |
| L280.00 | Oligohydramnios |
| L280000 | Oligohydramnios unspecified |
| L280100 | Oligohydramnios - delivered |
| L280200 | Oligohydramnios with antenatal problem |
| L280300 | Anhydramnios |
| L280z00 | Oligohydramnios NOS |
| L281.00 | Premature rupture of membranes |
| L281000 | Premature rupture of membranes unspecified |
| L281100 | Premature rupture of membranes - delivered |
| L281200 | Premature rupture of membranes with antenatal problem |
| L281300 | Prem rupture of membranes onset of labour within 24 hours |
| L281400 | Premature rupture of membranes, labour delayed by therapy |
| L281500 | Prem rupture of membranes onset of labour after 24 hours |
| L281z00 | Premature rupture of membranes NOS |
| L282.00 | Prolonged spontaneous or unspecified rupture of membranes |
| L282000 | Prolonged spont/unspec rupture of membranes unspecified |
| L282100 | Prolonged spont/unspec rupture of membranes - delivered |
| L282200 | Prolonged spont/unspec rupture of membranes with a/n problem |
| L282300 | Delay deliv after spontaneous or unsp rupture of membranes |
| L282z00 | Prolonged spontaneous/unspecified rupture of membranes NOS |
| L283.00 | Prolonged artificial rupture of membranes |
| L283000 | Prolonged artificial rupture of membranes unspecified |
| L283100 | Prolonged artificial rupture of membranes - delivered |
| L283200 | Prolonged artificial rupture of membranes with a/n problem |
| L283z00 | Prolonged artificial rupture of membranes NOS |
| L284.00 | Amniotic cavity infection |
| L284.11 | Amnionitis |
| L284.12 | Chorioamnionitis |
| L284.13 | Membranitis |
| L284.14 | Placentitis |
| L284000 | Amniotic cavity infection unspecified |
| L284100 | Amniotic cavity infection - delivered |
| L284200 | Amniotic cavity infection with antenatal problem |
| L284z00 | Amniotic cavity infection NOS |
| L28y.00 | Other problems of amniotic cavity and membranes |
| L28y.11 | Amnion nodosum |
| L28y.12 | Amniotic cyst |
| L28y.13 | Amniotic fluid leaking |
| L28y000 | Other problem of amniotic cavity and membranes unspecified |
| L28y100 | Other problem of amniotic cavity and membranes - delivered |
| L28y200 | Other amniotic/membrane problem with antenatal problem |
| L28y300 | Ragged membranes |
| L28yz00 | Other problem of amniotic cavity and membranes NOS |
| L28z.00 | Amniotic cavity and membrane problems NOS |
| L28z000 | Amniotic cavity and membrane problem NOS, unspecified |
| L28z100 | Amniotic cavity and membrane problem NOS - delivered |
| L28z200 | Amniotic cavity and membrane problem NOS with a/n problem |
| L28zz00 | Amniotic cavity and membrane problem NOS |
| L29..00 | Other problems affecting labour |
| L290.00 | Failed mechanical induction |
| L290.11 | Failed mechanical induction of labour |
| L290000 | Failed mechanical induction unspecified |
| L290100 | Failed mechanical induction - delivered |
| L290200 | Failed mechanical induction with antenatal problem |
| L290z00 | Failed mechanical induction NOS |
| L291.00 | Failed medical or unspecified induction |
| L291.11 | Failed medical induction of labour |
| L291000 | Failed medical or unspecified induction unspecified |
| L291100 | Failed medical or unspecified induction - delivered |
| L291200 | Failed medical or unspecified induction with a/n problem |
| L291z00 | Failed medical or unspecified induction NOS |
| L292.00 | Maternal pyrexia during labour, unspecified |
| L292000 | Unspecified maternal pyrexia during labour, unspecified |
| L292100 | Unspecified maternal pyrexia during labour - delivered |
| L292200 | Unspecified maternal pyrexia during labour with a/n problem |
| L292z00 | Unspecified maternal pyrexia during labour NOS |
| L293.00 | Septicaemia during labour |
| L293000 | Septicaemia during labour unspecified |
| L293100 | Septicaemia during labour - delivered |
| L293200 | Septicaemia during labour with antenatal problem |
| L293z00 | Septicaemia during labour NOS |
| L294.00 | Grand multiparity |
| L294000 | Grand multiparity unspecified |
| L294100 | Grand multiparity - delivered |
| L294200 | Grand multiparity with antenatal problem |
| L294z00 | Grand multiparity NOS |
| L295.00 | Elderly primigravida |
| L295000 | Elderly primigravida unspecified |
| L295100 | Elderly primigravida - delivered |
| L295200 | Elderly primigravida with antenatal problem |
| L295z00 | Elderly primigravida NOS |
| L296.00 | Vaginal delivery following previous caesarean section |
| L29y.00 | Other problems affecting labour |
| L29y000 | Other problems affecting labour unspecified |
| L29y100 | Other problems affecting labour - delivered |
| L29y200 | Other problems affecting labour with antenatal problem |
| L29yz00 | Other problems affecting labour NOS |
| L29z.00 | Problems affecting labour NOS |
| L29z000 | Problems affecting labour NOS unspecified |
| L29z100 | Problems affecting labour NOS - delivered |
| L29z200 | Problems affecting labour NOS with antenatal problem |
| L29zz00 | Problems affecting labour NOS |
| L2A..00 | Abnormal findings on antenatal screening of mother |
| L2A0.00 | Abnormal haematologic find on antenatal screening of mother |
| L2A1.00 | Abnormal biochemical finding on antenatal screen of mother |
| L2A2.00 | Abnormal cytological finding on antenatal screen of mother |
| L2A3.00 | Abnormal ultrasonic finding on antenatal screening of mother |
| L2A4.00 | Abnormal radiological finding on antenatal screen of mother |
| L2A5.00 | Abnormal chromosomal and genet find/antenat screen of mother |
| L2AX.00 | Abnormal finding on antenatal screening of mother |
| L2B..00 | Low weight gain in pregnancy |
| L2C..00 | Malnutrition in pregnancy |
| L2D..00 | Retained intrauterine contraceptive device in pregnancy |
| L2y..00 | Other specified risk factors in pregnancy |
| L2z..00 | Risk factors in pregnancy NOS |
| L3...00 | Complications occurring during labour and delivery |
| L30..00 | Obstructed labour |
| L300.00 | Obstructed labour due to fetal malposition |
| L300000 | Obstructed labour due to fetal malposition unspecified |
| L300100 | Obstructed labour due to fetal malposition - delivered |
| L300200 | Obstructed labour due to fetal malposition with a/n problem |
| L300300 | Obstructed labour due to breech presentation |
| L300400 | Obstructed labour due to face presentation |
| L300500 | Obstructed labour due to brow presentation |
| L300600 | Obstructed labour due to shoulder presentation |
| L300700 | Obstructed labour due to compound presentation |
| L300z00 | Obstructed labour due to fetal malposition NOS |
| L301.00 | Obstructed labour caused by bony pelvis |
| L301000 | Obstructed labour caused by bony pelvis unspecified |
| L301100 | Obstructed labour caused by bony pelvis - delivered |
| L301200 | Obstructed labour caused by bony pelvis with a/n problem |
| L301300 | Obstructed labour due to deformed pelvis |
| L301400 | Obstructed labour due to generally contracted pelvis |
| L301500 | Obstructed labour due to pelvic inlet contraction |
| L301600 | Obstruct labour due pelvic outlet and mid-cavity contract |
| L301700 | Obstructed labour due abnormality of maternal pelv organs |
| L301z00 | Obstructed labour caused by bony pelvis NOS |
| L302.00 | Obstructed labour caused by pelvic soft tissues |
| L302000 | Obstructed labour caused by pelvic soft tissues unspecified |
| L302100 | Obstructed labour caused by pelvic soft tissues - delivered |
| L302200 | Obstructed labour caused by pelvic soft tissues + a/n prob |
| L302z00 | Obstructed labour caused by pelvic soft tissues NOS |
| L303.00 | Deep transverse arrest (DTA) |
| L303000 | Deep transverse arrest unspecified |
| L303100 | Deep transverse arrest - delivered |
| L303200 | Deep transverse arrest with antenatal problem |
| L303z00 | Deep transverse arrest NOS |
| L304.00 | Persistent occipitoposterior or occipitoanterior position |
| L304000 | Persistent occipitopost/occipitoant position, unspecified |
| L304100 | Persistent occipitopost/occipitoant position - delivered |
| L304200 | Persistent occipitopost/occipitoant position + a/n problem |
| L304z00 | Persistent occipitoposterior/occipitoanterior position NOS |
| L305.00 | Shoulder dystocia |
| L305.11 | Impacted shoulders |
| L305000 | Shoulder dystocia unspecified |
| L305100 | Shoulder dystocia - delivered |
| L305200 | Shoulder dystocia with antenatal problem |
| L305z00 | Shoulder dystocia NOS |
| L306.00 | Locked twins |
| L306000 | Locked twins unspecified |
| L306100 | Locked twins - delivered |
| L306200 | Locked twins with antenatal problem |
| L306z00 | Locked twins NOS |
| L307.00 | Failed trial of labour unspecified |
| L307000 | Other failed trial of labour unspecified |
| L307100 | Other failed trial of labour - delivered |
| L307200 | Other failed trial of labour with antenatal problem |
| L307z00 | Failed trial of labour NOS |
| L308.00 | Failed forceps unspecified |
| L308000 | Other failed forceps, unspecified |
| L308100 | Other failed forceps - delivered |
| L308200 | Other failed forceps with antenatal problem |
| L308z00 | Failed forceps NOS |
| L309.00 | Failed ventouse extraction unspecified |
| L309000 | Other failed ventouse extraction, unspecified |
| L309100 | Other failed ventouse extraction - delivered |
| L309200 | Other failed ventouse extraction with antenatal problem |
| L309z00 | Failed ventouse extraction NOS |
| L30A.00 | Obstructed labour due to unusually large fetus |
| L30y.00 | Other causes of obstructed labour |
| L30y000 | Other causes of obstructed labour unspecified |
| L30y100 | Other causes of obstructed labour - delivered |
| L30y200 | Other causes of obstructed labour with antenatal problem |
| L30yz00 | Other causes of obstructed labour NOS |
| L30z.00 | Obstructed labour NOS |
| L30z000 | Obstructed labour NOS, unspecified |
| L30z100 | Obstructed labour NOS - delivered |
| L30z200 | Obstructed labour NOS with antenatal problem |
| L30zz00 | Obstructed labour NOS |
| L30zz11 | Dystocia NOS |
| L31..00 | Abnormal forces of labour |
| L310.00 | Primary uterine inertia |
| L310000 | Primary uterine inertia unspecified |
| L310100 | Primary uterine inertia - delivered |
| L310200 | Primary uterine inertia with antenatal problem |
| L310z00 | Primary uterine inertia NOS |
| L311.00 | Secondary uterine inertia |
| L311000 | Secondary uterine inertia unspecified |
| L311100 | Secondary uterine inertia - delivered |
| L311200 | Secondary uterine inertia with antenatal problem |
| L311z00 | Secondary uterine inertia NOS |
| L312.00 | Other uterine inertia |
| L312.11 | Atony of uterus |
| L312.12 | Poor contractions |
| L312000 | Other uterine inertia unspecified |
| L312100 | Other uterine inertia - delivered |
| L312200 | Other uterine inertia with antenatal problem |
| L312z00 | Other uterine inertia NOS |
| L313.00 | Precipitate labour |
| L313000 | Precipitate labour unspecified |
| L313100 | Precipitate labour - delivered |
| L313200 | Precipitate labour with antenatal problem |
| L313z00 | Precipitate labour NOS |
| L314.00 | Hypertonic uterine inertia |
| L314.11 | Bandl's retraction ring |
| L314.12 | Contraction ring (dystocia) |
| L314.13 | Hourglass uterine contraction |
| L314.14 | Incoordinate uterine action |
| L314.15 | Uterine dystocia NOS |
| L314.16 | Uterine or cervical spasm |
| L314000 | Hypertonic uterine inertia unspecified |
| L314100 | Hypertonic uterine inertia - delivered |
| L314200 | Hypertonic uterine inertia with antenatal problem |
| L314z00 | Hypertonic uterine inertia NOS |
| L31z.00 | Abnormality of forces of labour NOS |
| L31z000 | Abnormality of forces of labour NOS unspecified |
| L31z100 | Abnormality of forces of labour NOS - delivered |
| L31z200 | Abnormality of forces of labour NOS with antenatal problem |
| L31zz00 | Abnormality of forces of labour NOS |
| L32..00 | Long labour |
| L320.00 | Prolonged first stage |
| L320000 | Prolonged first stage unspecified |
| L320100 | Prolonged first stage - delivered |
| L320200 | Prolonged first stage with antenatal problem |
| L320z00 | Prolonged first stage NOS |
| L321.00 | Prolonged labour unspecified |
| L321000 | Unspecified prolonged labour, unspecified |
| L321100 | Unspecified prolonged labour - delivered |
| L321200 | Unspecified prolonged labour with antenatal problem |
| L321z00 | Prolonged labour NOS |
| L322.00 | Prolonged second stage |
| L322000 | Prolonged second stage unspecified |
| L322100 | Prolonged second stage - delivered |
| L322200 | Prolonged second stage with antenatal problem |
| L322z00 | Prolonged second stage NOS |
| L323.00 | Delayed delivery of second twin, triplet etc |
| L323000 | Delayed delivery second twin unspecified |
| L323100 | Delayed delivery second twin - delivered |
| L323200 | Delayed delivery second twin with antenatal problem |
| L323z00 | Delayed delivery second twin etc NOS |
| L32z.00 | Prolonged labour NOS |
| L33..00 | Umbilical cord complications |
| L330.00 | Prolapse of cord |
| L330.11 | Presentation of cord |
| L330000 | Prolapse of cord unspecified |
| L330100 | Prolapse of cord - delivered |
| L330200 | Prolapse of cord with antenatal problem |
| L330z00 | Prolapse of cord NOS |
| L331.00 | Cord tight round neck |
| L331000 | Cord tight round neck unspecified |
| L331100 | Cord tight round neck - delivered |
| L331200 | Cord tight round neck with antenatal problem |
| L331z00 | Cord tight round neck NOS |
| L332.00 | Cord tangled or knotted with compression |
| L332.11 | Knot in cord |
| L332000 | Cord tangled with compression unspecified |
| L332100 | Cord tangled with compression - delivered |
| L332200 | Cord tangled with compression with antenatal problem |
| L332z00 | Cord tangled or knotted with compression NOS |
| L333.00 | Other cord entanglement |
| L333000 | Other cord entanglement unspecified |
| L333100 | Other cord entanglement - delivered |
| L333200 | Other cord entanglement with antenatal problem |
| L333z00 | Other cord entanglement NOS |
| L334.00 | Short cord |
| L334000 | Short cord unspecified |
| L334100 | Short cord - delivered |
| L334200 | Short cord with antenatal problem |
| L334z00 | Short cord NOS |
| L335.00 | Vasa praevia |
| L335.11 | Velamentous insertion of cord |
| L335000 | Vasa praevia unspecified |
| L335100 | Vasa praevia - delivered |
| L335200 | Vasa praevia with antenatal problem |
| L335z00 | Vasa praevia NOS |
| L336.00 | Vascular lesions of cord |
| L336.11 | Bruising of cord |
| L336000 | Vascular lesions of cord unspecified |
| L336100 | Vascular lesions of cord - delivered |
| L336200 | Vascular lesions of cord with antenatal problem |
| L336z00 | Vascular lesions of cord NOS |
| L33y.00 | Other umbilical cord complications |
| L33y000 | Other umbilical cord complications unspecified |
| L33y100 | Other umbilical cord complications - delivered |
| L33y200 | Other umbilical cord complications with antenatal problem |
| L33yz00 | Other umbilical cord complications NOS |
| L33z.00 | Umbilical cord complications NOS |
| L33z000 | Umbilical cord complications NOS, unspecified |
| L33z100 | Umbilical cord complications NOS - delivered |
| L33z200 | Umbilical cord complications NOS with antenatal problem |
| L33zz00 | Umbilical cord complications NOS |
| L34..00 | Trauma to perineum and vulva during delivery |
| L34..11 | Perineal tear |
| L34..12 | Vulval delivery trauma |
| L340.00 | First degree perineal tear during delivery |
| L340.11 | Fourchette tear |
| L340.12 | Hymen tear |
| L340.13 | Labial tear |
| L340.14 | Vaginal tear |
| L340.15 | Vulval tear |
| L340000 | First degree perineal tear during delivery, unspecified |
| L340100 | First degree perineal tear during delivery - delivered |
| L340200 | First degree perineal tear during delivery with p/n problem |
| L340300 | Labial tear during delivery |
| L340400 | Fourchette tear during delivery |
| L340500 | Vulval tear during delivery |
| L340600 | Vaginal tear during delivery |
| L340z00 | First degree perineal tear during delivery NOS |
| L341.00 | Second degree perineal tear during delivery |
| L341.11 | Pelvic floor tear |
| L341.12 | Perineal muscle tear |
| L341.13 | Vaginal muscle tear |
| L341000 | Second degree perineal tear during delivery, unspecified |
| L341100 | Second degree perineal tear during delivery - delivered |
| L341200 | Second degree perineal tear during delivery with p/n prob |
| L341z00 | Second degree perineal tear during delivery NOS |
| L342.00 | Third degree perineal tear during delivery |
| L342.11 | Anal sphincter tear |
| L342000 | Third degree perineal tear during delivery, unspecified |
| L342100 | Third degree perineal tear during delivery - delivered |
| L342200 | Third degree perineal tear during delivery with p/n problem |
| L342z00 | Third degree perineal tear during delivery NOS |
| L343.00 | Fourth degree perineal tear during delivery |
| L343.11 | Mucosal tear of anus or rectum |
| L343000 | Fourth degree perineal tear during delivery, unspecified |
| L343100 | Fourth degree perineal tear during delivery - delivered |
| L343200 | Fourth degree perineal tear during delivery with p/n problem |
| L343z00 | Fourth degree perineal tear during delivery NOS |
| L344.00 | Unspecified perineal laceration during delivery |
| L344000 | Unspecified perineal laceration during delivery, unspecified |
| L344100 | Unspecified perineal laceration during delivery - delivered |
| L344200 | Unspecified perineal laceration during delivery + p/n prob |
| L344z00 | Unspecified perineal laceration during delivery NOS |
| L345.00 | Vulval and perineal haematoma during delivery |
| L345.11 | Perineal haematoma |
| L345.12 | Vulval and perineal haematoma during delivery |
| L345000 | Vulval and perineal haematoma during delivery, unspecified |
| L345100 | Vulval and perineal haematoma during delivery - delivered |
| L345200 | Vulval and perineal haematoma during delivery + p/n problem |
| L345z00 | Vulval and perineal haematoma during delivery NOS |
| L34y.00 | Other vulval and perineal trauma during delivery |
| L34y000 | Other vulval/perineal trauma during delivery, unspecified |
| L34y100 | Other vulval/perineal trauma during delivery- delivered |
| L34y200 | Other vulval/perineal trauma during delivery + p/n problem |
| L34yz00 | Other vulval/perineal trauma during delivery NOS |
| L34z.00 | Vulval/perineal trauma during delivery NOS |
| L34z000 | Vulval/perineal trauma during delivery NOS unspec |
| L34z100 | Vulval/perineal trauma during delivery NOS - delivered |
| L34z200 | Vulval/perineal trauma during delivery NOS with p/n problem |
| L34zz00 | Vulval/perineal trauma during delivery NOS |
| L35..00 | Other obstetric trauma |
| L350.00 | Ruptured uterus before labour |
| L350000 | Rupture of uterus before labour unspecified |
| L350100 | Rupture of uterus before labour - delivered |
| L350200 | Rupture of uterus before labour with antenatal problem |
| L350z00 | Rupture of uterus before labour NOS |
| L351.00 | Rupture of uterus during and after labour |
| L351000 | Rupture of uterus during and after labour unspecified |
| L351100 | Rupture of uterus during and after labour - delivered |
| L351200 | Rupture of uterus during/after labour - deliv with p/n prob |
| L351300 | Rupture of uterus during/after labour with postnatal problem |
| L351z00 | Rupture of uterus during and after labour NOS |
| L352.00 | Obstetric inversion of uterus |
| L352.11 | Inversion of uterus - obstetric |
| L352000 | Obstetric inversion of uterus unspecified |
| L352100 | Obstetric inversion of uterus - delivered with p/n problem |
| L352200 | Obstetric inversion of uterus with postnatal problem |
| L352z00 | Obstetric inversion of uterus NOS |
| L353.00 | Obstetric laceration of cervix |
| L353.11 | Laceration of cervix - obstetric |
| L353.12 | Tear of cervix - obstetric |
| L353000 | Obstetric laceration of cervix unspecified |
| L353100 | Obstetric laceration of cervix - delivered |
| L353200 | Obstetric laceration of cervix with postnatal problem |
| L353z00 | Obstetric laceration of cervix NOS |
| L354.00 | Obstetric high vaginal laceration |
| L354.11 | High vaginal laceration - obstetric |
| L354.12 | High vaginal tear - obstetric |
| L354000 | Obstetric high vaginal laceration unspecified |
| L354100 | Obstetric high vaginal laceration - delivered |
| L354200 | Obstetric high vaginal laceration with postnatal problem |
| L354z00 | Obstetric high vaginal laceration NOS |
| L355.00 | Other obstetric pelvic organ damage |
| L355.11 | Bladder injury - obstetric |
| L355.12 | Urethra injury - obstetric |
| L355000 | Other obstetric pelvic organ damage unspecified |
| L355100 | Other obstetric pelvic organ damage - delivered |
| L355200 | Other obstetric pelvic organ damage with postnatal problem |
| L355z00 | Other obstetric pelvic organ damage NOS |
| L356.00 | Obstetric trauma damaging pelvic joints and ligaments |
| L356.11 | Obstetric pelvic joint damage |
| L356.12 | Obstetric pelvic ligament damage |
| L356.13 | Pubic symphysis separation |
| L356.14 | Symphysis pubis separation |
| L356000 | Obstetric damage to pelvic joints and ligaments unspecified |
| L356100 | Obstetric damage to pelvic joints and ligaments - delivered |
| L356200 | Obstetric damage to pelvic joints and ligaments + p/n prob |
| L356z00 | Obstetric damage to pelvic joints and ligaments NOS |
| L357.00 | Obstetric trauma causing pelvic haematoma |
| L357000 | Obstetric pelvic haematoma unspecified |
| L357100 | Obstetric pelvic haematoma - delivered |
| L357200 | Obstetric pelvic haematoma - delivered with p/n problem |
| L357300 | Obstetric pelvic haematoma with postnatal problem |
| L357z00 | Obstetric trauma causing pelvic haematoma NOS |
| L35y.00 | Other obstetric trauma OS |
| L35y000 | Other obstetric trauma unspecified |
| L35y100 | Other obstetric trauma - delivered |
| L35y200 | Other obstetric trauma - delivered with postnatal problem |
| L35y300 | Other obstetric trauma with antenatal problem |
| L35y400 | Other obstetric trauma with postnatal problem |
| L35yz00 | Other obstetric trauma NOS |
| L35z.00 | Obstetric trauma NOS |
| L35z000 | Obstetric trauma NOS, unspecified |
| L35z100 | Obstetric trauma NOS - delivered |
| L35z200 | Obstetric trauma NOS - delivered with postnatal problem |
| L35z300 | Obstetric trauma NOS with antenatal problem |
| L35z400 | Obstetric trauma NOS with postnatal problem |
| L35zz00 | Obstetric trauma NOS |
| L36..00 | Postpartum haemorrhage (PPH) |
| L36..11 | Bleeding postpartum |
| L360.00 | Third-stage postpartum haemorrhage |
| L360.11 | Retained placenta NOS |
| L360000 | Third-stage postpartum haemorrhage unspecified |
| L360100 | Third-stage postpartum haemorrhage - deliv with p/n problem |
| L360200 | Third-stage postpartum haemorrhage with postnatal problem |
| L360z00 | Third-stage postpartum haemorrhage NOS |
| L361.00 | Other immediate postpartum haemorrhage |
| L361000 | Other immediate postpartum haemorrhage unspecified |
| L361100 | Other immediate postpartum haemorrhage - deliv with p/n prob |
| L361200 | Other immediate postpartum haemorrhage with postnatal prob |
| L361z00 | Other immediate postpartum haemorrhage NOS |
| L362.00 | Secondary and delayed postpartum haemorrhage |
| L362000 | Secondary postpartum haemorrhage unspecified |
| L362100 | Secondary postpartum haemorrhage - deliv with postnatal prob |
| L362200 | Secondary postpartum haemorrhage with postnatal problem |
| L362z00 | Secondary and delayed postpartum haemorrhage NOS |
| L363.00 | Postpartum coagulation defects |
| L363.11 | Afibrinogenaemia - postpartum |
| L363.12 | Fibrinolysis - postpartum |
| L363000 | Postpartum coagulation defects unspecified |
| L363100 | Postpartum coagulation defects - delivered with p/n problem |
| L363200 | Postpartum coagulation defects with postnatal problem |
| L363z00 | Postpartum coagulation defects NOS |
| L364.00 | Minor postpartum haemorrhage |
| L365.00 | Major postpartum haemorrhage |
| L366.00 | Quant of postpartum blood loss |
| L36z.00 | Postpartum haemorrhage NOS |
| L37..00 | Retained placenta or membranes with no haemorrhage |
| L37..11 | Retained membrane without haemorrhage |
| L37..12 | Retained placenta without haemorrhage |
| L370.00 | Retained placenta with no haemorrhage |
| L370.11 | Placenta accreta without haemorrhage |
| L370000 | Retained placenta with no haemorrhage unspecified |
| L370100 | Retained placenta with no haemorrhage - deliv with p/n prob |
| L370200 | Retained placenta with no haemorrhage with postnatal problem |
| L370z00 | Retained placenta with no haemorrhage NOS |
| L370z11 | Retained placenta without haemorrhage |
| L371.00 | Retained portion of placenta or membranes - no haemorrhage |
| L371000 | Retained products with no haemorrhage unspecified |
| L371100 | Retained products with no haemorrhage - deliv with p/n prob |
| L371200 | Retained products with no haemorrhage with postnatal problem |
| L371z00 | Retained products with no haemorrhage NOS |
| L37z.00 | Retained placenta or membranes with no haemorrhage NOS |
| L38..00 | Complications of anaesthesia during labour and delivery |
| L380.00 | Obstetric anaesthesia with pulmonary complications |
| L380.11 | Mendelson's syndrome |
| L380000 | Obstetric anaesthesia with pulmonary complications unsp |
| L380100 | Obstetric anaesthesia with pulmonary complications - deliv |
| L380200 | Obstetric anaesthesia with pulmonary comp - deliv + p/n prob |
| L380300 | Obstetric anaesthesia with pulmonary comp with a/n problem |
| L380400 | Obstetric anaesthesia with pulmonary comp with p/n problem |
| L380z00 | Obstetric anaesthesia with pulmonary complications NOS |
| L381.00 | Obstetric anaesthesia with cardiac complications |
| L381000 | Obstetric anaesthesia with cardiac complications unspecified |
| L381100 | Obstetric anaesthesia with cardiac complications - delivered |
| L381200 | Obstetric anaesthesia with cardiac comp - deliv + p/n prob |
| L381300 | Obstetric anaesthesia with cardiac comp with antenatal prob |
| L381400 | Obstetric anaesthesia with cardiac comp with postnatal prob |
| L381z00 | Obstetric anaesthesia with cardiac complications NOS |
| L382.00 | Obstetric anaesthesia with CNS complications |
| L382000 | Obstetric anaesthesia with CNS complications unspecified |
| L382100 | Obstetric anaesthesia with CNS complications - delivered |
| L382200 | Obstetric anaesthesia with CNS comp - deliv with p/n problem |
| L382300 | Obstetric anaesthesia with CNS comp with antenatal problem |
| L382400 | Obstetric anaesthesia with CNS comp with postnatal problem |
| L382z00 | Obstetric anaesthesia with CNS complication NOS |
| L383.00 | Obstetric toxic reaction to local anaesthesia |
| L383000 | Toxic reaction to local anaesthesia during pregnancy |
| L383100 | Toxic reaction to local anaesthesia during the puerperium |
| L384.00 | Obstetric spinal and epidural anaesthesia-induced headache |
| L384000 | Spinal+epidural anaesthesia-inducd headache during pregnancy |
| L384100 | Spinal/epidural anaesth-induced headache during puerp |
| L385.00 | Failed or difficult intubation during pregnancy |
| L386.00 | Toxic reaction to local anaesthesia during labour and deliv |
| L387.00 | Spinal/epidural anesth-induced headache dur labour/delivery |
| L388.00 | Cardiac comps of anaesthesia during labour and delivery |
| L389.00 | CNS comps of anaesthesia during labour and delivery |
| L38A.00 | Failed or difficult intubation during labour and delivery |
| L38B.00 | Failed or difficult intubation during the puerperium |
| L38X.00 | Complication of anaesthesia during labour and deliv unsp |
| L38y.00 | Other complications of obstetric anaesthesia |
| L38y000 | Other complications of obstetric anaesthesia unspecified |
| L38y100 | Other complications of obstetric anaesthesia - delivered |
| L38y200 | Other complications of obstetric anaesthesia -del + p/n prob |
| L38y300 | Other complications of obstetric anaesthesia + a/n problem |
| L38y400 | Other complications of obstetric anaesthesia + p/n problem |
| L38yz00 | Other complications of obstetric anaesthesia NOS |
| L38z.00 | Obstetric anaesthetic complications NOS |
| L38z000 | Obstetric anaesthetic complications NOS, unspecified |
| L38z100 | Obstetric anaesthetic complications NOS - delivered |
| L38z200 | Obstetric anaesthetic complications NOS - deliv + p/n prob |
| L38z300 | Obstetric anaesthetic complications NOS with a/n problem |
| L38z400 | Obstetric anaesthetic complications NOS with p/n problem |
| L38zz00 | Obstetric anaesthetic complications NOS |
| L39..00 | Other complications of labour and delivery NEC |
| L390.00 | Maternal distress |
| L390000 | Maternal distress unspecified |
| L390100 | Maternal distress - delivered |
| L390200 | Maternal distress - delivered with postnatal problem |
| L390300 | Maternal distress with antenatal problem |
| L390400 | Maternal distress with postnatal problem |
| L390z00 | Maternal distress NOS |
| L391.00 | Obstetric shock |
| L391000 | Obstetric shock unspecified |
| L391100 | Obstetric shock - delivered |
| L391200 | Obstetric shock - delivered with postnatal problem |
| L391300 | Obstetric shock with antenatal problem |
| L391400 | Obstetric shock with postnatal problem |
| L391z00 | Obstetric shock NOS |
| L392.00 | Maternal hypotension syndrome |
| L392000 | Maternal hypotension syndrome unspecified |
| L392100 | Maternal hypotension syndrome - delivered |
| L392200 | Maternal hypotension syndrome - delivered with p/n problem |
| L392300 | Maternal hypotension syndrome with antenatal problem |
| L392400 | Maternal hypotension syndrome with postnatal problem |
| L392z00 | Maternal hypotension syndrome NOS |
| L393.00 | Acute renal failure following labour and delivery |
| L393000 | Post-delivery acute renal failure unspecified |
| L393100 | Post-delivery acute renal failure - delivered with p/n prob |
| L393200 | Post-delivery acute renal failure with postnatal problem |
| L393z00 | Post-delivery acute renal failure NOS |
| L394.00 | Other complications of obstetric procedures |
| L394000 | Other complications of obstetric procedures unspecified |
| L394100 | Other complications of obstetric procedures - delivered |
| L394200 | Other complications of obstetric procedures - del +p/n prob |
| L394300 | Other complications of obstetric procedures with p/n problem |
| L394500 | Infection of obstetric surgical wound |
| L394600 | Haematoma of obstetric wound |
| L394z00 | Other complications of obstetric procedures NOS |
| L395.00 | Forceps delivery |
| L395.11 | Keilland's forceps delivery |
| L395.12 | Neville - Barnes forceps delivery |
| L395.13 | Simpson's forceps delivery |
| L395000 | Forceps delivery unspecified |
| L395100 | Forceps delivery - delivered |
| L395200 | Low forceps delivery |
| L395300 | Mid-cavity forceps delivery |
| L395400 | Delivery by combination of forceps and vacuum extractor |
| L395500 | Mid-cavity forceps with rotation |
| L395z00 | Forceps delivery NOS |
| L396.00 | Vacuum extractor delivery |
| L396.11 | Ventouse delivery |
| L396000 | Vacuum extractor delivery unspecified |
| L396100 | Vacuum extractor delivery - delivered |
| L396z00 | Vacuum extractor delivery NOS |
| L397.00 | Breech extraction |
| L397000 | Breech extraction unspecified |
| L397100 | Breech extraction - delivered |
| L397z00 | Breech extraction NOS |
| L398.00 | Caesarean delivery |
| L398000 | Caesarean delivery unspecified |
| L398100 | Caesarean delivery - delivered |
| L398200 | Caesarean section - pregnancy at term |
| L398300 | Delivery by elective caesarean section |
| L398400 | Delivery by emergency caesarean section |
| L398500 | Delivery by caesarean hysterectomy |
| L398600 | Caesarean delivery following previous Caesarean delivery |
| L398z00 | Caesarean delivery NOS |
| L399.00 | Destructive operation for delivery |
| L39A.00 | Death obst cse occur more 42 day less than one yr aft deliv |
| L39A000 | Dth dir ob cs 42dy 1yr aft del |
| L39A100 | Dth ind ob cs 42dy 1yr aft del |
| L39B.00 | Death from sequelae of direct obstetric causes |
| L39X.00 | Obstetric death of unspecified cause |
| L39y.00 | Other complications of labour and delivery |
| L39y000 | Other complications of labour and delivery unspecified |
| L39y100 | Other complications of labour and delivery - delivered |
| L39y200 | Other complications of labour and delivery - deliv +p/n prob |
| L39y300 | Other complications of labour and delivery with a/n problem |
| L39y400 | Other complications of labour and delivery with p/n problem |
| L39y411 | Postnatal vaginal discomfort |
| L39y412 | Vaginal discomfort postnatal |
| L39y500 | Maternal exhaustion |
| L39yz00 | Other complications of labour and delivery NOS |
| L39z.00 | Complications of labour and delivery NOS |
| L39z000 | Complications of labour and delivery NOS, unspecified |
| L39z100 | Complications of labour and delivery NOS - delivered |
| L39z200 | Complications of labour and delivery NOS - del + p/n problem |
| L39z300 | Complications of labour and delivery NOS with antenatal prob |
| L39z400 | Complications of labour and delivery NOS with p/n problem |
| L39zz00 | Complications of labour and delivery NOS |
| L3A..00 | Intrapartum haemorrhage with coagulation defect |
| L3X..00 | Intrapartum haemorrhage, unspecified |
| L3y..00 | Other specified complications of labour or delivery |
| L3z..00 | Complications of labour and delivery NOS |
| L4...00 | Complications of the puerperium |
| L40..00 | Major puerperal infection |
| L40..11 | Sepsis - puerperal |
| L400.00 | Puerperal endometritis |
| L400000 | Puerperal endometritis unspecified |
| L400100 | Puerperal endometritis - delivered with postnatal comp |
| L400200 | Puerperal endometritis with postnatal complication |
| L400z00 | Puerperal endometritis NOS |
| L401.00 | Puerperal salpingitis |
| L401000 | Puerperal salpingitis unspecified |
| L401100 | Puerperal salpingitis - delivered with postnatal comp |
| L401200 | Puerperal salpingitis with postnatal complication |
| L401z00 | Puerperal salpingitis NOS |
| L402.00 | Puerperal peritonitis |
| L402000 | Puerperal peritonitis unspecified |
| L402100 | Puerperal peritonitis - delivered with postnatal comp |
| L402200 | Puerperal peritonitis with postnatal complication |
| L402z00 | Puerperal peritonitis NOS |
| L403.00 | Puerperal septicaemia |
| L403000 | Puerperal septicaemia unspecified |
| L403100 | Puerperal septicaemia - delivered with postnatal comp |
| L403200 | Puerperal septicaemia with postnatal complication |
| L403z00 | Puerperal septicaemia NOS |
| L40z.00 | Major puerperal infection NOS |
| L40z000 | Major puerperal infection NOS, unspecified |
| L40z100 | Major puerperal infection NOS - delivered with p/n comp |
| L40z200 | Major puerperal infection NOS with postnatal complication |
| L40zz00 | Major puerperal infection NOS |
| L41..00 | Venous complications of pregnancy and the puerperium |
| L41..11 | Varicose veins - obstetric |
| L410.00 | Varicose veins of legs in pregnancy and the puerperium |
| L410000 | Varicose veins of legs in pregnancy/puerperium unspecified |
| L410100 | Varicose veins of legs in pregnancy/puerperium - delivered |
| L410200 | Varicose veins of legs in pregnancy/puerperium -del+p/n comp |
| L410300 | Varicose veins of legs in pregnancy/puerperium + a/n comp |
| L410400 | Varicose veins of legs in pregnancy/puerperium + p/n comp |
| L410500 | Varicose veins of legs in pregnancy |
| L410600 | Varicose veins of legs in the puerperium |
| L410z00 | Varicose veins of legs in pregnancy and puerperium NOS |
| L411.00 | VV's of perineum/vulva in pregnancy/puerperium |
| L411.11 | Perineal obstetric varicose veins |
| L411.12 | Vulval obstetric varicose veins |
| L411000 | VV's of perineum/vulva in pregnancy/puerperium unspecified |
| L411100 | VV's of perineum/vulva in pregnancy/puerperium - delivered |
| L411200 | VV's of perineum/vulva in pregnancy/puerperium -del+p/n comp |
| L411300 | VV's of perineum/vulva in pregnancy/puerperium + a/n comp |
| L411400 | VV's of perineum/vulva in pregnancy/puerperium + p/n comp |
| L411500 | Genital varices in pregnancy |
| L411511 | Perineal varices in pregnancy |
| L411512 | Vaginal varices in pregnancy |
| L411513 | Vulval varices in pregnancy |
| L411600 | Genital varices in the puerperium |
| L411611 | Perineal varices in the puerperium |
| L411612 | Vaginal varices in the puerperium |
| L411613 | Vulval varices in the puerperium |
| L411z00 | Varicose veins of perineum/vulva in pregnancy/puerperium NOS |
| L412.00 | Superficial thrombophlebitis in pregnancy and the puerperium |
| L412000 | Superficial thrombophlebitis in pregnancy/puerperium unsp |
| L412100 | Superficial thrombophlebitis in pregnancy/puerperium -deliv |
| L412200 | Superficial thrombophleb in preg/puerperium - del + p/n comp |
| L412211 | Phlebitis - postpartum |
| L412212 | Puerperal phlebitis |
| L412300 | Superficial thrombophlebitis in preg/puerperium + a/n comp |
| L412400 | Superficial thrombophlebitis in preg/puerperium + p/n comp |
| L412500 | Superficial thrombophlebitis in pregnancy |
| L412511 | Thrombophlebitis of legs in pregnancy |
| L412600 | Superficial thrombophlebitis in the puerperium |
| L412611 | Thombophlebitis of legs in the puerperium |
| L412z00 | Superficial thrombophlebitis in pregnancy and puerperium NOS |
| L413.00 | Antenatal deep vein thrombosis |
| L413.11 | DVT - deep venous thrombosis, antenatal |
| L413000 | Antenatal deep vein thrombosis unspecified |
| L413100 | Antenatal deep vein thrombosis - delivered |
| L413200 | Antenatal deep vein thrombosis with antenatal complication |
| L413z00 | Antenatal deep vein thrombosis NOS |
| L414.00 | Postnatal deep vein thrombosis |
| L414.11 | DVT - deep venous thrombosis, postnatal |
| L414.12 | Phlegmasia alba dolens - obstetric |
| L414000 | Postnatal deep vein thrombosis unspecified |
| L414100 | Postnatal deep vein thrombosis - delivered with p/n comp |
| L414200 | Postnatal deep vein thrombosis with postnatal complication |
| L414z00 | Postnatal deep vein thrombosis NOS |
| L415.00 | Other phlebitis and thrombosis in pregnancy and puerperium |
| L415000 | Other phlebitis/thrombosis in pregnancy/puerperium unsp |
| L415100 | Other phlebitis/thrombosis in pregnancy/puerperium - deliv |
| L415200 | Other phlebitis/thrombosis in preg/puerperium -del +p/n comp |
| L415300 | Other phlebitis/thrombosis in preg/puerperium + a/n comp |
| L415400 | Other phlebitis/thrombosis in preg/puerperium + p/n comp |
| L415500 | Other phlebitis in pregnancy |
| L415600 | Other phlebitis in the puerperium |
| L415z00 | Other phlebitis/thrombosis in pregnancy and puerperium NOS |
| L416.00 | Haemorrhoids in pregnancy and the puerperium |
| L416.11 | Piles - obstetric |
| L416000 | Haemorrhoids in pregnancy and the puerperium unspecified |
| L416100 | Haemorrhoids in pregnancy and the puerperium - delivered |
| L416200 | Haemorrhoids in pregnancy and puerperium - deliv + p/n comp |
| L416300 | Haemorrhoids in pregnancy and puerperium with a/n comp |
| L416400 | Haemorrhoids in pregnancy and puerperium with p/n comp |
| L416500 | Haemorrhoids in the puerperium |
| L416600 | Haemorrhoids in pregnancy |
| L416z00 | Haemorrhoids in pregnancy and the puerperium NOS |
| L417.00 | Obstetric cerebral venous thrombosis |
| L417000 | Cerebral venous thrombosis in pregnancy |
| L417100 | Cerebral venous thrombosis in the puerperium |
| L41y.00 | Other venous complication of pregnancy and the puerperium |
| L41y000 | Other venous complication of pregnancy/puerperium unsp |
| L41y100 | Other venous complication of pregnancy/puerperium -delivered |
| L41y200 | Other venous comp of pregnancy/puerperium - deliv + p/n comp |
| L41y300 | Other venous comp of pregnancy/puerperium + a/n comp |
| L41y400 | Other venous comp of pregnancy/puerperium + p/n comp |
| L41yz00 | Other venous complication of pregnancy and puerperium NOS |
| L41z.00 | Venous complications of pregnancy and puerperium NOS |
| L41z000 | Venous complication pregnancy/puerperium NOS unspecified |
| L41z100 | Venous complication pregnancy and puerperium NOS - delivered |
| L41z200 | Venous complication pregnancy/puerperium NOS - del +p/n comp |
| L41z300 | Venous complication pregnancy/puerperium NOS + a/n comp |
| L41z400 | Venous complication pregnancy/puerperium NOS + p/n comp |
| L41z500 | Venous complication of pregnancy, unspecified |
| L41z511 | Gestational phlebitis NOS |
| L41z512 | Gestational phlebopathy NOS |
| L41z513 | Gestational thrombosis NOS |
| L41z600 | Venous complication in the puerperium, unspecified |
| L41z611 | Puerperal phlebitis NOS |
| L41z612 | Puerperal phlebopathy NOS |
| L41z613 | Puerperal thrombosis NOS |
| L41zz00 | Venous complication of pregnancy and puerperium NOS |
| L42..00 | Puerperal pyrexia of unknown origin |
| L420.00 | Puerperal pyrexia of unknown origin |
| L420000 | Puerperal pyrexia of unknown origin unspecified |
| L420100 | Puerperal pyrexia of unknown origin - delivered + p/n comp |
| L420200 | Puerperal pyrexia of unknown origin with p/n complication |
| L420z00 | Puerperal pyrexia NOS |
| L42z.00 | Puerperal pyrexia NOS |
| L43..00 | Obstetric pulmonary embolism |
| L43..11 | Obstetric pulmonary embolus |
| L430.00 | Obstetric air pulmonary embolism |
| L430000 | Obstetric air pulmonary embolism unspecified |
| L430100 | Obstetric air pulmonary embolism - delivered |
| L430200 | Obstetric air pulm embolism - delivered + p/n complication |
| L430300 | Obstetric air pulmonary embolism with a/n complication |
| L430400 | Obstetric air pulmonary embolism with p/n complication |
| L430z00 | Obstetric air pulmonary embolism NOS |
| L431.00 | Amniotic fluid pulmonary embolism |
| L431000 | Amniotic fluid pulmonary embolism unspecified |
| L431100 | Amniotic fluid pulmonary embolism - delivered |
| L431200 | Amniotic fluid pulm embolism - delivered + p/n complication |
| L431300 | Amniotic fluid pulmonary embolism with a/n complication |
| L431400 | Amniotic fluid pulmonary embolism with p/n complication |
| L431z00 | Amniotic fluid pulmonary embolism NOS |
| L432.00 | Obstetric blood-clot pulmonary embolism |
| L432000 | Obstetric blood-clot pulmonary embolism unspecified |
| L432100 | Obstetric blood-clot pulmonary embolism - delivered |
| L432200 | Obstetric blood-clot pulm embolism - delivered with p/n comp |
| L432300 | Obstetric blood-clot pulmonary embolism + a/n complication |
| L432400 | Obstetric blood-clot pulmonary embolism + p/n complication |
| L432z00 | Obstetric blood-clot pulmonary embolism NOS |
| L433.00 | Obstetric pyaemic and septic pulmonary embolism |
| L433.11 | Pyaemic obstetric embolism |
| L433.12 | Septic obstetric embolism |
| L433000 | Obstetric pyaemic and septic pulmonary embolism unspecified |
| L433100 | Obstetric pyaemic and septic pulmonary embolism - delivered |
| L433200 | Obstetric pyaemic and septic pulm embolism - deliv +p/n comp |
| L433300 | Obstetric pyaemic and septic pulm embolism + a/n comp |
| L433400 | Obstetric pyaemic and septic pulm embolism + p/n comp |
| L433z00 | Obstetric pyaemic and septic pulmonary embolism NOS |
| L43y.00 | Other obstetric pulmonary embolism |
| L43y.11 | Fat embolism - obstetric |
| L43y000 | Other obstetric pulmonary embolism unspecified |
| L43y100 | Other obstetric pulmonary embolism - delivered |
| L43y200 | Other obstetric pulmonary embolism - delivered + p/n comp |
| L43y300 | Other obstetric pulmonary embolism with antenatal comp |
| L43y400 | Other obstetric pulmonary embolism with postnatal comp |
| L43yz00 | Other obstetric pulmonary embolism NOS |
| L43z.00 | Obstetric pulmonary embolism NOS |
| L43z000 | Obstetric pulmonary embolism NOS, unspecified |
| L43z100 | Obstetric pulmonary embolism NOS - delivered |
| L43z200 | Obstetric pulmonary embolism NOS - delivered with p/n comp |
| L43z300 | Obstetric pulmonary embolism NOS with antenatal complication |
| L43z400 | Obstetric pulmonary embolism NOS with postnatal complication |
| L43zz00 | Obstetric pulmonary embolism NOS |
| L44..00 | Other complications of the puerperium NEC |
| L440.00 | Cerebrovascular disorders in the puerperium |
| L440.11 | CVA - cerebrovascular accident in the puerperium |
| L440.12 | Stroke in the puerperium |
| L440000 | Puerperal cerebrovascular disorder unspecified |
| L440100 | Puerperal cerebrovascular disorder - delivered |
| L440200 | Puerperal cerebrovascular disorder - delivered with p/n comp |
| L440300 | Puerperal cerebrovascular disorder with antenatal comp |
| L440400 | Puerperal cerebrovascular disorder with postnatal comp |
| L440z00 | Puerperal cerebrovascular disorder NOS |
| L441.00 | Caesarean wound disruption |
| L441000 | Caesarean wound disruption unspecified |
| L441100 | Caesarean wound disruption - delivered with p/n complication |
| L441200 | Caesarean wound disruption with postnatal complication |
| L441z00 | Caesarean wound disruption NOS |
| L442.00 | Obstetric perineal wound disruption |
| L442.11 | Breakdown of perineum |
| L442.12 | Episiotomy breakdown |
| L442000 | Obstetric perineal wound disruption unspecified |
| L442100 | Obstetric perineal wound disruption - deliv + p/n comp |
| L442200 | Obstetric perineal wound disruption with p/n complication |
| L442z00 | Obstetric perineal wound disruption NOS |
| L443.00 | Other complication of obstetric surgical wound |
| L443.11 | Haematoma - perineal wound |
| L443.12 | Infection - perineal wound |
| L443000 | Other complication of obstetric surgical wound unspecified |
| L443100 | Other complication obstetric surg wound -delivered +p/n comp |
| L443200 | Other complication obstetric surgical wound with p/n comp |
| L443z00 | Other complication of obstetric surgical wound NOS |
| L444.00 | Placental polyp |
| L444000 | Placental polyp unspecified |
| L444100 | Placental polyp - delivered with postnatal complication |
| L444200 | Placental polyp with postnatal complication |
| L444z00 | Placental polyp NOS |
| L44y.00 | Other complications of the puerperium |
| L44y.11 | Subinvolution of uterus in the puerperium |
| L44y000 | Other complications of the puerperium unspecified |
| L44y100 | Other complications of the puerperium - delivered + p/n comp |
| L44y200 | Other complications of the puerperium with p/n complication |
| L44yz00 | Other complications of the puerperium NOS |
| L44yz11 | Blood dyscrasia puerperal |
| L44z.00 | Complications of the puerperium NOS |
| L44z000 | Complications of the puerperium NOS, unspecified |
| L44z100 | Complications of the puerperium NOS - delivered + p/n comp |
| L44z200 | Complications of the puerperium NOS with postnatal comp |
| L44zz00 | Complications of the puerperium NOS |
| L45..00 | Obstetric breast infections |
| L450.00 | Obstetric nipple infection |
| L450.11 | Abscess of nipple - obstetric |
| L450.12 | Nipple infection - obstetric |
| L450000 | Obstetric nipple infection unspecified |
| L450100 | Obstetric nipple infection - delivered |
| L450200 | Obstetric nipple infection - delivered with p/n complication |
| L450300 | Obstetric nipple infection with antenatal complication |
| L450400 | Obstetric nipple infection with postnatal complication |
| L450z00 | Obstetric nipple infection NOS |
| L451.00 | Obstetric breast abscess |
| L451.11 | Purulent mastitis - obstetric |
| L451000 | Obstetric breast abscess unspecified |
| L451100 | Obstetric breast abscess - delivered |
| L451200 | Obstetric breast abscess - deliv with postnatal complication |
| L451300 | Obstetric breast abscess with antenatal complication |
| L451400 | Obstetric breast abscess with postnatal complication |
| L451z00 | Obstetric breast abscess NOS |
| L452.00 | Obstetric nonpurulent mastitis |
| L452.11 | Lymphangitis of breast - obstetric |
| L452000 | Obstetric nonpurulent mastitis unspecified |
| L452100 | Obstetric nonpurulent mastitis - delivered |
| L452200 | Obstetric nonpurulent mastitis - deliv with p/n complication |
| L452300 | Obstetric nonpurulent mastitis with antenatal complication |
| L452400 | Obstetric nonpurulent mastitis with postnatal complication |
| L452z00 | Obstetric nonpurulent mastitis NOS |
| L45y.00 | Other obstetric breast infections |
| L45y000 | Other obstetric breast infection unspecified |
| L45y100 | Other obstetric breast infection - delivered |
| L45y200 | Other obstetric breast infection - deliv with p/n comp |
| L45y300 | Other obstetric breast infection with antenatal complication |
| L45y400 | Other obstetric breast infection with postnatal complication |
| L45yz00 | Other obstetric breast infection NOS |
| L45z.00 | Obstetric breast infection NOS |
| L45z000 | Obstetric breast infection NOS, unspecified |
| L45z100 | Obstetric breast infection NOS - delivered |
| L45z200 | Obstetric breast infection NOS - deliv with p/n complication |
| L45z300 | Obstetric breast infection NOS with antenatal complication |
| L45z400 | Obstetric breast infection NOS with postnatal complication |
| L45zz00 | Obstetric breast infection NOS |
| L46..00 | Obstetric breast and lactation disorders NOS |
| L46..11 | Lactation problems |
| L460.00 | Retracted nipple in pregnancy, the puerperium or lactation |
| L460000 | Retracted nipple in pregnancy/puerperium/lactation unspec |
| L460100 | Retracted nipple in pregnancy/puerperium/lactation - deliv |
| L460200 | Retracted nipple in pregnancy/puerp/lact - deliv + p/n comp |
| L460300 | Retracted nipple in pregnancy/puerperium/lact with a/n comp |
| L460400 | Retracted nipple in pregnancy/puerperium/lact with p/n comp |
| L460z00 | Retracted nipple in pregnancy/puerperium/lactation NOS |
| L461.00 | Cracked nipple in pregnancy, the puerperium or lactation |
| L461.11 | Fissure of nipple |
| L461000 | Cracked nipple in pregnancy/puerperium/lactation unspecified |
| L461100 | Cracked nipple in pregnancy/puerperium/lactation - delivered |
| L461200 | Cracked nipple in pregnancy/puerp/lact - deliv + p/n comp |
| L461300 | Cracked nipple in pregnancy/puerperium/lactation + a/n comp |
| L461400 | Cracked nipple in pregnancy/puerperium/lactation + p/n comp |
| L461z00 | Cracked nipple in pregnancy, the puerperium or lactation NOS |
| L462.00 | Breast engorgement in pregnancy, the puerperium or lactation |
| L462000 | Breast engorgement in pregnancy/puerperium/lactation unspec |
| L462100 | Breast engorgement in pregnancy/puerperium/lactation - deliv |
| L462200 | Breast engorgement in pregnancy/puerp/lact - del + p/n comp |
| L462300 | Breast engorgement in pregnancy/puerperium/lact + a/n comp |
| L462400 | Breast engorgement in pregnancy/puerperium/lact + p/n comp |
| L462z00 | Breast engorgement in pregnancy/puerperium/lactation NOS |
| L462z11 | Breast engorgement |
| L463.00 | Other breast disorder in pregnancy/puerperium/lactation |
| L463000 | Other breast disorder in pregnancy/puerperium/lact unspec |
| L463100 | Other breast disorder in pregnancy/puerperium/lact - deliv |
| L463200 | Other breast disorder in pregnancy/puerperium/lact +p/n comp |
| L463300 | Other breast disorder in pregnancy/puerperium/lact +a/n comp |
| L463400 | Other breast disorder in pregnancy/puerperium/lact +p/n comp |
| L463500 | Pain on breast feeding |
| L463z00 | Other breast disorder in pregnancy/puerperium/lactation NOS |
| L464.00 | Failure of lactation |
| L464.11 | Agalactia |
| L464000 | Failure of lactation unspecified |
| L464100 | Failure of lactation - delivered |
| L464200 | Failure of lactation - delivered with postnatal complication |
| L464300 | Failure of lactation with antenatal complication |
| L464400 | Failure of lactation with postnatal complication |
| L464z00 | Failure of lactation NOS |
| L465.00 | Suppressed lactation |
| L465000 | Suppressed lactation unspecified |
| L465100 | Suppressed lactation - delivered |
| L465200 | Suppressed lactation - delivered with postnatal complication |
| L465300 | Suppressed lactation with antenatal complication |
| L465400 | Suppressed lactation with postnatal complication |
| L465z00 | Suppressed lactation NOS |
| L466.00 | Galactorrhoea in pregnancy and the puerperium |
| L466000 | Galactorrhoea in pregnancy and the puerperium unspecified |
| L466100 | Galactorrhoea in pregnancy and the puerperium - delivered |
| L466200 | Galactorrhoea in pregnancy/puerperium - deliv with p/n comp |
| L466300 | Galactorrhoea in pregnancy/puerperium with a/n complication |
| L466400 | Galactorrhoea in pregnancy/puerperium with p/n complication |
| L466z00 | Galactorrhoea in pregnancy and the puerperium NOS |
| L467.00 | Hypogalactia |
| L46y.00 | Other disorders of lactation |
| L46y.11 | Galactocele - obstetric |
| L46y000 | Other disorder of lactation unspecified |
| L46y100 | Other disorder of lactation - delivered |
| L46y200 | Other disorder of lactation - delivered with p/n comp |
| L46y300 | Other disorder of lactation with antenatal complication |
| L46y400 | Other disorder of lactation with postnatal complication |
| L46yz00 | Other disorder of lactation NOS |
| L46z.00 | Disorders of lactation NOS |
| L46z000 | Disorder of lactation NOS, unspecified |
| L46z100 | Disorder of lactation NOS - delivered |
| L46z200 | Disorder of lactation NOS - delivered with p/n complication |
| L46z300 | Disorder of lactation NOS with antenatal complication |
| L46z400 | Disorder of lactation NOS with postnatal complication |
| L46zz00 | Disorder of lactation NOS |
| L4y..00 | Other specified complications of the puerperium |
| L4z..00 | Complications of the puerperium NOS |
| L5...00 | Maternal care for fetus |
| L50..00 | Maternal care for compound presentation |
| L51..00 | Maternal care for other known or suspected fetal problems |
| L510.00 | Maternal care for hydrops fetalis |
| L511.00 | Maternal care for viable fetus in abdominal pregnancy |
| L512.00 | Maternal care for diminished fetal movements |
| L514.00 | Maternal care for poor fetal growth |
| L51X.00 | Maternal care/known or suspected fetal problem,unspecifd |
| Ly...00 | Complications of pregnancy,childbirth or the puerperium OS |
| Ly0..00 | Spontaneous vertex delivery |
| Ly1..00 | Spontaneous breech delivery |
| Ly2..00 | Sequelae of complication pregnancy childbirth and puerperium |
| Lyu..00 | [X]Additional preg,cldbirth+puerperium diseas clssfctn terms |
| Lyu0.00 | [X]Pregnancy with abortive outcome |
| Lyu0000 | [X]Other ectopic pregnancy |
| Lyu0100 | [X]Other specified abnormal products of conception |
| Lyu0200 | [X]Other abortion |
| Lyu0300 | [X]Failed medical abortion,wth other+unspcfied complications |
| Lyu0400 | [X]Oth+unspcf failed inducd abort,complct gen tract+pelv inf |
| Lyu0500 | [X]Oth+unspc fail induc abortn,complict/delay/exces h'morrhg |
| Lyu0600 | [X]Other+unspcf failed induced abortion,complicated/embolism |
| Lyu0700 | [X]Oth+unspcf failed inducd abortn,wth oth+unspcf complicatn |
| Lyu0800 | [X]Other+unspcf failed induced abortion,without complication |
| Lyu0900 | [X]Oth venous complicatns follow abortn+ectopic+molr pregncy |
| Lyu0A00 | [X]Other complications follow abortn+ectopic+molar pregnancy |
| Lyu0B00 | [X]Complic following abortion & ectopic & molar preg, unspec |
| Lyu1.00 | [X]Oedema,proteinuria+hypertens in pregnancy,childbrth,puerp |
| Lyu2.00 | [X]Other maternal disorders predominant related to pregnancy |
| Lyu2000 | [X]Other haemorrhage in early pregnancy |
| Lyu2100 | [X]Other vomiting complicating pregnancy |
| Lyu2200 | [X]Other venous complications in pregnancy |
| Lyu2300 | [X]Infections of other parts of urinary tract in pregnancy |
| Lyu2400 | [X]Other+unspcf genitourinary tract infection in pregnancy |
| Lyu2500 | [X]Other specified pregnancy-related conditions |
| Lyu2600 | [X]Other abnormal findings on antenatal screening of mother |
| Lyu2700 | [X]Oth complicatns/spinal+epidural anaesthsia during pregncy |
| Lyu2800 | [X]Other complications of anaesthesia during pregnancy |
| Lyu2900 | [X]Pre-existing diabetes mellitus, unspecified |
| Lyu2A00 | [X]Abnormal finding on antenatal screening of mother |
| Lyu3.00 | [X]Maternal care relat to fetus+amniotic cavity+deliv prob |
| Lyu3000 | [X]Other multiple gestation |
| Lyu3100 | [X]Other complications specific to multiple gestation |
| Lyu3200 | [X]Maternal care for other malpresentation of fetus |
| Lyu3300 | [X]Maternal care for other abnormalities of cervix |
| Lyu3400 | [X]Maternal care for other abnormalities of gravid uterus |
| Lyu3500 | [X]Maternal care for other abnormalities of pelvic organs |
| Lyu3600 | [X]Maternal care/(suspected)damage/fetus/oth medicl procedur |
| Lyu3700 | [X]Maternal care/other(suspected)fetal abnormality+damage |
| Lyu3800 | [X]Maternal care for other isoimmunization |
| Lyu3900 | [X]Maternal care/oth spcf known or suspected fetal problems |
| Lyu3A00 | [X]Maternal care/known or suspected fetal problem,unspecifd |
| Lyu3B00 | [X]Other disorders of amniotic fluid and membranes |
| Lyu3C00 | [X]Other placental disorders |
| Lyu3D00 | [X]Other premature separation of placenta |
| Lyu3E00 | [X]Other antepartum haemorrhage |
| Lyu4.00 | [X]Complications of labour and delivery |
| Lyu4000 | [X]Other failed induction of labour |
| Lyu4100 | [X]Other uterine inertia |
| Lyu4200 | [X]Other abnormalities of forces of labour |
| Lyu4300 | [X]Obstructed labour due/other malposition+malpresentation |
| Lyu4400 | [X]Obstructd labour due to oth maternal pelvic abnormalities |
| Lyu4500 | [X]Obstructed labour due to other abnormalities of fetus |
| Lyu4600 | [X]Other specified obstructed labour |
| Lyu4700 | [X]Other intrapartum haemorrhage |
| Lyu4800 | [X]Labour+delivery complicat/oth evidence of fetal distress |
| Lyu4900 | [X]Labour+delivery complicated by other cord entanglement |
| Lyu4A00 | [X]Labour+delivery complicated by other cord complications |
| Lyu4B00 | [X]Other obstetric injury to pelvic organs |
| Lyu4C00 | [X]Other specified obstetric trauma |
| Lyu4D00 | [X]Other immediate postpartum haemorrhage |
| Lyu4E00 | [X]Oth pulmonary complicatns/anaesthesia during lab+delivery |
| Lyu4F00 | [X]Oth complicatn/spinl+epidur anaesths during lab+delivery |
| Lyu4G00 | [X]Other complications of anaesthesia during labour+delivery |
| Lyu4H00 | [X]Other infection during labour |
| Lyu4J00 | [X]Other complications of obstetric surgery and procedures |
| Lyu4K00 | [X]Other specified complications of labour and delivery |
| Lyu4L00 | [X]Obstructed labour due to fetopelv disproportion, unspec |
| Lyu4M00 | [X]Intrapartum haemorrhage, unspecified |
| Lyu4N00 | [X]Labour & delivery complicated by fetal stress, unspecif |
| Lyu4P00 | [X]Complication of anaesthesia during labour and deliv unsp |
| Lyu5.00 | [X]Delivery |
| Lyu5000 | [X]Other single spontaneous delivery |
| Lyu5100 | [X]Other and unspecified forceps delivery |
| Lyu5200 | [X]Other single delivery by caesarean section |
| Lyu5300 | [X]Other assisted breech delivery |
| Lyu5400 | [X]Other manipulation-assisted delivery |
| Lyu5500 | [X]Other specified assisted single delivery |
| Lyu5600 | [X]Other multiple delivery |
| Lyu5700 | [X]Assisted single delivery, unspecified |
| Lyu5800 | [X]Multiple delivery, unspecified |
| Lyu6.00 | [X]Complications predominantly related to the puerperium |
| Lyu6000 | [X]Other infection of genital tract following delivery |
| Lyu6100 | [X]Other genitourinary tract infections following delivery |
| Lyu6200 | [X]Other specified puerperal infection |
| Lyu6300 | [X]Other venous complications in the puerperium |
| Lyu6400 | [X]Other obstetric embolism |
| Lyu6500 | [X]Oth complicatn/spinal+epidural anaesthes during puerperum |
| Lyu6600 | [X]Other complications of anaesthesia during the puerperium |
| Lyu6700 | [X]Other specified puerperal complications |
| Lyu6800 | [X]Other+unspcf disorders/breast associated with childbirth |
| Lyu6900 | [X]Other and unspecified disorders of lactation |
| Lyu6A00 | [X]Infection of caesarean section wound following delivery |
| Lyu6B00 | [X]Vaginitis following delivery |
| Lyu6C00 | [X]Cervicitis following delivery |
| Lyu7.00 | [X]Other obstetric conditions, not elsewhere classified |
| Lyu7000 | [X]Oth infctns wth predomin sexual mode/transmissn complicat |
| Lyu7100 | [X]Other viral diseases complicating preg,cldbirth+puerperum |
| Lyu7200 | [X]Oth infects+parasitc dis complicat preg,cldbrth+puerperum |
| Lyu7300 | [X]Oth d/bld+bld-form org+c d inv im mch cm preg,cldbir+puer |
| Lyu7400 | [X]Oth spcf dis+conditns complicat preg,childbirth+puerperum |
| Lyu7500 | [X]Obstetric death of unspecified cause |
| Lz...00 | Complications of pregnancy,childbirth and the puerperium NOS |

1. Smoking codes

| Read code | Description | Smoking status |
| --- | --- | --- |
| 13p0.00 | Negotiated date for cessation of smoking | smoker |
| ZRh4.11 | RFS - Reasons for smoking scale | smoker |
| 137N.00 | Ex pipe smoker | ex-smoker |
| 9kn..00 | Non-smoker annual review - enhanced services administration | never-smoker |
| 137Q.11 | Smoking restarted | smoker |
| 137A.00 | Ex-heavy smoker (20-39/day) | ex-smoker |
| 6791.00 | Health ed. - smoking | smoker |
| 137j.00 | Ex-cigarette smoker | ex-smoker |
| ZRao.00 | Occasions for smoking scale | smoker |
| 67A3.00 | Pregnancy smoking advice | smoker |
| 8CAL.00 | Smoking cessation advice | smoker |
| E251z00 | Tobacco dependence NOS | smoker |
| 137V.00 | Smoking reduced | smoker |
| ZG23300 | Advice on smoking | smoker |
| 137l.00 | Ex roll-up cigarette smoker | ex-smoker |
| 9ko..00 | Current smoker annual review - enhanced services admin | smoker |
| 8IEK.00 | Smoking cessation programme declined | smoker |
| 137P.11 | Smoker | smoker |
| ZRao.11 | OFS - Occasions for smoking scale | smoker |
| 137H.00 | Pipe smoker | smoker |
| 1373.00 | Light smoker - 1-9 cigs/day | smoker |
| 9kf2.11 | COPD structured smoking assessment declined | smoker |
| 8IEo.00 | Referral to smoking cessation service declined | smoker |
| 1372.00 | Trivial smoker - < 1 cig/day | smoker |
| 137f.00 | Reason for restarting smoking | smoker |
| 9ko..11 | Current smoker annual review | smoker |
| 137..11 | Smoker - amount smoked | smoker |
| 137h.00 | Minutes from waking to first tobacco consumption | smoker |
| 8T08.00 | Referral to smoking cessation service | smoker |
| E251200 | Tobacco dependence, episodic | smoker |
| 137d.00 | Not interested in stopping smoking | smoker |
| 8IEM.00 | Smoking cessation drug therapy declined | smoker |
| 137m.00 | Failed attempt to stop smoking | smoker |
| 8H7i.00 | Referral to smoking cessation advisor | smoker |
| 137e.00 | Smoking restarted | smoker |
| 137Q.00 | Smoking started | smoker |
| 8HkQ.00 | Referral to NHS stop smoking service | smoker |
| ZRh4.00 | Reasons for smoking scale | smoker |
| 1376.00 | Very heavy smoker - 40+cigs/d | smoker |
| E251000 | Tobacco dependence, unspecified | smoker |
| ZRaM.00 | Motives for smoking scale | smoker |
| 137K000 | Recently stopped smoking | ex-smoker |
| 1377.00 | Ex-trivial smoker (<1/day) | ex-smoker |
| 137b.00 | Ready to stop smoking | smoker |
| 8IAj.00 | Smoking cessation advice declined | smoker |
| 9kf1.11 | Referred for COPD structured smoking assessment | smoker |
| 1378.00 | Ex-light smoker (1-9/day) | ex-smoker |
| 8IEM000 | Varenicline smoking cessation therapy declined | smoker |
| 1379.00 | Ex-moderate smoker (10-19/day) | ex-smoker |
| 9NS0200 | Referral for smoking cessation service offered | smoker |
| 67H1.00 | Lifestyle advice regarding smoking | smoker |
| 9kf1.00 | Refer COPD structured smoking assessment - enhanc serv admin | smoker |
| 1371.11 | Non-smoker | never-smoker |
| 137C.00 | Keeps trying to stop smoking | smoker |
| 1372.11 | Occasional smoker | smoker |
| 137J.00 | Cigar smoker | smoker |
| 1V08.00 | Smokes drugs in cigarette form | smoker |
| E251300 | Tobacco dependence in remission | ex-smoker |
| 137P.00 | Cigarette smoker | smoker |
| 137B.00 | Ex-very heavy smoker (40+/day) | ex-smoker |
| 137R.00 | Current smoker | smoker |
| ZV4K000 | [V]Tobacco use | smoker |
| 137G.00 | Trying to give up smoking | smoker |
| 8HTK.00 | Referral to stop-smoking clinic | smoker |
| 9kf2.00 | COPD structured smoking assessment declined - enh serv admin | smoker |
| 67H6.00 | Brief intervention for smoking cessation | smoker |
| 137c.00 | Thinking about stopping smoking | smoker |
| 1374.00 | Moderate smoker - 10-19 cigs/d | smoker |
| SMC..00 | Toxic effect of tobacco and nicotine | smoker |
| 1375.00 | Heavy smoker - 20-39 cigs/day | smoker |
| ZRaM.11 | MFS - Motives for smoking scale | smoker |
| 137O.00 | Ex cigar smoker | ex-smoker |
| E251100 | Tobacco dependence, continuous | smoker |
| 137T.00 | Date ceased smoking | ex-smoker |
| 9km..11 | Ex-smoker annual review | ex-smoker |
| 9kn..11 | Non-smoker annual review | never-smoker |
| 9km..00 | Ex-smoker annual review - enhanced services administration | ex-smoker |
| 137M.00 | Rolls own cigarettes | smoker |
| 1371.00 | Never smoked tobacco | never-smoker |
| 137F.00 | Ex-smoker - amount unknown | ex-smoker |
| E251.00 | Tobacco dependence | smoker |
| 8CAg.00 | Smoking cessation advice provided by community pharmacist | smoker |
| 137S.00 | Ex smoker | ex-smoker |
| 137K.00 | Stopped smoking | ex-smoker |

1. Conditions precluding the use of contraception codes

| Read code | Description |
| --- | --- |
| 1599.00 | H/O: hysterectomy |
| 159A.11 | H/O: sterilisation - female |
| 159B.00 | H/O: bilateral oophorectomy |
| 15A9.11 | H/O: hysterotomy |
| 61H..00 | Contraception: female sterilis |
| 685H.00 | No smear - benign hysterectomy |
| 685H.11 | No smear - hysterectomy |
| 7E04.00 | Abdominal excision of uterus |
| 7E04.11 | Abdominal hysterectomy |
| 7E04.12 | Wertheim hysterectomy |
| 7E04000 | Abdominal hysterocolpectomy and excision periuterine tissue |
| 7E04100 | Abdominal hysterectomy & excision of periuterine tissue NEC |
| 7E04200 | Abdominal hysterocolpectomy NEC |
| 7E04300 | Total abdominal hysterectomy NEC |
| 7E04311 | Bonney abdominal hysterectomy |
| 7E04312 | Hysterectomy NEC |
| 7E04400 | Subtotal abdominal hysterectomy |
| 7E04500 | Abdominal hysterectomy and bilateral salpingoophorectomy |
| 7E04511 | Abdominal hysterectomy & bilateral salpingoophorectomy (BSO) |
| 7E04512 | TAH - total abdom hysterectomy & bilateral salpingoophorect |
| 7E04600 | Radical hysterectomy |
| 7E04700 | Abdominal hysterectomy and right salpingoopherectomy |
| 7E04711 | Abdominal hysterectomy and left salpingoopherectomy |
| 7E04800 | Abdominal hysterectomy and left salpingoophorectomy |
| 7E04900 | TAH - Tot abdom hysterectomy and BSO - bilat salpingophorect |
| 7E04A00 | Abdominal hysterectomy with conservation of ovaries |
| 7E04B00 | Lapar total abdominal hysterect bilat salpingo-oophorectomy |
| 7E04C00 | Laparoscopic hysterectomy |
| 7E04E00 | Laparoscopic subtotal hysterectomy |
| 7E04F00 | Subtotal abdominal hysterectomy with conservation of ovaries |
| 7E04G00 | Total abdominal hysterectomy with conservation of ovaries |
| 7E04H00 | Subtotl abdominal hysterectomy & bilat salpingo-oophorectomy |
| 7E04J00 | Subtotl abdominal hysterectomy & right salpingo-oophorectomy |
| 7E04K00 | Subtotal abdominal hysterectomy & left salpingo-oophorectomy |
| 7E04N00 | Radical hysterectomy with conservation of ovaries |
| 7E04P00 | Radical hysterectomy with bilateral salpingo-oophorectomy |
| 7E04y00 | Other specified abdominal excision of uterus |
| 7E04z00 | Abdominal excision of uterus NOS |
| 7E05.00 | Vaginal excision of uterus |
| 7E05.11 | Schauta radical vaginal hysterectomy |
| 7E05.12 | Vaginal hysterectomy |
| 7E05000 | Vaginal hysterocolpectomy and excision of periuterine tissue |
| 7E05100 | Vaginal hysterectomy and excision of periuterine tissue NEC |
| 7E05200 | Vaginal hysterocolpectomy NEC |
| 7E05300 | Vaginal hysterectomy NEC |
| 7E05311 | Heaney vaginal hysterectomy |
| 7E05400 | Laparoscopic vaginal hysterectomy |
| 7E05500 | Vaginal hysterectomy with conservation of ovaries |
| 7E05600 | Lap assist vag hysterectomy with bilat salpingo-oophorectomy |
| 7E05700 | Vaginal hysterectomy and right salpingo-oophorectomy |
| 7E05800 | Vaginal hysterectomy and left salpingo-oophorectomy |
| 7E05y00 | Other specified vaginal excision of uterus |
| 7E05y11 | Ward vaginal hysterectomy |
| 7E05z00 | Vaginal excision of uterus NOS |
| 7E10000 | Bilateral salpingoophorectomy |
| 7E10100 | Bilateral salpingectomy NEC |
| 7E10200 | Bilateral oophorectomy NEC |
| 7E11100 | Salpingoophorectomy remaining solitary fallop tube and ovary |
| 7E11300 | Salpingectomy of remaining solitary fallopian tube NEC |
| 7E11500 | Oophorectomy of remaining solitary ovary NEC |
| 7E15.00 | Open bilateral occlusion of fallopian tubes |
| 7E15.11 | Open bilateral female sterilisation |
| 7E15000 | Open bilateral ligation of fallopian tubes |
| 7E15011 | Pomeroy open bilateral ligation of fallopian tubes |
| 7E15100 | Open bilateral clipping of fallopian tubes |
| 7E15111 | Open bilateral ringing of fallopian tubes |
| 7E15y00 | Other specified open bilateral occlusion of fallopian tubes |
| 7E15z00 | Open bilateral occlusion of fallopian tubes NOS |
| 7E16.11 | Other open female sterilisation |
| 7E16000 | Open ligation of remaining solitary fallopian tube |
| 7E16200 | Open clipping of remaining solitary fallopian tube |
| 7E16211 | Open clipping of residual solitary fallopian tube |
| 7E16212 | Open ringing of remaining solitary fallopian tube |
| 7E1C.00 | Endoscopic bilateral occlusion of fallopian tubes |
| 7E1C.11 | Endoscopic bilateral female sterilisation |
| 7E1C.12 | Laparoscopic bilateral female sterilisation |
| 7E1C000 | Endoscopic bilateral cauterisation of fallopian tubes |
| 7E1C100 | Endoscopic bilateral clipping of fallopian tubes |
| 7E1C200 | Endoscopic bilateral ringing of fallopian tubes |
| 7E1C300 | Endoscopic bilateral placement of intrafallopian implants |
| 7E1Cy00 | Endoscopic bilateral occlusion of fallopian tubes OS |
| 7E1Cz00 | Endoscopic bilateral occlusion of fallopian tubes NOS |
| 7E1D.12 | Other endoscopic female sterilisation |
| 7E1D.13 | Other laparoscopic female sterilisation |
| 7E1D000 | Endoscopic occlusion of remaining solitary fallopian tube |
| 7E1D300 | Endo place intrafallop implant remain solitary fallop tube |
| 7F1A000 | Caesarean hysterectomy |
| 9O8W.00 | Cervical smear to continue post hysterectomy |
| K515.00 | Post hysterectomy vaginal vault prolapse |
| L398500 | Delivery by caesarean hysterectomy |
| ZV25200 | [V]Sterilisation |

1. LARC insertion/administration codes

| Read code | Description |
| --- | --- |
| 6151.00 | IUD fitted |
| 6153.00 | IUD re-fitted |
| 61A2.00 | "Morning after" IUD fitted |
| 61A2.11 | Post-coital IUD fitted |
| 7E09.12 | Intrauterine device procedure |
| 7E09000 | Introduction of intrauterine contraceptive device |
| 7E09011 | Fitting of intrauterine contraceptive device |
| 7E09100 | Replacement of intrauterine contraceptive device |
| 7E09111 | Change of intrauterine contraceptive device |
| 7E09400 | Introduction of Mirena coil |
| 7E09600 | Replacement of intrauterine system |
| 7E09700 | Insertion of intrauterine system |
| ZV25100 | [V]Intrauterine contraceptive device insertion |
| ZV25112 | [V]Intrauterine contraceptive device insertion |
| ZV25113 | [V]Intrauterine contraceptive device insertion |
| ZV25412 | [V]Reinsertion of coil |
| ZV25415 | [V]Reinsertion of intrauterine contraceptive device |
| ZV2541A | [V]Reinsertion of intrauterine contraceptive device |
| ZV25D00 | [V]Reinsertion of intrauterine contraceptive device |
| ZV25D11 | [V]Reinsertion of coil |
| 7G2AG00 | Insertion of Implanon |
| 7G2AJ00 | Insertion of etonogestrel radiopaque contraceptive implant |
| 61KA.00 | Insertion of subcutaneous contraceptive |
| 61KC.00 | Insert subcutaneous contraceptive implnt othr healthcre prov |
| 7G2AB00 | Insertion of subcutaneous contraceptive |
| 7G2AH00 | Reinsertion of subcutaneous contraceptive |
| 61B..00 | Depot contraceptive |
| 61B..11 | Depot contraception |
| 61B1.00 | Depot contraceptive given |
| 61B1.11 | Depo-provera injection given |
| 61B2.00 | Depot contraceptive repeated |
| 61B3.00 | Depot contraceptive-no problem |
| 61BZ.00 | Depot contraceptive NOS |

1. LARC removal codes

| Read code | Description |
| --- | --- |
| 6152.00 | IUD removed |
| 615B.00 | IUD expelled |
| 615B.11 | IUD fallen out |
| 7D1E400 | Removal intrauterine contracept device from pouch of Douglas |
| 7D1E411 | Removal of contraceptive coil from pouch of Douglas |
| 7E09200 | Removal of intrauterine contraceptive device NEC |
| 7E09300 | Removal of displaced intrauterine contraceptive device |
| 7E09500 | Removal of Mirena coil |
| 7E09800 | Removal of intrauterine system |
| ZV25413 | [V]Removal of coil |
| ZV25416 | [V]Removal of intrauterine contraceptive device |
| ZV2541B | [V]Removal of intrauterine contraceptive device |
| ZV25E00 | [V]Removal of intrauterine contraceptive device |
| ZV25E11 | [V]Removal of coil |
| 61KF.00 | Remov subcutaneous contraceptive implant othr healthcre prov |
| 7G2HA00 | Removal of Implanon |
| 7G2HB00 | Removal of etonogestrel radiopaque contraceptive implant |
| 7G2H700 | Removal of subcutaneous contraceptive |
