# Supplementary material for: Use of contraceptives and risk of inflammatory bowel disease: a nested case–control study
Source: Aliment Pharmacol Ther. Author manuscript; Available in PMC 2022 Jun 28. (PMC7612921; doi:10.1111/apt.16647)
Supplement: Supporting Information Tables [file EMS146182-supplement-Supporting_Information_Tables.docx]

## Supporting information - tables:

Supporting information table 1. Numbers, proportions and adjusted odds ratios for Crohn’s disease and ulcerative colitis by contraceptive exposure.

|  | **Crohn’s disease** | | | **Ulcerative colitis** | | |
| --- | --- | --- | --- | --- | --- | --- |
|  | Cases n(%) n=2,231 | Controls n(%)  n=13,279 | Adjusted  odds ratio  (95% CI) † | Cases n(%)  n=2,701 | Controls n(%)  n=16,061 | Adjusted  odds ratio  (95% CI) † |
| **Non-user** | 659 (29.5) | 5,204 (39.2) | 1 | 921 (34.1) | 6,643 (41.4) | 1 |
| **2nd generation COCP user** | 622 (27.9) | 2,819 (21.2) | 1.69 (1.48-1.93) | 573 (21.2) | 3,008 (18.7) | 1.27 (1.12-1.44) |
| **Newer generation COCP user** | 117 (5.2) | 728 (5.5) | 1.25 (1.01-1.57) | 165 (6.1) | 822 (5.1) | 1.38 (1.14-1.67) |
| **Progestogen-only pill user** | 81 (3.6) | 537 (4.0) | 1.09 (0.84-1.40) | 138 (5.1) | 760 (4.7) | 1.25 (1.03-1.53) |
| **Parenteral method user** | 150 (6.7) | 879 (6.6) | 1.21 (0.99-1.47) | 195 (7.2) | 1,166 (7.3) | 1.17 (0.98-1.39) |
| **Mixed user** | 602 (27.0) | 3,112 (23.4) | 1.50 (1.30-1.72) | 709 (26.3) | 3,662 (22.8) | 1.31 (1.15-1.48) |

†Adjusted for social deprivation by Townsend score, smoking status, BMI, history of polycystic ovarian syndrome, history of endometriosis, history of acne, history of pregnancy. All odds ratios are generated using non-use as the reference group

| *Supporting information table 2. Interaction coefficients (adjusted OR*†*) for smoking & progestogen-only pill exposure interactions* | | |
| --- | --- | --- |
| Smoking status | CD (95% CI) p= 0.12 | UC (95% CI) p=0.13 |
| Never smoker | 0.98 (0.94-1.02) | 1.00 (0.97-1.04) |
| Ex-smoker | 0.98 (0.91-1.06) | 1.00 (0.94-1.05) |
| Current smoker | 0.99 (0.95-1.04) | 1.06 (1.01-1.10) |
| Missing | 1.10 (1.00-1.19) | 0.98 (0.88-1.09) |
| † Adjusted for social deprivation by Townsend score, smoking status, body mass index, history of polycystic ovarian syndrome, history of endometriosis, history of acne, history of pregnancy | | |

| *Supporting information table 3. Interaction coefficients (adjusted OR*†*) for smoking & combined oral contraceptive pill exposure interactions* | | |
| --- | --- | --- |
| Smoking status | CD (95% CI) p= 0.35 | UC (95% CI) p= 0.03 |
| Never smoker | 1.05 (1.04-1.07) | 1.04 (1.02-1.05) |
| Ex-smoker | 1.07 (1.04-1.10) | 1.01 (0.99-1.04) |
| Current smoker | 1.07 (1.05-1.09) | 1.01 (0.99-1.04) |
| Missing | 1.08 (1.05-1.11) | 1.07 (1.04-1.10) |
| †Adjusted for social deprivation by Townsend score, smoking status, body mass index, history of polycystic ovarian syndrome, history of endometriosis, history of acne, history of pregnancy | | |

| *Supporting information table 4. Interaction coefficients (adjusted OR*†*) for smoking & ‘any oral contraceptive pill’ exposure interactions* | | |
| --- | --- | --- |
| Smoking status | CD (95% CI) p= 0.17 | UC (95% CI) p= 0.11 |
| Never smoker | 1.05 (1.03-1.06) | 1.03 (1.02-1.05) |
| Ex-smoker | 1.06 (1.03-1.09) | 1.01 (0.99-1.04) |
| Current smoker | 1.06 (1.04-1.08) | 1.03 (1.00-1.05) |
| Missing | 1.08 (1.05-1.12) | 1.06 (1.03-1.09) |
| †Adjusted for social deprivation by Townsend score, smoking status, body mass index, history of polycystic ovarian syndrome, history of endometriosis, history of acne, history of pregnancy | | |

*Supporting information table 5. Adjusted odds ratios for Crohn’s disease and ulcerative colitis by any OCP exposure. Results are stratified by calendar period in five-yearly quantiles*

|  | **Crohn’s disease** | | | **Ulcerative colitis** | | |
| --- | --- | --- | --- | --- | --- | --- |
|  | Cases n(%)  n=2,231 | Controls n(%)  N=13,279 | Adjusted  odds ratio  (95% CI) † | Cases n(%) n=2,701 | Controls n(%) n=16,061 | Adjusted  odds ratio  (95% CI) † |
| **2000-2004** | 427 (19.1) | 2,538 (19.1) | 1.51 (1.15-1.95) | 524 (19.4) | 3,134 (19.5) | 1.29 (1.02-1.63) |
| **2005-2009** | 749 (33.6) | 4,470 (33.7) | 1.62 (1.30-2.00) | 856 (31.7) | 5,107 (31.8) | 1.20 (0.99-1.46) |
| **2010-2014** | 710 (31.8) | 4,214 (31.7) | 1.36 (1.09-1.70) | 858 (31.8) | 5,075 (31.6) | 1.30 (1.06-1.58) |
| **2015-2018** | 345 (15.5) | 2,057 (15.5) | 1.38 (0.99-1.95) | 463 (17.1) | 2,745 (17.1) | 1.40 (1.05-1.87) |

†Adjusted for social deprivation by Townsend score, smoking status, BMI, history of polycystic ovarian syndrome, history of endometriosis, history of acne, history of pregnancy. All odds ratios are generated using non-use as the reference group

Supporting information table 6. Numbers, proportions and adjusted odds ratios for Crohn’s disease and ulcerative colitis by contraceptive exposure. Sensitivity analysis – women with missing BMI excluded and BMI treated as a continuous variable

|  | **Crohn’s disease** | | | **Ulcerative colitis** | | |
| --- | --- | --- | --- | --- | --- | --- |
|  | Cases n(%) n=1,861 | Controls n(%)  n=10,016 | Adjusted  odds ratio  (95% CI) † | Cases n(%)  n=2,297 | Controls n(%)  n=12,862 | Adjusted  odds ratio  (95% CI) † |
| **Non-user** | 496 (26.7) | 3,220 (32.2) | 1 | 712 (31.0) | 4,578 (35.6) | 1 |
| **2nd generation COCP user** | 523 (28.1) | 2,259 (22.6) | 1.57 (1.34-1.83) | 500 (21.8) | 2,534 (19.7) | 1.24 (1.08-1.42) |
| **Newer generation COCP user** | 99 (5.3) | 608 (6.1) | 1.11 (0.87-1.44) | 149 (6.5) | 713 (5.5) | 1.31 (1.06-1.61) |
| **Progestogen-only pill user** | 71 (3.8) | 456 (4.6) | 0.96 (0.73-1.26) | 125 (5.4) | 680 (5.3) | 1.27 (1.03-1.58) |
| **Parenteral method user** | 129 (6.9) | 757 (7.6) | 1.06 (0.85-1.33) | 175 (7.6) | 1,018 (7.9) | 1.15 (0.95-1.38) |
| **Mixed user** | 543 (29.2) | 2,716 (27.1) | 1.37 (1.16-1.60) | 636 (27.7) | 3,339 (26.0) | 1.23 (1.07-1.41) |

†Adjusted for social deprivation by Townsend score, smoking status, BMI, history of polycystic ovarian syndrome, history of endometriosis, history of acne, history of pregnancy. All odds ratios are generated using non-use as the reference group
